# Critical Review and Conceptual and Quantitative Models for the Transfer and Depuration of Ciguatoxins in Fishes

**DOI:** 10.3390/toxins13080515

**Published:** 2021-07-23

**Authors:** Michael J. Holmes, Bill Venables, Richard J. Lewis

**Affiliations:** 1 Queensland Department of Environment and Science, Brisbane 4102, Australia; michael.holmes@des.qld.gov.au; 2 CSIRO Data61, Brisbane 4102, Australia; bill.venables@gmail.com; 3 Institute for Molecular Bioscience, The University of Queensland, Brisbane 4072, Australia

**Keywords:** ciguatera, ciguatoxin, maitotoxin, 44-methylgambierone, toxin depuration, *Gambierdiscus*, *Fukuyoa*, Platypus Bay, Great Barrier Reef, *Scomberomorus commerson*, Spanish mackerel, *Plectropomus*, coral trout, turf algae, surgeonfish, *Ctenochaetus*, *Acanthurus*

## Abstract

We review and develop conceptual models for the bio-transfer of ciguatoxins in food chains for Platypus Bay and the Great Barrier Reef on the east coast of Australia. Platypus Bay is unique in repeatedly producing ciguateric fishes in Australia, with ciguatoxins produced by benthic dinoflagellates (*Gambierdiscus* spp.) growing epiphytically on free-living, benthic macroalgae. The *Gambierdiscus* are consumed by invertebrates living within the macroalgae, which are preyed upon by small carnivorous fishes, which are then preyed upon by Spanish mackerel (*Scomberomorus commerson*). We hypothesise that *Gambierdiscus* and/or *Fukuyoa* species growing on turf algae are the main source of ciguatoxins entering marine food chains to cause ciguatera on the Great Barrier Reef. The abundance of surgeonfish that feed on turf algae may act as a feedback mechanism controlling the flow of ciguatoxins through this marine food chain. If this hypothesis is broadly applicable, then a reduction in herbivory from overharvesting of herbivores could lead to increases in ciguatera by concentrating ciguatoxins through the remaining, smaller population of herbivores. Modelling the dilution of ciguatoxins by somatic growth in Spanish mackerel and coral trout (*Plectropomus leopardus*) revealed that growth could not significantly reduce the toxicity of fish flesh, except in young fast-growing fishes or legal-sized fishes contaminated with low levels of ciguatoxins. If Spanish mackerel along the east coast of Australia can depurate ciguatoxins, it is most likely with a half-life of ≤1-year. Our review and conceptual models can aid management and research of ciguatera in Australia, and globally.

## 1. Introduction

Ciguatera is a disease in humans caused by eating normally edible warm water fishes contaminated with a class of potent, lipid-soluble toxins called ciguatoxins (CTX). It is estimated to poison >25,000 people annually [1], with ~500,000 people poisoned across the Pacific basin between 1973 and 2008 [2]. The largest single outbreak of ciguatera possibly occurred in 1748 in the invasion fleet of British Admiral Boscawen at Rodrigues Island in the Indian Ocean, when >1500 men reportedly died before his failed assault on the then French island of Mauritius [3]. Until recently, ciguatera was mostly confined to tropical and subtropical coastal communities, but with tropical fish now part of the global food supply chain, cases can occur anywhere. Victims typically experience a range of gastrointestinal and neurological symptoms with diagnosis based upon clinical presentation after recently eating a suspect fish species [4,5,6,7]. Paraesthesia and cold allodynia are diagnostic neurological symptoms for ciguatera across much of the Pacific basin [1,5,8].

Ciguatoxins activate voltage sensitive sodium channels at pM to nM concentrations and are the most potent, orally active, mammalian sodium channel toxins known [9,10]. They cause a hyperpolarizing shift in the voltage-dependence of channel activation resulting in channels opening at resting membrane potentials. The pathophysiological effects of the ciguatoxins are thought to be defined by their ability to cause the persistent activation of these channels and to also inhibit neuronal potassium channels, leading to increased neuronal excitability and neurotransmitter release, impaired synaptic vesicle recycling and modified Na^+^-dependent mechanisms in numerous cell types [11]. They share a common binding site (site 5) on sodium channels with the structurally similar polyether brevetoxins [12] and effect the cathepsin S-protease activated receptor-2 pathway that contributes to the sensory effects of the toxins [13].

Ciguatera is an uncommon but underreported disease in Australia with most cases caused by Spanish mackerel (*Scomberomorus commerson*) caught from the east coast of Australia or demersal reef fish from the Great Barrier Reef [5,6,14,15]. It is a notifiable disease in Queensland, Australia, but many cases go unreported, either because health care professionals are not familiar with the disease, or patients do not associate their illness with eating fish given the delayed onset of symptoms, which are often mild. There is no confirmatory test available to assist doctors with their diagnosis, and treatment is symptomatic and supportive only, although intravenous D-mannitol has shown promise as an early treatment for severe cases [16,17,18]. Between 2014 and 2020, there were 182 ciguatera cases reported to the health department for the State of Queensland, Australia (Queensland Health, notifiable conditions annual reporting: https://www.health.qld.gov.au/clinical-practice/guidelines-procedures/diseases-infection/surveillance/reports/notifiable/annual (accessed on 10 July 2019, 13 July 2020, 20 July 2021)). While there are sensitive and reliable laboratory methods for detecting and quantifying ciguatoxins from the flesh of fishes (reviewed by [19]), no rapid, reliable, cost-effective screening method is available to test commercial quantities of fishes prior to consumption.

**Figure 1:**
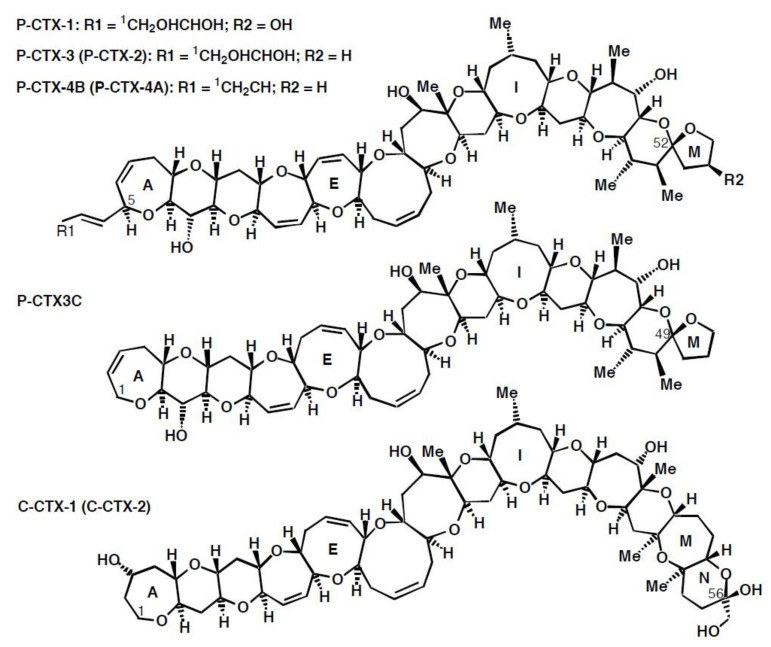
Pacific-ciguatoxin-1 (P-CTX-1), P-CTX-2 and P-CTX-3 (52-*epi*-P-CTX-2) are the major ciguatoxins found in ciguateric fishes from the east coast of Australia, with P-CTX-4A their presumed precursor produced by *Gambierdiscus* and *Fukuyoa* spp. P-CTX3C has been found in dinoflagellates and fishes from the Pacific Ocean, but not yet reported from Australia. Caribbean-ciguatoxin-1 (C-CTX-1) and C-CTX-2 are two of the major ciguatoxins found in fishes from the Caribbean Sea. P-CTX-2 and -3 differ by the stereochemistry of the H on C54, as does P-CTX-4A and -4B. C-CTX-1 and -2 differ by the stereochemistry of the CH_2_-OH sidechain on C56. P-CTX-1 is also known as CTX1B, P-CTX-2 as 52-epi-54-deoxyCTX1B, and P-CTX-3 as 54-deoxyCTX1B.

Ciguatoxin was the name given by Scheuer et al. [20] to the toxin in a partially purified extract from Pacific Ocean moray eels. Ciguatoxins are now considered to be a family of large, ladder-like, cyclic polyether toxins (Figure 1) with the structure of the first ciguatoxin isolated from fishes from the Pacific Ocean, Pacific-ciguatoxin-1 (P-CTX-1, also known as CTX-1B), determined by Murata et al. [21] using ciguatoxin that was isolated and purified in French Polynesia. The current nomenclature for the various toxin congeners is confusing and the subject of debate [22], with standard naming conventions often ignored. More than 20 analogs have been determined from fishes and the causative dinoflagellates from the Pacific with P-CTX-4A (Figure 1, previously designated as GTX-4A) being the presumed precursor of P-CTX-1 [22,23,24,25]. These analogs are generally referred to as belonging to either the P-CTX-4A or P-CTX3C family of toxins (Figure 1, reviewed by [22]).

P-CTX-1, or its 54-dexoy analog, P-CTX-2 (52-*epi*-54-deoxy P-CTX-1) (Figure 1), are the major toxins so far found in ciguateric fishes from the east coast of Australia and the Northern Territory [26,27,28,29]. A stereoisomer of P-CTX-2, named P-CTX-3 (52-*epi*-P-CTX-2), has also been extracted from ciguateric fishes [26,30,31,32] with P-CTX-3 having the lower energy configuration for the terminal furan M-ring attached to C-52 [33]. Lewis and Holmes [33] proposed a pathway where P-CTX-4A produced by dinoflagellates is bio-transformed through marine food chains to P-CTX-2/-3, and then to P-CTX-1. Evidence supporting this pathway was found from the Republic of Kiribati, where the flesh of carnivorous fishes generally had higher P-CTX-1 concentrations relative to P-CTX-2/-3, but P-CTX-2/-3 mostly dominated in the flesh of herbivorous and omnivorous fishes (relative to P-CTX-1) [32]. However, P-CTX-2 can sometimes be the dominant ciguatoxin in the flesh of carnivorous fishes in Australia and Kiribati [28,32], and Yogi et al. [31] found that P-CTX-3 was dominant in the flesh of a carnivorous blue-spot coral trout, *Plectropomus laevis* from Okinawa. Due to the different toxicities of the various ciguatoxin analogs, the toxicity of samples containing unknown or mixtures of toxins is often expressed in toxicity equivalents, especially as P-CTX-1 or P-CTX3C toxicity equivalents [22].

In addition to Pacific ciguatoxins, two other structural families of ciguatoxins are known based upon geographical location, the Caribbean ciguatoxins (C-CTX, Figure 1) and Indian Ocean ciguatoxins (I-CTX). Structures of four C-CTX analogs have been determined [34,35,36], along with additional toxic variants characterised by mass [34] but those from the Indian Ocean have to-date only been characterized by liquid chromatography-mass spectroscopy, with structure elucidation remaining elusive due to poor recovery during purification [37,38,39].

The origin of the ciguatoxins are microscopic, benthic dinoflagellate species belonging to the genera *Gambierdiscus* and *Fukuyoa*, normally found as epiphytes on macroalgae and a range of other benthic substrates [22,40,41]. They attach to substrates using mucous threads or “webs” [42] but can swim for short periods to be dispersed by local currents (tycoplanktonic). *Gambierdiscus toxicus* was the first microorganism linked to production of ciguatoxins by a French-Japanese collaboration working in the ciguatera-endemic Gambier Islands in French Polynesia [40,42,43]. To-date, 18 anterior-posteriorly flattened (discus-shaped) *Gambierdiscus* species, and 4 globular-shaped *Fukuyoa* species have been identified (Table S1). Most of the *Gambierdiscus* species and *F. ruetzleri* appear to be capable of producing ciguatoxins [22,44], although the cellular concentrations can vary greatly. The highest cellular concentrations of ciguatoxins appear to be produced by *G. polynesiensis* and *G. excentricus* [45,46,47]. It is now thought that the original description of *G. toxicus* [40] was based upon a sample containing more than one species, so Litaker et al. [48] redesignated the type species for *G. toxicus* based upon the GTT-91 isolate by Chinain et al. [49]. However, it is difficult to know how much of the literature published after 2009 referring to ciguatoxins from *G. toxicus*, is consistent with the revised description of Litaker et al. [48].

Most *Gambierdiscus* and possibly *Fukuyoa* species also produce more polar, water-soluble toxins with the first of these identified as maitotoxin (MTX). The name maitotoxin comes from the Tahitian word “Maito” for the lined-bristletooth surgeonfish (*Ctenochaetus striatus*) from which the putative toxin was first extracted [50]. The structure of the MTX extracted from a French Polynesian strain of *Gambierdiscus* was elucidated by Murata et al. [51] and renamed MTX-1 by Holmes et al. [52] to distinguish it from other maitotoxins. MTX-1 has a polyether structure unrelated to the ciguatoxins, and maitotoxins have not yet been shown to have a role in human poisoning. MTX-1 is one of the largest non-polymeric chemical structures ever determined and is the most potent marine toxin known when injected intraperitoneally into mice [51], but ciguatoxins are significantly more toxic when dosed orally to match the route of exposure in humans [53]. Two other maitotoxins, named MTX-2 and -3 were characterised from Queensland isolates of *Gambierdiscus* [52,54,55] and MTX-4 from *G. excentricus* [56].

The structure of MTX-3 was recently suggested to be 44-methylgambierone [57,58], and has been detected in many *Gambierdiscus* and *Fukuyoa* species [59,60,61,62,63,64]. However, Murray et al. [62] reported the toxicity (LD_50_) of 44-methylgambierone injected intraperitoneally (i.p.) into mice at between 20–38 mg/kg. The low toxicity and signs displayed by mice injected with 44-methylambierone [62] are inconsistent with the potency and signs induced by MTX-3 (Figures S1 and S2, Table S2). Indeed, while Holmes and Lewis [54] did not isolate sufficient MTX-3 to obtain a weight for pure material, a chromatographically enriched fraction containing MTX-3 had an LD_50_ < 50 µg/kg (Table S2), indicating ~1000-fold greater toxicity than 44-methylgambierone. Critical to its characterization, purified MTX-3 produced similar signs in mice (i.p.) to MTX-1 (LD_50_ ≈ 50 ng/kg, [51]) and MTX-2 (LD_50_ ≈ 80 ng/kg, [54]), all three toxins lost toxicity upon desulphation [54,65], could cause death in mice in <2 h when injected i.p. [52,54] Figure S1, and produced similar pharmacological responses in cardiac and smooth muscle tissues [54]. This characterisation shows that MTX-3 is not 44-methylgambierone.

Much of the early literature on the toxicity of *Gambierdiscus* from cultured isolates failed to use methods that could differentiate ciguatoxins from maitotoxins and therefore should be interpreted cautiously [22,23,66,67]. Early research on benthic dinoflagellates in Australia found only 2 of 13 laboratory clones of *Gambierdiscus* isolated from Queensland produced detectable concentrations of ciguatoxins by mouse bioassay [68].

Speculation on the dietary accumulation of toxins by fish to cause human poisoning has appeared in the literature from at least the 16th century. However, the marine food chain transfer of toxins causing ciguatera appears to have been first suggested by Mills [69] and later expanded by Randall [70] into a comprehensive hypothesis for the accumulation and bio-concentration of toxins from a benthic source into herbivorous fishes and then carnivorous fishes that prey on these herbivores. Lewis and Holmes [33] expanded on this model to incorporate ciguatoxin bio-transformations and dilution in fish through growth and toxin depuration. However, recent evidence suggests that ciguatoxins accumulate, but do not always bio-concentrate across trophic levels, at least in juvenile marine fishes [71,72] and freshwater goldfish [73]. The aim of this review is to develop conceptual models for the trophic transfers, accumulation, and loss of ciguatoxins in fishes for two different ecosystems that produce ciguateric fishes based upon our knowledge of ciguatera in Platypus Bay and the Great Barrier Reef on the east coast of Australia. We do this by modelling the transfer of ciguatoxins from their production in benthic dinoflagellates, to invertebrates/herbivores and then carnivorous fish for each ecosystem. Fisheries stock assessment and catch data are then used to model the dilution of ciguatoxins by growth in carnivorous fish species responsible for causing ciguatera from each ecosystem, Spanish mackerel (*Scomberomorus commerson*) from Platypus Bay, and common coral trout (*Plectropomus leopardus*) from the Great Barrier Reef. We also model the dilution of ciguatoxin from Spanish mackerel by a combination of somatic growth and depuration, and compare hypothetical depuration rates with the incidence of ciguatera from Spanish mackerel from the east coast of Australia. The relative risk of ciguatera from Spanish mackerel and coral trout is then compared. We use our review to identify knowledge gaps and make recommendations for future research directions.

## 2. A Conceptual Model for a Ciguateric Food-Chain in Platypus Bay

**Figure 2:**
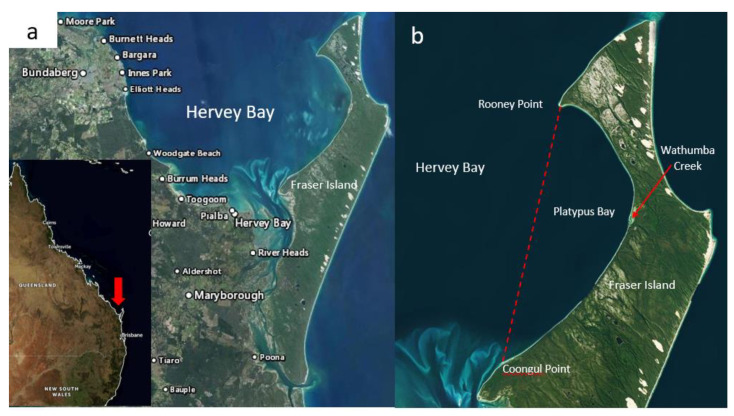
(**a**) Hervey Bay and Fraser Island, Queensland, Australia. Image adapted from QGlobe (https://qldglobe.information.qld.gov.au/ (accessed on 20 July 2021); Creative Commons Attribution 4.0 International (CC BY 4.0) licence). Inset: Outline of Queensland and New South Wales with arrow showing the location of Hervey Bay. Image adapted from Zoom Earth (https://zoom.earth/ (accessed on 20 July 2021)). (**b**) Platypus Bay is a smaller bay within Hervey Bay, and lies between Rooney Point and Coongul Point on Fraser Island. Image adapted from Zoom Earth (https://zoom.earth/ (accessed on 20 July 2021)).

Platypus Bay is a small (<400 km^2^), sheltered, sandy bay mostly <20 m in depth, which is bounded by a line between Rooney Point and Coongul Point on the north-west coast of Fraser Island (known as K’gari by the indigenous Butchulla people), Queensland (Figure 2). It forms the eastern boundary of the much larger Hervey Bay, and lies within the Great Sandy Marine Park. In recent times, it has become a destination for observing humpback whales as they transit on their annual southward migration from the Coral Sea to Antarctica. Two of the authors (RJL, MJH) began investigating Platypus Bay in the 1980′s because of the frequency of ciguatera from Spanish mackerel (*S. commerson*) and barracuda (*Sphyraena jello*) caught in its vicinity [5,74,75]. Platypus Bay is the only known location on the east coast of Australia where ciguateric fishes have been repeatedly caught [76,77], with many cases of ciguatera from Spanish mackerel reported across the Hervey Bay region in the late 1970’s and 1980’s [78,79]. However, because of Platypus Bay’s small area relative to that of the Great Barrier Reef (>340,000 km^2^), absence of a coral reef environment more typically associated with ciguatera, the fact that the fishes causing ciguatera are highly mobile pelagic species and the paradigm that fish retain toxicity [70], it was initially suspected that Spanish mackerel and barracuda might accumulate ciguatoxins elsewhere along the coast of Queensland [78,80]. It was thought that Platypus Bay was where toxic pelagic fish and mackerel fishers occasionally intersected, with fishers taking advantage of the shelter that Fraser Island provides from the predominate easterly winds. This was supported by the observation that extracts of livers from rabbitfish (*Siganus spinus*), a common herbivore that consumes the dominant macroalgae in the bay, were non-toxic and dredged macroalgal samples collected at 5 m depth contained no detectable *Gambierdiscus* [80].

However, later evidence pointed to Platypus Bay as the source of ciguatoxins contaminating Spanish mackerel. Firstly, in 1987 the Queensland Government banned the taking of Spanish mackerel and barracuda from Platypus Bay, after which, ciguatera cases in Queensland were no longer dominated by Spanish mackerel, but instead were mostly caused by demersal reef fish caught from the Great Barrier Reef [77,81]. Secondly, there was no obvious increase in frequency of ciguatera caused by demersal fishes from the Great Barrier Reef, but ciguatera cases from Spanish mackerel decreased. It is difficult to believe that prohibiting the taking of Spanish mackerel from such a small bay could make such a difference for a species capable of making annual migrations of more than a 1000 km [82]. It also raised questions about the accepted logic that fish retained toxicity for life, since toxic fish were not captured elsewhere given the high and previously unsustainable catches of this fish along the east coast of Queensland [83]. Thirdly, a family of recreational fishers were poisoned after eating blotched-javelin fish (*Pomadasys maculatus*) they caught in Platypus Bay [84]. The blotched-javelin is a small carnivorous grunter bream that is abundant in tropical estuaries and can grow to a length of 25 cm, but is not commercially important in Australia [85]. What was surprising about this ciguatera outbreak, was that all five family members were poisoned despite eating separate fish. In addition, when batches of the remaining 54 uneaten fish were assayed, every batch of fish was toxic [26]. While up to 100% toxicity of high-risk fish species is not unusual in ciguateric hot spots such as the islands of Tarawa and Marakei in the Republic of Kiribati [32,86,87], this remains a unique occurrence in Australia.

### 2.1. Food Chain Links Leading to Ciguatera in Platypus Bay, Trophic Level 1

In contrast to earlier studies using a dredge [80], hand collections of the benthic, free-living green filamentous macroalgae (*Cladophora* sp.) that covered large areas of the sandy bottom of Platypus Bay identified *Gambierdiscus* epiphytes at up to 556 cells per gram of wet weight *Cladophora* [77]. Observations of the benthos between 5–20 m depth using scuba diving found that the bottom of this ~50–100 mm thick macroalgal layer typically transitioned into blackened sand consistent with an anoxic layer at the interface between the sand and the overlaying blanket of macroalgae. Analysis of cultured clones of *Gambierdiscus* isolated from the *Cladophora* from Platypus Bay revealed that one of four produced detectable levels of ciguatoxins [68,88]. At the time, *Gambierdiscus* was a monotypic genus, so Holmes et al. [68] and Holmes and Lewis [88] attributed the species to *G. toxicus* and referred to the ciguatoxins they identified as gambiertoxins (GTX), following the naming convention originally proposed by Murata et al. [21] to distinguish these toxins from the ciguatoxins found in fish. Gambiertoxins were subsequently renamed as ciguatoxins by Satake et al. [23]. Based on ciguatoxin production, this suggests the presence of at least two species of *Gambierdiscus* in Platypus Bay, but we cannot exclude the possibility of multiple strains of one species with different toxin-producing capabilities. Holmes et al. [77] also assayed bio-detrital fractions sieved from bulk samples of *Cladophora* collected by scuba divers at ~15 m depth over a 22-month period. Extracts of the size fraction containing *Gambierdiscus* (45–250 µm), from one of these six bulk collections produced bioassay signs in mice consistent with ciguatoxins. While this sample had the highest *Gambierdiscus* populations [77], it presumably contained ciguatoxin-producing and non-, or low-ciguatoxin-producing species/strains, indicating that simple monitoring of *Gambierdiscus* populations alone may not accurately identify ciguatera risk even in ciguateric areas [22,89]. This conclusion is supported by studies at Flinders Reef in southern Queensland which had some of the highest population densities of *Gambierdiscus* so far found in Australia [90], but wild cells and cultured clones isolated from Flinders Reef did not produce detectable ciguatoxins [91], and demersal fishes, including herbivores, from this reef were also not toxic [92]. *Gambierdiscus* populations on Flinders Reef [90], in Platypus Bay [77], as well as in New South Wales [93] appeared to bloom seasonally, as observed elsewhere [94,95,96,97].

The cellular concentration of ciguatoxin in wild *Gambierdiscus* collected from Platypus Bay (as determined by mouse bioassay) was similar to those found in wild cells collected from Marakei Island in the Republic of Kiribati [68,77]. However, these concentrations were ~100-fold greater than those extracted from a cultured clonal isolate of *Gambierdiscus* collected from Platypus Bay [68], suggesting that ciguatoxin production in *Gambierdiscus* may be enhanced under natural conditions, or that higher ciguatoxin-producing species/strains (super-producers) remain to be isolated from Platypus Bay [77]. Elsewhere, there are few direct comparisons of cellular ciguatoxin production from wild and cultured populations of benthic dinoflagellates isolated from the same location. Attributing cellular toxin production to a wild sample is complicated by a potential mix of species/strains in the sample which may include cells having a range of toxin concentrations. Recently, Liefer et al. [97] found that *Gambierdiscus* populations in the U.S. Virgin Islands peaked in summer, but cellular ciguatoxin loads peaked in cooler months during lower population densities. Repeated sampling and toxin analysis of *Gambierdiscus* populations in Platypus Bay did not find evidence for such asynchronous cell toxin loads [77] but these studies need be repeated using the more sensitive toxin assays now available. Future studies in Platypus Bay could be facilitated by assays that allow the detection and species-identification of single *Gambierdiscus* and *Fukuyoa* cells [98]. It could also be interesting to grow cultured cells back in the wild to determine if ciguatoxin production of isolates match those from wild cells, possibly using mesocosm experiments or using dialysis chambers to grow cultured cells at a hot spot such as Platypus Bay. Alternatively, comparative intra- and inter-specific omics studies on ciguatoxin and non-ciguatoxin producing strains may provide new understandings of these differences [99,100]. Ciguatoxin concentrations in *Gambierdiscus* may also be altered by quorum-sensing bacteria [101].

The evidence indicates that some *Gambierdiscus* in Platypus Bay are a source of ciguatoxins [68,77] but that for much of the time, only low levels of ciguatoxins are produced that limit the contamination of fish in Platypus Bay with these toxins. However, a major gap in our knowledge is we do not know the species of *Gambierdiscus* living in Platypus Bay and the chemical profile of the toxins they produce. We only know that a cultured clone and possibly wild cells from Platypus Bay produced at least two ciguatoxins of different polarity [68,77,88]. Recent studies on the accumulation of ciguatoxins in fishes feeding on cultured *Gambierdiscus* used the TB-92 strain of *G. polynesiensis* isolated from French Polynesia [71,102]. The ciguatoxin profile of this strain is dominated by P-CTX3C and its isomers, with lesser amounts of P-CTX-4A [71,103], the precursor of the major ciguatoxin (P-CTX-1) found in ciguateric Platypus Bay fishes [26,33]. Ledreux et al. [71] suggested that the two toxin classes have different tissue depositions and retention in fishes, with P-CTX3C analogs possibly being poorly retained.

**Figure 3:**
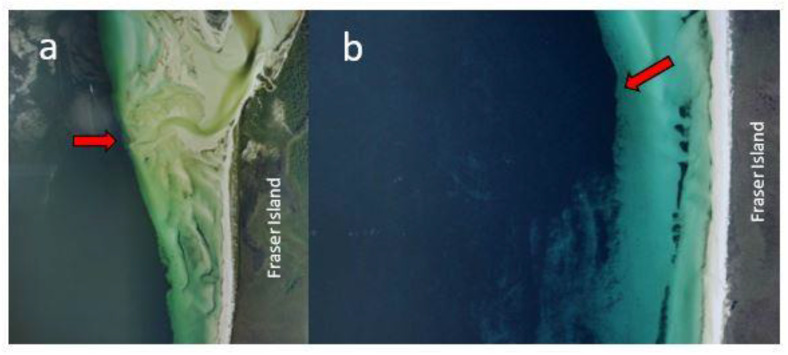
Benthic unattached macroalgae (*Cladophora*) lying on sand in Platypus Bay near the mouth of Wathumba Creek. Aerial photography from QImagery (Creative Commons Attribution 4.0 International (CC BY 4.0) licence): https://qimagery.information.qld.gov.au/ (accessed on 20 July 2021). (**a**) Arrow shows the eastern limit of a near-continuous, dark, benthic layer of *Cladophora* on 28 May 1994 at the entrance to Wathumba Creek. (**b**) Arrow shows limit of *Cladophora* near beach just south of Wathumba Creek on 24 June 2000.

We also do not know the spatial extent of the *Cladophora* layer and if it extends beyond Platypus Bay into Hervey Bay proper, or how much of this substrate that supports benthic dinoflagellates changes significantly through time. Archived aerial photography (QImagery: https://qimagery.information.qld.gov.au/ (accessed on 20 July 2021)) from 1994 and 2000 show that at times the *Cladophora* can be an almost continuous benthic layer in the eastern part of the bay (Figure 3), as it appeared to be during the studies by Holmes et al. [77] and Lewis et al. [84], whereas more recent satellite imagery (Queensland Globe: https://qldglobe.information.qld.gov.au/ (accessed on 20 July 2021)) suggests that the *Cladophora* layer near the beach can sometimes thin and become patchier (data not shown). The earliest available aerial photography images of Platypus Bay in QImagery are from 1958 and show dark streaks in the water consistent with *Cladophora*. Tidal currents form a large gyre in Platypus Bay [104] which may be a mechanism that helps maintain the unattached macroalgae within the bay. However, strong westerly winds can push considerable amounts of the macroalgae (presumably from shallower waters) up onto the western beach of Fraser Island, creating problems for vehicles driving on the beach that sink through a light covering of sand hiding a thick layer of rotting *Cladophora*. This wind-wave-driven transport of *Cladophora* onto the beach of Platypus Bay likely creates space for new *Cladophora* growth that would provide new substrate for epiphytic benthic dinoflagellates, especially in the shallower near-shore areas. It may also cause major, localized reductions in the benthic dinoflagellate populations in these shallow near-shore areas, reducing the risk of production of ciguatoxins within Platypus Bay. At other times, especially in deeper waters, a near continuous macroalgal substrate that can support *Gambierdiscus* populations likely covers many km^2^.

Much of Platypus Bay has no reef structure, so in the past, fishers would dump objects such as old washing machines at sites within the bay to try and create secret artificial reefs. One such location is about 1.5 nautical miles west of Wathumba Creek (Figure 2) where benthic dinoflagellates and fish were collected [77]. While the site consistently held a range of fishes such as trevallies (*Caranx* spp.) and yellowtail kingfish (*Seriola lalandi*), assays of liver extracts from fish caught at this site were nontoxic (RJL unpublished results), so we have no evidence that these sites had any role in exacerbating the production of ciguateric fishes in Platypus Bay.

Hervey Bay supports the largest seagrass meadows (>2000 km^2^) on the east coast of Australia [105] and subtidal mapping by the Queensland Government suggest that some deep- and shallow-water seagrass beds cover the south-western part of Platypus Bay (Wetland*Maps*: https://wetlandinfo.des.qld.gov.au/wetlandmaps/ (accessed on 20 July 2021)). There have been some studies of the epiphytes on Hervey Bay seagrasses but none that report any benthic dinoflagellate populations. Hervey Bay seagrasses support one of the largest populations of dugong (*Dugong dugon*) on the east coast of Australia [106], but as yet there are no reports of illness or death of these marine mammals linked to possible poisoning by feeding on seagrasses with epiphytic benthic dinoflagellates. *Gambierdiscus* has been found on seagrass (*Zostera*) on the east coast of Australia [93,107], and the deaths of manatees [108] and dolphins [109] have been linked in the U.S.A. to poisoning by brevetoxins produced by the dinoflagellate *Karenia brevis*. Brevetoxins have a similar mechanism of action as ciguatoxins and compete for the same receptor binding site on Na^+^-channels [12], suggesting that dugongs may also be sensitive to ciguatoxins. This hypothesis is supported by ciguatoxins being detected from the tissues of living and dead Hawaiian Monk seals [110]. However, there is no evidence currently for the poisoning of dugong from feeding on these seagrasses, so we think they are unlikely to be a major source of ciguatoxins into local marine food chains.

An examination of the origin of nutrients that support growth of *Cladophora* and the associated benthic dinoflagellates in Platypus Bay suggest that there are likely three major sources. Firstly, recycling of nutrients in situ from dead and decaying biomass of *Cladophora* and the invertebrates living within this macroalgal layer (including gastropods, crustaceans, polychaetes and nematodes; [84]). Secondly, episodic upwelling where summer north-west winds can drive wind- or Ekman-driven coastal and shelf-edge upwelling to replace the surface waters transported offshore by the wind, with intrusions of upwelling waters flowing into northeast Hervey Bay [111,112]. Thirdly, catchment derived nutrients transported offshore in flood plumes that can extend well out into Hervey Bay. For example, in 1992 sediment transport from two major floods and a cyclone in a 3-week period caused the loss of more than 1000 km^2^ of seagrass in Hervey Bay [113]. However, outside of flood events, there is little freshwater flow into the bay [104]. Even though Hervey Bay is not within the Great Barrier Reef Marine Park, the Burnett-Mary region is adjacent to Hervey Bay and is the southernmost Natural Resource Management (NRM) region for the Great Barrier Reef. Many Great Barrier Reef ecosystems continue to be in poor condition due largely to the collective impact of land run-off associated with past and ongoing catchment development, coastal development activities, extreme weather events and climate change impacts [114]. To reduce these impacts, targets have been developed from a 2013 baseline for the reduction by 2025 of end-of-catchment loads of nutrients and sediments, and concentrations of pesticides (mostly herbicides) that flow from the major river catchments into the Great Barrier Reef lagoon ([115], Great Barrier Reef 2050 Water Quality Improvement Plan, https://www.reefplan.qld.gov.au/ (accessed on 20 July 2021)). This includes targets for the largest three rivers that discharge into Hervey Bay, the Mary, Burrum and Burnett rivers (Figure S3). The largest load reduction targets are for the Mary River, the flood plume of which is the most likely to reach Platypus Bay [116]. If the Burnett-Mary region load reduction targets are met, it could reduce catchment-derived nutrients reaching Platypus Bay and its benthic layer of *Cladophora* and epiphytic dinoflagellates. If this reduction in nutrients led to reduced populations of ciguatoxic *Gambierdiscus*, it would be an unintended, but direct benefit from the 2050 Great Barrier Reef Water Quality Improvement Plan. However, a study at reef sites in the US Virgin Islands found that enriched nutrient levels do not always produce significantly greater populations of *Gambierdiscus* [117].

Fraser Island is the largest of the five large barrier sand islands along the southern coast of Queensland and may also be a local source of nutrients to Platypus Bay. Wathumba Creek is one the largest of the western flowing creeks on Fraser Island and it flows into the middle of Platypus Bay (Figure 2). However, these barrier sand islands tend to be low in nutrients with oligotrophic lakes and streams fed by underground sand aquifers. The majority of Fraser Island is also World Heritage listed National Park and the only permanent human structure along the coast of Platypus Bay from which anthropogenic nutrients could be exported is a camping toilet block near to Wathumba Creek. It is also possible that nutrients are occasionally input from aeolian sources such as smoke and ash from infrequent but large bushfires that can persist in much of the inaccessible wilderness of Fraser Island, such as the recent fire that burned for 6 weeks on Fraser Island in late 2020.

A pesticide reduction target has been set under the Great Barrier Reef 2050 Water Quality Improvement Plan based upon keeping concentrations below that required to protect at least 99% of aquatic species from all pesticides at river mouths ([115], Great Barrier Reef 2050 Water Quality Improvement Plan, https://www.reefplan.qld.gov.au/ (accessed on 20 July 2021)). Pesticide reduction is not a priority for catchments flowing into Hervey Bay; however, at moderate flows of the Mary River, nearshore seagrass areas are at risk of being exposed to concentrations of herbicides that are known to inhibit photosynthesis [118]. For example, during a low flow period in the Mary River, the photosystem-II inhibitor diuron was detected near the edge of Platypus Bay at 5 ng/L [118]. However, herbicide concentrations tend to be highest during “first-flush” events during high river flows, not during periods of low river flows [119]. Community composition changes in benthic microalgae communities can occur at low µg/L concentrations of diuron [120,121]. Currently there is insufficient information to know if catchment derived herbicide concentrations could be having an impact on populations of *Gambierdiscus* in Platypus Bay.

Several species of benthic dinoflagellate other than *Gambierdiscus* also occur as epiphytes on the *Cladophora* in Platypus Bay, including *Prorocentrum* sp. and *Coolia* sp. [77], but these have not yet received much study in Platypus Bay. Many benthic dinoflagellate species produce toxins but apart from *Gambierdiscus* and *Fukuyoa*, there is limited evidence for their involvement in the ciguatera food chain [33,67,77]. Holmes et al. [122] did isolate a new toxin from a clonal culture of *Coolia* collected from the *Cladophora* that was distinct from ciguatoxins and named it cooliatoxin. At the time, *Coolia* was a monotypic genus, so Holmes et al. [122] attributed the species to *C. monotis*. A subsequent review based mostly upon old scanning electron micrographs suggested that the species may have been *C. tropicalis* [123]. 44-methylgambierone has recently been detected from *C. tropicalis* and *C. malayensis* [61,62,63,124] along with an additional isomer from *C. tropicalis* [124]; however, the greater mass and toxicity of cooliatoxin show that it is distinct from 44-methylgambierone [63,122]. *Coolia tropicalis*, *C. malayensis*, *C. canariensis* and *C. palmyrensis* are now known to occur in Queensland waters [125,126].

### 2.2. Food Chain Links Leading to Ciguatera in Platypus Bay, Trophic Level 2

The most obvious herbivorous fish in Platypus Bay are rabbitfish (*Siganus spinus*) [80,84] that rarely exceed 24 cm in length (Fishes of Australia: http://fishesofaustralia.net.au/home/species/1886 (accessed on 20 July 2021)). Rabbitfishes are known to accumulate ciguatoxins in the Pacific Ocean [32,87], with the highest concentration recorded being 0.13 µg/kg P-CTX-1 equivalents from the Republic of Kiribati [87]. Rabbitfishes are grazers that feed on filamentous algae or browse on larger macroalgae and are abundant around tropical reefs [127]. They are also abundant in Platypus Bay with stomachs often tightly packed full of *Cladophora* [80,84]. Even though these rabbitfishes presumably consume significant numbers of *Gambierdiscus*, to our knowledge they rarely cause ciguatera [84] and extraction and analysis of the dissected livers from 50 rabbitfish caught from Platypus Bay failed to detect any ciguatoxins [80]. While rabbitfishes are generally not targeted by recreational fishers in Queensland, small quantities are netted from Platypus Bay and sold by commercial fishers (Table 1). The consumption of rabbitfishes from Platypus Bay, apparently without harm, is not consistent with the classical food chain hypothesis for the bioaccumulation and bio-concentration of ciguatoxins [70], unless the toxins are not efficiently absorbed by rabbitfishes, and/or any toxins bio-accumulated are rapidly depurated. Assuming an average weight of ~0.5 kg per rabbitfish, the total commercial catch of more than 45 tonnes for the 25 years between 1994 and 2018 (Table 1) equates to the sale and presumed human consumption of >90,000 fish over this time frame, apparently without harm. Similarly, in the ciguatera endemic Cook Islands, *S. spinus* is a targeted food fish that rarely causes ciguatera [128].

Platypus Bay appears to be a case where the classical transfer of ciguatoxins from benthic dinoflagellates to herbivorous fish may not apply. Kelly et al. [129] proposed a model for an expanded marine food chain for ciguatera that included invertebrates in the bio-transfer of ciguatoxins. Lewis et al. [84] found evidence for this transfer of ciguatoxins through invertebrates in Platypus Bay (especially Alpheid shrimps), to small carnivorous fish (blotched-javelin fish) that feed on invertebrates in the *Cladophora*. Based on this finding, they suggested that Spanish mackerel might accumulate ciguatoxins by preying on these blotched-javelin fish. Even though ciguatera is generally considered a disease caused by fishes, ciguatoxins have also been detected in molluscs, crustaceans, and echinoderms, reinforcing the evidence for the potential transfer of these toxins through invertebrates [32,130,131,132,133,134].

Cells of cultured *Gambierdiscus* were lethal when fed to laboratory reared brine shrimp [129,135]. The brine shrimp may have been intoxicated by ciguatoxins and/or water-soluble toxins, as the latter are produced by most *Gambierdiscus* species/strains, unlike the ciguatoxins which are produced in detectable quantities by fewer species/strains [46,53,68]. Behavioural changes in invertebrates caused by intoxication from feeding on benthic dinoflagellates could increase their probability of being preyed upon [33,135], and invertebrates feeding upon ciguatoxin producing dinoflagellates could be a mechanism that concentrates ciguatoxins into predatory fish [84]. It appears that this concentration step in the food chain is required for ciguatoxins to accumulate in higher trophic level to cause ciguatera from Platypus Bay fishes.

### 2.3. Food Chain Links Leading to Ciguatera in Platypus Bay, Trophic Levels 3 and 4

Spanish mackerel, barracuda and blotched-javelin are three carnivorous fish species caught from Platypus Bay known to cause ciguatera [74,75,78,84]. The ciguatoxic blotched-javelin and barracuda caught from Platypus Bay were relatively small fish, <0.5 and ~2.0 kg, respectively [78,84], whereas toxic Spanish mackerel tend to be much larger (typically >10 kg). Lewis et al. [84] suggested that barracuda and Spanish mackerel acquire ciguatoxins from preying on blotched-javelin fish, with Spanish mackerel also possibly preying on barracuda. Blotched-javelin can be considerably more toxic than Spanish mackerel, and both fish species carry the same suite of major ciguatoxins [26]. These results are consistent with the prey fish species (blotched-javelin) frequently feeding within an area producing ciguatoxins, and sometimes being more toxic relative to its predator because the larger Spanish mackerel are only transiently feeding within the toxic area on a mix of toxic and non-toxic prey fishes. This reduction of toxin concentration up the food chain is also consistent with ciguatera in French Polynesia where the lower trophic level fishes (herbivores) tend to be more toxic than the higher trophic level piscivorous fish that feed upon them [136,137]. However, this is contrary to the conceptual model for the bio-concentration of toxins along the ciguatera food chain from herbivorous to carnivorous fishes, and from small to large carnivorous fishes [70]. The highest known ciguatoxin concentrations from the Pacific Ocean are found in predatory fishes, and in ciguatera endemic regions such as the Republic of Kiribati, the pattern of toxicity in fish appears to be consistent with toxin concentration along the food chain [32,87].

Some fish are susceptible to intoxication by ciguatoxins [71,73,138,139,140]. If blotched-javelin are affected by ciguatoxins, then they may be more vulnerable to predation making this a mechanism by which blotched-javelin contaminated with ciguatoxins are selectively preyed upon by larger predators such as Spanish mackerel [84]. This increases the potential for transfer of ciguatoxins along the food chain into larger fish that are more likely to enter the human food chain in Australia. However, blotched-javelin fish do not always carry detectable levels of ciguatoxins in Platypus Bay, as Lewis et al. [84] caught non-toxic fish <1 year after finding toxic fish.

Overall, these results are consistent with significant levels of ciguatoxins only intermittently entering the marine food chain in Platypus Bay because:

- The spatial extent of the *Cladophora* layer varies over time, limiting the available substrate for benthic dinoflagellates;
- Most of the *Gambierdiscus* species/strains present are not those that produce significant levels of ciguatoxins [68,77];
- And/or, the population of ciguatoxin-producers are sometimes too low for significant amounts of toxin to enter food chains in the bay [77];
- And/or, the profile of the ciguatoxins being produced varies over time, with possibly P-CTX3C congeners less likely to be retained by fishes [71];
- And/or, ciguatoxins are produced, but are not concentrated/transferred into the invertebrate populations being eaten by small predatory fish; and
- And/or, ciguatoxins are not accumulated by small predatory fish (e.g., blotched-javelin) at high enough concentrations to increase their risk of predation.

Any of the above factors would act as a rate-limiting step to the input and transfer of ciguatoxins along food chains in Platypus Bay and explain the observation by Lewis [78] that ciguatera cases from Spanish mackerel cycle through periods of high and low incidences.

After the prohibition on the taking of Spanish mackerel and barracuda from Platypus Bay was scheduled into law in 1987, most ciguatera cases in Queensland were caused by demersal reef fish caught from the Great Barrier Reef [76,77,81]. However, even after this ban, recreational and commercial fishers continued to take Spanish mackerel from non-prohibited waters beyond Platypus Bay, with commercial fishers’ logbooks showing Spanish mackerel continue to be taken from within the two 30 × 30 nautical mile logbook grids/squares (W32, W33) that include Platypus Bay (Figure S4), presumably from waters to the north and east of Fraser Island. Between 1990 and 2019, commercial fishers caught an average of 11.9 ± 4.8 tonnes annually (mean ± standard deviation) of Spanish mackerel from within the logbook grids that include Platypus Bay (W32 and W33; range 3.5 to 24.3 tonnes; QFish [141]). In addition, an average of 2.5 ± 1.4 tonnes of Spanish mackerel (range < 1–5.6 tonnes) were caught annually between 1990 and 2019 from Hervey Bay in the two logbook grids to the west of Platypus Bay (V32, V33, Figure S4). The combined catch from these four grids (V32, V33, W32, W33) that encompass Platypus Bay and its adjacent waters, showed considerable annual variation ranging from a low of 4 tonnes in 1994 to 26 tonnes in 1999 (Figure S5). Assuming an average weight of 7.7 kg for commercially caught Spanish mackerel [83], this equates to between ~500 and 3300 fish caught annually between 1990 and 2019 from waters surrounding Platypus Bay, apparently without causing the regional levels of ciguatera reported previously by Lewis [78].

Historically, most cases of ciguatera along the east coast of Australia have been caused by demersal reef fishes caught from along the Great Barrier Reef north of about Mackay (~21° S latitude), and by Spanish mackerel caught south of this latitude [6,74,76], including in recent years from New South Wales [14]. This north-south divide is not absolute, with Spanish mackerel from north Queensland occasionally causing ciguatera [15,79] and demersal reef species more usually associated with the Great Barrier Reef sometimes causing ciguatera in southern waters, e.g., a mild case of ciguatera was caused in 2016 by a blue-spot coral trout (*Plectropomus laevis*), caught off Moreton Island (~27° S) (MJH personal communication). The ciguatera suffered by people eating toxic Spanish mackerel has ranged from very mild cases through to critical poisonings requiring hospitalization, occasionally leading to death [5,78,142].

Spanish mackerel from the east coast of Australia form a single genetic stock in coastal shelf waters between Cape York Peninsula and northern New South Wales, but some travel long distances within this range with the longest recorded from tagging studies being up to 1000 nautical miles from northern Queensland to New South Wales [79]. Their movement patterns depend on spawning and feeding behaviours, water temperatures and currents [79]. Fishers often interpret Spanish mackerel movement as a southward’s coastal migration of fish from Queensland waters to northern and central New South Wales waters in summer, and then a northern return to Queensland waters in late autumn. Movement is likely more complex than this but may in part relate to fish attaining favourable environmental or feeding conditions within a 24 °C isotherm [79]. There is a correlation between the distance moved southwards and fish size, with the larger sized fish usually being females [79].

Before the prohibition on taking Spanish mackerel from Platypus Bay, ciguatoxic Spanish mackerel were caught in southern Queensland during most months with possibly a gradual increase through winter and early spring to a maximum in October ([78], Figure S6). This was for fish caught between Maryborough and Gladstone (i.e., the waters of Hervey Bay and coastal waters to the north of Hervey Bay) between 1976 and 1983. Spanish mackerel from the east coast of Australia show predictable, seasonal migratory behaviour with annual spawning concentrated over a two lunar month period between September and November near a spatially discrete group of inner reefs northeast of Townsville (Figure S7) on the central Great Barrier Reef [143]. It is one of the most predictable spawning aggregations of fish on the Great Barrier Reef [83], but this does not mean there is a salmonid-like return to the same spawning reefs [79]. Most of Queensland’s annual commercial catch of Spanish mackerel is taken each year in spring between September and November from these central Great Barrier reefs [144]. In contrast, most of the Spanish mackerel caught by commercial fishers from the waters around Platypus Bay are taken between April and August (Figure S6). The predominance of ciguatoxic Spanish mackerel caught between April and October [78] likely reflects when most fish are being caught in this region, either of resident fishes before they move north for spawning, or as part of the general northwards’ movement of fish along the east coast of New South Wales and Queensland from late summer and autumn. However, spawning may also occur on isolated reefs as far south as Hervey Bay and fishers in this region believe that some fish do not always leave the area [79].

If all Spanish mackerel, including those feeding in Platypus Bay, showed high fidelity to the spawning aggregations northeast of Townsville, then we might have expected a spike in ciguatera cases from Spanish mackerel caught from the spring spawning aggregations, especially given the very high exploitation rates in this fishery [79], but this has not been noticeable. However, a recent analysis did find a slightly increased frequency of ciguatera associated with mackerel species (*Scomberomrus* spp.) between November-March, corresponding to the Austral wet season from late spring to early autumn [15]. East coast Queensland stocks of Spanish mackerel were previously considered either fully-fished or overfished relative to maximum sustainable levels, initially reaching annual landings of around 1000 tonnes during the 1970’s [143]. The east coast stock is now thought to be sustainably fished, but maximally exploited [83]. However, the recent increase in ciguatera cases from Spanish mackerel caught in New South Wales [14], might be consistent with fish feeding in Platypus Bay and becoming contaminated with ciguatoxins before moving south along the east coast of Australia into New South Wales. The distribution of Spanish mackerel along the east coast of Australia has been found to be especially sensitive to the environmental effects of climate change with southward range shifts into southern New South Wales exceeding 200 km per decade [145].

A range of large carnivorous fishes other than Spanish mackerel are often captured from Platypus Bay and apparently eaten without harm by recreational fishers. We know this from personal observations (MJH, RJL) and regional reports published in recreational fishing publications from charter boat operators and fishing guides. The carnivorous fish species caught by recreational and charter fishers from Platypus Bay include at least five species of trevally (*Caranx* spp.), yellowtail kingfish (*Seriola lalandi*), spotted mackerel (*Scomberomorus munroi*), school mackerel (*S. queenslandicus*), mackerel-tuna (*Euthynnus affinis*), longtail tuna (*Thunnus tonggol*) and juvenile black marlin (*Makaira indica*). Spotted mackerel, school mackerel, trevally and yellowtail kingfish have caused ciguatera in Australia, but not many cases are known [1,5] and we are not aware of any cases of ciguatera in Australia from longtail tuna, marlin or mackerel-tuna (mackerel-tuna are not often caught for food in Australia and black marlin are legally protected from commercial fishing in the Australian Fishing Zone). Possibly, these large predatory species do not often feed within the ciguatera-food chain in Platypus Bay, with some instead preying upon small pelagic, plankton-feeding fishes such as scads (Carangids) and herrings (Clupeids). Indian scad (*Decapterus russelli*) are common along the east-coast of Queensland, including Platypus Bay and may be one such small prey species. Lewis [80] assayed Indian scad captured from Platypus Bay at the same time as toxic barracuda were captured but could not detect any ciguatoxin. It is therefore likely that there are a range of non-toxic food chains in Platypus Bay, into which many predatory fishes feed. Stable isotope analysis of fishes from the Great Barrier Reef suggests that there is little dietary niche overlap of Spanish mackerel with predatory demersal reef fishes [146]. If dietary overlap is the exception, as suggested by Espinoza et al. [146], and if it also operates outside of Great Barrier Reef waters, then separate, non-toxic food chains may be a mechanism that prevents ciguatoxins from benthic dinoflagellates contaminating most predatory fishes in Platypus Bay. The availability of toxic and non-toxic food chains to a predatory fish species in highly ciguatoxic areas may exert selection pressure against ciguateric fish populations if the behaviour or health of these fish are affected by the ciguatoxins.

There are also several fish species caught from Platypus Bay that appear to be inconsistent with our hypothesis for the food chain production of ciguateric fishes in Platypus Bay. For example, snapper (*Chrysophrys auratus*) is a large, long-lived (>30 years) predatory Sparid species often caught by recreational fishers from Platypus Bay (reports from recreational fishing magazines and weekly fishing reports for Hervey Bay). This prized sporting and table fish typically does not move large distances [147] with the species so heavily exploited in southern Queensland that stocks are classified as overfished past maximum sustainable yield [147]. Inshore sheltered habitats such as Hervey Bay provide important nursery grounds for juvenile snapper, which feed mainly on worms, crustaceans, and other invertebrates, while adults have a wider diet including small fishes and hard-shelled invertebrates that they crush with their molar-like teeth [147]. Surprisingly, we are not aware of any cases of ciguatera caused by snapper, despite their feeding preference overlapping those of blotched-javelin fish. Given the targeting of this species by fishers, and their high level of exploitation, it is likely that snapper are rarely ciguateric.

A range of small, carnivorous fish species normally associated with shallow water estuaries and open beaches are also caught by recreational fishers from along the shoreline of Platypus Bay, including whiting (*Sillago* spp.), flathead (*Platycephalus* spp.) and yellowfin bream (*Acanthopagrus australis*). Despite these species eating a range of benthic invertebrates and small fishes [148], to our knowledge they have never caused ciguatera. In the past, these species were also netted from the beach of Platypus Bay and sold by commercial fishers (RJL personal communication). Possibly these species do not feed within the *Cladophora*, otherwise it is difficult to understand why they would not be exposed to ciguatoxins in their diet in Platypus Bay.

### 2.4. Summarizing the Production and Food Chain Transfer of Ciguatoxins in Platypus Bay

**Figure 4:**
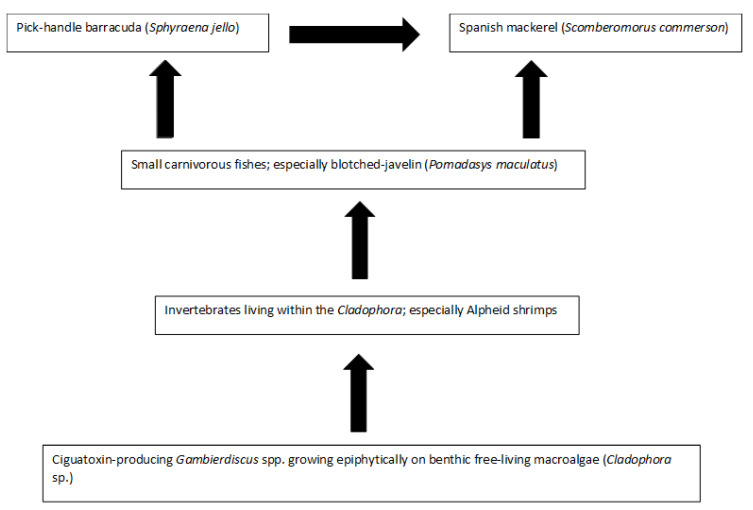
Conceptual model for the food chain transfer (arrows) of ciguatoxins through at least three trophic transfers in Platypus Bay, Fraser Island.

Platypus Bay appears to be a unique ecosystem for production, transfer, and accumulation of ciguatoxins into higher trophic level fishes. It is the only location known that repeatedly produces ciguateric fishes along the east coast of Australia. Ciguatoxins are produced by *Gambierdiscus* spp. growing epiphytically on unattached macroalgae (*Cladophora* sp.) lying over an unconsolidated sandy substrate. We suggest these benthic dinoflagellates are consumed by invertebrates living within the macroalgae, principally Alpheid shrimps, which are in turn preyed upon by blotched-javelin fish (*Pomadasys maculatus*). The blotched-javelin are eaten by transient pelagic carnivores, especially Spanish mackerel (*Scomberomorus commerson*) (Figure 4). If these Spanish mackerel are caught and eaten before they can depurate ciguatoxins, ciguatera poisoning occurs. However, further research is required to determine the chemical profile of the ciguatoxins produced by Platypus Bay *Gambierdiscus* and the bio-transformations that occur through at least three trophic transfers.

Platypus Bay is a simpler ecosystem compared to the diversity and complexity generally found on coral reefs, and this relative simplicity may facilitate future studies on ciguatera food chains, which should include:

- Identifying the resident *Gambierdiscus*/*Fukuyoa* species in Platypus Bay;
- Determining the profile and quantities of ciguatoxins produced by these species;
- Identifying the profile and quantities of ciguatoxins that bio-transfer across Platypus Bay trophic levels; and
- Determining the relationship between the spatial extent of the *Cladophora* substrate and the risk of ciguatera to allow the development of remote sensing imagery as a monitoring tool (e.g., [149]).

## 3. Model for the Dilution of Ciguatoxins in the Flesh of Spanish Mackerel (*S. commerson*) through Growth

Lewis and Holmes [33] developed a model for the factors that influence the accumulation of ciguatoxins through marine food chains, hypothesising that somatic growth could dilute the concentrations of ciguatoxins contaminating the flesh of fishes over time. Similar suggestions were also made by Yang et al. [150]. However, the suggestion that fish could lose toxicity was made as early as Halstead and Bunker [151] who suggested the toxin could metabolise in the liver of fishes and that higher concentrations of toxin could be expected in the liver and intestine, and lower concentrations in the muscle if the fish had been captured soon after feeding on the source material. Randall [70] did not agree with this hypothesis. However, Banner et al. [152] also suggested that fish could lose toxicity, in part to account for some detailed observations by Cooper [153] of localized changes in fish toxicity on reefs in the Gilbert Islands (Republic of Kiribati). Clausing et al. [102] recently provided experimental support for the concept of somatic growth diluting ciguatoxin concentrations in juvenile herbivorous unicornfish (*Naso brevirostris*) fed on a gel diet containing a cultured clone of *G. polynesiensis* that produces a ciguatoxin profile dominated by P-CTX3C.

We therefore constructed growth-based models to explore the potential for dilution of ciguatoxin concentrations in the flesh of commercial (legal) sized Spanish mackerel to below that which could cause human poisoning, i.e., below the precautionary action concentration of 0.01 µg/kg of P-CTX-1 equivalents suggested by the U.S. Food and Drug Administration (FDA) for the safe consumption of seafood [154]. We only consider ciguatoxin concentration in the muscle (flesh) of fishes, because in Australia the potentially more toxic viscera (including gonads) of Spanish mackerel and reef fish are generally discarded and not sold commercially. Our first model is based only upon dilution of toxicity through somatic growth and assumes that Spanish mackerel do not depurate or metabolize ciguatoxins from their tissues. This is the simplest model and was a mechanism proposed by Lewis et al. [86] for reducing fish toxicity over time. The model assumes that if there is no loss or gain of toxin over time, then the ciguatoxin concentration in muscle will decrease in proportion to the relative increase in mass from somatic growth. That is, if [CTX]*_i_* is the concentration of ciguatoxin in year *i* in µg/kg, then the concentration in year *i* + 1 can be described by:

[CTX]*_i_*_+1_ = [CTX]*_i_* × mass*_i_*/mass*_i_*_+1_ (1)

Spanish mackerel are the largest of the mackerel species found in Australian waters and are targeted by both commercial and recreational fishers because of their large size, good eating qualities and because they are fun to catch. Spanish mackerel can reportedly grow to 2.4 m in length and weigh up to 70 kg [82], although fish of this size are rarely caught now. One of the largest fish captured in recent years weighed 54 kg and was caught off Fraser Island in 2015. Fisheries scientists estimated its age at 26 years (Queensland Department of Agriculture and Fisheries, personal communication). Spanish mackerel are a fast-growing fish, especially in the first year, with females reaching sexual maturity at ~89 cm total length between 2- and 4-years of age [83,144]. Males and females have different growth rates with females growing faster and larger [82]. Both Queensland and New South Wales have the same minimum legal size of 75 cm (total length) for the taking of Spanish mackerel by commercial and recreational fishers. This means fish become vulnerable to harvesting along the east coast of Australia mostly between 1- to 2-years of age with the estimated age at which fish become 50% vulnerable to fishing being ~1.5 years, and 95% vulnerable by ~2.1 years [83].

**Figure 5:**
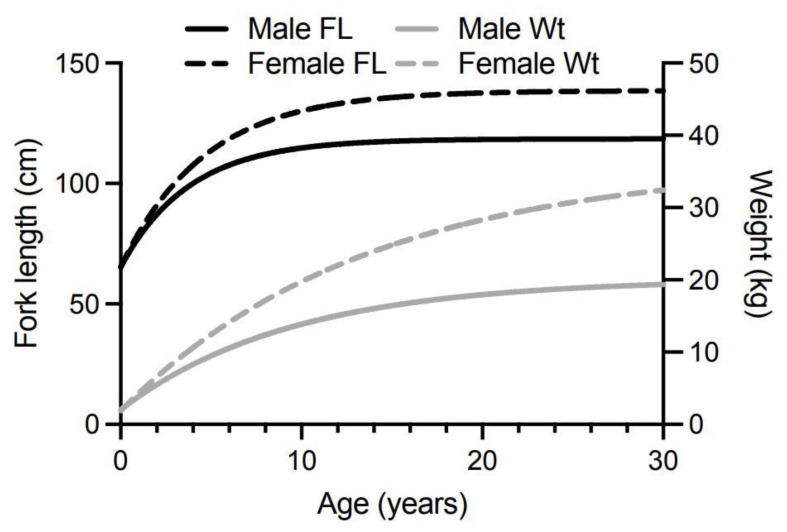
Growth curves for Spanish mackerel from the east coast of Australia (data supplied by Michael O’Neill from O’Neill et al., [83]). The black lines show von Bertalanffy growth curves for fork length-at-age for male and female Spanish mackerel. The grey lines show weight-at-age for growth of male and female Spanish mackerel.

We constructed our model from von Bertalanffy growth curves for male and female Spanish mackerel (Figure 5) based upon fish fork lengths at age [83]. The von Bertalanffy growth curve is one of the most widely used models for describing fish growth in fishery science, although it often performs poorly for young ages for which there are usually little data. Often, the observed data shows a broad distribution around the modelled growth curve because of considerable variation in the lengths that individual fish can reach for the same age [155]. O’Neill et al. [83] reported most Spanish mackerel caught by commercial and recreational fishers belong to annual cohorts up to 7-years of age. We therefore modelled changes in ciguatoxin concentrations in fish between 0.5- to 10-years of age to ensure we cover most of the fish sizes caught and eaten from the east coast of Australia. While fast-growing fish may reach the minimum-legal-size for harvesting before 1-year of age, most only reach this total length between 1–2 years of age [83]. The minimum-legal-size of 75 cm total length corresponds to about 67 cm fork length (fisheries scientists more commonly use fork length than total length as the measure for fish length, but legal limits for harvesting fish are based upon total length). Total length for Spanish mackerel is related to fork length by the equation of Begg et al. [156]:

Total length (cm) = 4.275 + (1.06 Fork length (cm)) (2)

Our models rely upon conversion of Spanish mackerel fork length-at-age values, to weight-at-age values (Figure 5), using the equation of Campbell et al. [157]:

Weight (kg) = 2.35 × 10^−6^ (Fork Length (cm)^3.2766^) (3)

The consistent increase in weight-for-age that this mathematical function produces’ does not reflect the reality of seasonal changes in weight that animals in the wild undergo, especially in relation to gonad development and spawning. Prior to spawning, the gonads can contribute a significant amount to the total weight of the fish with ovaries of female Spanish mackerel ranging from <0.1% to 13% of fish body weight, and male testes ranging between <0.1% to 7% of fish body weight [156].

Our growth dilution model assumes the concentration of ciguatoxins are homogeneous throughout the flesh of fish, and this is supported by recent findings that P-CTX-1, -2 and -3 were similarly distributed throughout the fillets of yellow-edge coronation trout (*Variola louti*) caught from Okinawa [158]. However, it is possible that the concentrations of ciguatoxins across the tissues of Spanish mackerel fillets could vary, as Li et al. [72] have recently shown that much higher concentrations of ciguatoxins can accumulate in the skin compared to the muscle in goldspotted rockcod (*Epinephelus coioides*). In Queensland, Spanish mackerel fillets or “steaks” are normally sold with the skin still attached, whereas demersal reef fish fillets are normally sold skinned (Queensland Department of Agriculture and Fisheries, personal communication). Previous studies in the Caribbean found that toxicity was evenly distributed throughout the flesh of a ciguatoxic trevally (*Caranx bartholomaei*) [159]. In the Pacific, Helfrich et al. [160] reported that there was no difference in toxicity of the flesh of red bass (*Lutjanus bohar*) tested from different parts of the body, and Banner [161] found no statistical difference in the toxicity between the flesh of the anterior and posterior halves of moray eels (*Gymnothorax javanicus*) and red bass. However, there are no data for the distribution of ciguatoxins in the flesh of Spanish mackerel or any reef fish from Australia.

Our model also assumes that the ratio for flesh weight to total weight for fish greater than 0.5-year of age is constant. This appears a reasonable assumption as there is a linear relationship (r^2^ = 0.98) between fillet (flesh) weight and whole weight for Spanish mackerel from Western Australia [162]. However, if the flesh weight to total weight ratio increases over time, then the “dilution” effect of growth will be greater, and our results will underestimate the reduction in fish toxicity over time. The corollary is that if the ratio decreases over time, then the “dilution” effect will be less, and our results will overestimate the reduction in toxicity.

Our model assumes that the accumulation of ciguatoxin does not affect the long-term growth characteristics of Spanish mackerel and therefore the age-weight model for growth is consistent for non-toxic fish as well as fish contaminated with ciguatoxins. However, growth may be affected, at least in the short-term, by accumulation of ciguatoxins as Davin et al. [139] reported behavioural changes in demersal reef fishes (*Epinephelus* and *Lutjanus* spp.) fed extracts from ciguatoxic barracuda, including changes in feeding behaviour. O’Toole et al. [163] suggested that the home-range of ciguatoxic barracuda in the Caribbean may be less than for non-toxic individuals, indicating possible behavioural changes. Ciguatoxins are potent ichthyotoxins [140], that can produce similar electrophysiological effects on fish and mammalian nerves [138], so it would not be surprising if the physiology of Spanish mackerel were affected by feeding on ciguatoxic prey. Lewis [140] suggested that the lethal effects on fish may impose an upper limit on the concentrations of toxins that some fish can accumulate. However, there is conflicting evidence about the effect of ciguatoxins on fishes. Early studies feeding toxic fish flesh to red bass (*Lutjanus bohar*) and an omnivorous surgeonfish (*Acanthurus xanthropterus*) did not report any signs of intoxication [152,164]. More recently, juvenile sea mullet (*Mugil cephalus*) fed gel pellets containing *Gambierdiscus polynesiensis* displayed signs of intoxication with a mix of abnormal hyperactive and hypoactive behaviours [71]. In contrast, juvenile unicornfish (*Naso brevirostris*) fed the same cultured strain of *G. polynesiensis* showed no signs of abnormal behaviour or poisoning [102]. The *G. polynesiensis* strain (TB-92) used for these experiments has a ciguatoxin profile dominated by the P-CTX3C family of toxins but also includes lesser amounts of P-CTX-4A, the precursor to P-CTX-1 and -2 [71,102,103]. Growth was also reduced in juvenile goldspotted rockcod (*Epinephelus coioides*) fed fish-pellets contaminated with P-CTX-1, -2 and -3 [72], and freshwater goldfish (*Carassius auratus*) fed C-CTX-1 became lethargic after 2-weeks of daily ingestion of toxin [73]. Unicornfish (surgeonfish) and goldspotted rockcod (grouper) are fish species that are often found in a coral reef environment typical for ciguatera, whereas sea mullet is an estuarine and coastal species that does not (at least in Australia). Intoxication likely depends upon the dose ingested, the fish species consuming the toxins and possibly the chemical profile of the ciguatoxins being consumed (as well as water-soluble toxins such as maitotoxins ingested by herbivorous and detritivorous fishes but not bio-accumulated across trophic levels). Our models would overestimate the dilution of ciguatoxins from the flesh of Spanish mackerel by somatic growth, if fish growth is reduced because of the physiological or behavioural effects of accumulating ciguatoxins.

Finally, our model assumes that the accumulation of ciguatoxins does not affect the susceptibility of Spanish mackerel to be caught by commercial or recreational fishers (e.g., through enhancing or deterring feeding behaviours as most fish are caught on lines using baits or lures, not by netting). Any increased bias towards catchability would be a mechanism that funnels ciguatera into the human food chain. In contrast, reduced catchability would suggest that the actual incidence rate for ciguatoxic Spanish mackerel in the wild (see [29]), is underestimated.

To simplify the interpretation of our model, we have only modelled fish that have accumulated the indicated ciguatoxin concentration in their flesh from single age points (0.5, 1, 2 and 4 years of age). Any additional toxin uptake after these time points would complicate the modelling, and result in the current models being an underestimate of the time required for dilution of ciguatoxins below the precautionary action concentration of 0.01 µg/kg of P-CTX-1 equivalents suggested by the U.S. Food and Drug Administration (FDA) for the safe consumption of seafood [154].

Our modelling assumes that the severity of ciguatera poisoning suffered by most people is proportional to the concentration of ciguatoxins in the fish flesh eaten [1]. We model four different initial concentrations for contamination of Spanish mackerel flesh (P-CTX-1 equivalents of 5.0, 1.0, 0.1 and 0.03 µg/kg), and assume that people eat a relatively “standard” portion size, and do not intentionally eat small (<50 g) portions as a personal risk minimization measure [1].

We consider a flesh ciguatoxin concentration of 5.0 µg/kg P-CTX-1 equivalents or more as an extremely toxic fish for Australia. This is the maximum ciguatoxin concentration that Lehane and Lewis [1] reported for the range of toxin concentrations found in ciguateric fishes from across the Pacific. However, for our models it is a hypothetical concentration as we do not have data for the maximum concentration of ciguatoxins that can accumulate into the flesh of Spanish mackerel or any Australian reef fish. The highest P-CTX-1 flesh concentrations we know of from Australia are 3.9 µg/kg from the flesh of a Coral Cod (*Cephalopholis miniata*) that caused ciguatera in the Northern Territory [27], 3.5 µg/kg P-CTX-1 from the flesh of a sawtooth barracuda (*Sphyraena putnamiae*) that caused three cases of ciguatera including the death of an elderly male in Queensland [28], and 1.0 µg/kg from a ciguatoxic Spanish mackerel caught from northern New South Wales [14]. The maximum concentration reported from the muscle of fishes from the Pacific Ocean is 81.8 µg/kg of P-CTX-1 equivalents from moray eel (*Gymnothorax* sp.) from the Republic of Kiribati [87]. Such high toxin concentrations are likely life-threatening, with probably more deaths from ciguatera in the Pacific caused by moray eels than any other fish [165]. Possibly moray eels can accumulate such high concentrations through a resistance mechanism [87], not possessed to the same degree by other fish species [140,166,167]. The accumulation of such high ciguatoxin concentrations in moray eels is even more surprising if Lewis et al. [86] are correct with their hypothesis for the depuration of ciguatoxins from fishes. There is no market for moray eels as food fishes in Australia and they are not targeted by commercial or recreational fishers, so there is no basis to assess the toxicity of moray eels from the Great Barrier Reef or east coast of Australia.

We consider highly toxic fish from Australia as having flesh P-CTX-1 concentration of 1.0 µg/kg P-CTX-1 equivalents, i.e., 100-times the U.S. FDA precautionary action concentration of 0.01 µg/kg of P-CTX-1 equivalents. We consider lowly toxic fish as having flesh P-CTX-1 concentration equivalents of 0.1 µg/kg, as Lehane and Lewis [1] estimated that 2 out of 10 people would be poisoned by this concentration. Precautionary action concentrations incorporate a safety margin, so the actual no-adverse-effect concentration likely lies between 0.01 and 0.1 µg/kg of P-CTX-1 equivalents. However, Hossen et al. [168] suggested that Caribbean fishes contaminated with as little as 0.02 µg/kg of P-CTX-1 equivalents of Caribbean ciguatoxins (C-CTX) could cause human poisoning, but this may correspond to a higher toxin load, as C-CTX-1 is less toxic than P-CTX-1 [34]. Toxic fish that cause acute or life-threatening poisonings are rare in Australia [5], although the death of a healthy young woman in Queensland has been attributed to ciguatera from eating Spanish mackerel fillets [142]. Therefore, modelling the dilution of ciguatoxins from the flesh of Australian fishes is probably most relevant for lowly to highly toxic fish. Based upon the symptom profile and time to onset of symptoms, Lewis et al. [76] concluded that mackerel were “on-average” more toxic than non-mackerel species in Queensland.

Ciguatera is uncommon in Australia [5], so repeat poisonings are even rarer. This contrasts with communities with much greater reliance on tropical seafood, such as some Pacific island communities where a fatalistic attitude to ciguatera poisoning can develop [7,136,169]. We have therefore assumed that our modelled concentrations for causing clinical symptoms of ciguatera (≥0.01 µg/kg P-CTX-1 equivalents) is for a naive human population not previously exposed to ciguatera, as it is thought that people who have been poisoned previously can become more sensitive to the ciguatoxins [4].

**Figure 6:**
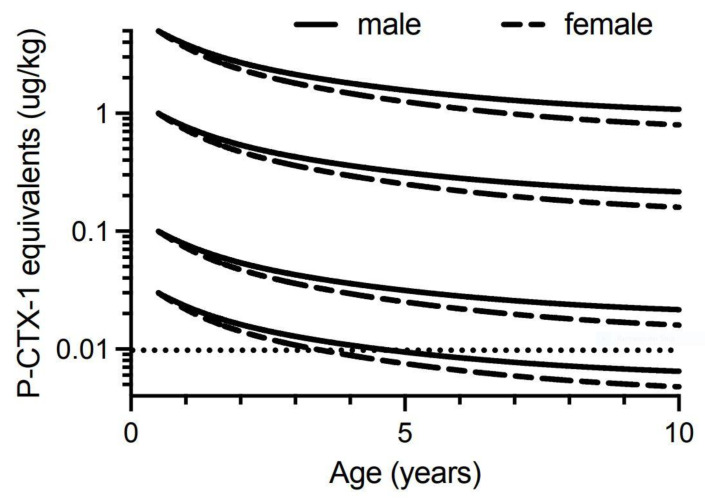
Modelled dilution of 5.0, 1.0, 0.1 and 0.03 µg/kg P-CTX-1 equivalents from Spanish mackerel flesh by somatic growth, for male and female fish contaminated with ciguatoxins at 0.5 years of age. Dotted line = USFDA precautionary action concentration of 0.01 µg/kg of P-CTX-1 equivalents. (See Figure S8 for expanded figure with modelled dilution of fish contaminated with ciguatoxins at 0.5, 1, 2 and 4 years of age).

Our model suggests that for 0.5-year-old Spanish mackerel contaminated with 0.03 µg/kg of P-CTX-1 equivalents, somatic growth could reduce the toxicity of the flesh below the U.S. FDA precautionary action concentration of 0.01 µg/kg equivalents by 4-years of age for female fish, and 5-years for male fish (Figure 6). However, a toxin concentration of 0.03 µg/kg P-CTX-1 equivalents may not always cause human poisoning [1]. We conclude that somatic growth cannot reduce the toxicity of the flesh of fish of ≥0.5-years age contaminated with 0.1 µg/kg P-CTX-1 equivalents (i.e., a lowly toxic fish), to less than the U.S. FDA precautionary level within 10-years (Figure 6 and Figure S8). As most Spanish mackerel caught from along the east coast of Australia are less than 8-years old [83] this effectively means that somatic growth on its own is unlikely to reduce the ciguatoxin burden in the flesh of ciguateric Spanish mackerel of legal size to levels considered safe for consumption. However, for fish that accumulate a significant toxin burden at a young age, the reduction in toxin concentration from rapid growth during its first year (Figure 5), could reduce the toxin concentration sufficiently to reduce the severity of the illness.

## 4. Model for the Dilution of Ciguatoxins in the Flesh of Spanish Mackerel (*S. commerson*) through Growth and Depuration

Our second quantitative model hypothesizes that Spanish mackerel can depurate ciguatoxins. Halstead and Bunker [151] thought that ciguateric fish could detoxify their tissues, but Randall [70] did not. Thirty years later, Tosteson et al. [170] suggested that barracuda (*Sphyraena barracuda*) may be able to detoxify ciguatoxins based upon a seasonal reduction in frequency of toxic fish caught in the Caribbean. Populations of ciguateric surgeonfish were reported to lose toxicity over several months in French Polynesia [94], and Lewis et al. [86] suggested moray eels from Tarawa in the Republic of Kiribati could depurate ciguatoxins with a half-life of <1 year. Li et al. [72] have recently demonstrated rapid depuration of ciguatoxins from the muscle of juvenile goldspotted rockcod (*Epinephelus coioides*), with depuration consistent with a mono-phasic exponential decay, and half-lives for P-CTX-1, -2 and -3 of 28, 26 and 33 days, respectively. Previously, Ledreux et al. [71] were the first to experimentally demonstrate the depuration of ciguatoxins from the muscle of fishes. They found that juvenile sea mullet (*Mugil cephalus*) fed a gel diet containing *Gambierdiscus polynesiensis* depurated ciguatoxins from muscle tissue with a half-life in hours. The depuration of ciguatoxins from muscle was suggested to be through the liver via the bile to the intestine [71]. However, it is likely that the depuration rates for xenobiotics such as ciguatoxins are faster in juvenile fish compared to those of adults. In humans, the half-life for pharmacokinetic clearance of drugs can be faster in children than adults [171]. It is difficult to see how ciguatera would be a global health problem if ciguatoxins could depurate from the muscle of large fish with a half-life of <1 day.

We find the use of sea mullet (*M. cephalus*) as a model for the uptake and depuration of ciguatoxins [71] interesting as sea mullet is one of the most important commercial fisheries in Australia with the annual catch exceeding that of all other fin-fish species in New South Wales and Queensland [172]. Along the east coast of Australia, sea mullet is commercially fished from southern New South Wales to just north of Hervey Bay, including the north-western beach of Fraser Island to the immediate north of Platypus Bay (Queensland Department of Agriculture and Fisheries, personal communication). In recent years, the most valuable part of the mullet fishery is for the ovaries (roe) which are exported to East Asia [173] for production of high value comestibles such as Karasumi/Bottarga. Sea mullet are a species never associated with ciguatera in Australia and given that the viscera of ciguateric reef fishes can be up to 50-fold more toxic per unit mass than the flesh [43,161], it is likely that we would know of poisoning cases from mullet flesh or roe if they occurred. The depuration of ciguatoxins from sea mullet reported by Ledreux et al. [71] suggests that the enzymatic pathways responsible for depuration are common to many fish species including those not normally associated with ciguatera. Ikehara et al. [25] have shown that liver enzymes from both toxic and non-toxic fish species can bio-oxidize ciguatoxin P-CTX-4A to its more toxic form (P-CTX-1), as well as produce M-*seco*-analogs, which they interpreted as liver detoxification products.

Yogi et al. [31] suggested the absence of P-CTX3C (Figure 1) from a range of predatory reef fish from Okinawa containing P-CTX-1, could indicate that the local *Gambierdiscus* did not produce the P-CTX3C family of toxins. Alternatively, P-CTX3C analogs may depurate rapidly from higher trophic level fish as suggested by Ledreux et al. [71]. However, both P-CTX-1 and P-CTX3C analogs have been detected from red bass (*Lutjanus bohar*) [31], amberjack (*Seriola dumerili*) [174] and moray eel (*Gymnothorax javanicus*) [22], showing that both families of toxins can bio-accumulate into higher trophic level fishes. Oshiro et al. [158] could not detect P-CTX3C from five yellow-edge coronation trout (*Variola louti*) caught from Okinawa and contaminated with P-CTX-1, -2 and -3. As yet, P-CTX3C analogs have not been detected from dinoflagellates or fish from Australia.

**Figure 7:**
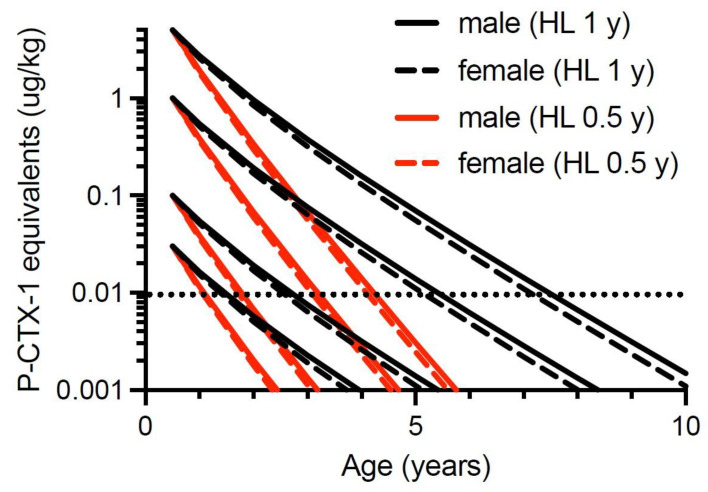
Modelled dilution of 5.0, 1.0, 0.1 and 0.03 µg/kg P-CTX-1 equivalents from the flesh of male and female Spanish mackerel of 0.5 years of age by a combination of somatic growth and depuration. Red lines = depuration half-life (HL) = 0.5 year. Black lines = depuration half-life (HL) = 1 year. Dotted line = USFDA precautionary action concentration of 0.01 µg/kg of P-CTX-1 equivalents. (See Figures S9–S12 for expanded figure with modelled dilution from somatic growth and depuration for fish contaminated with ciguatoxins at 0.5, 1, 2 and 4 years of age, and with hypothetical half-lives for depuration of 0.5, 1, 2 and 4 years).

Lewis et al. [86] suggested that Pacific moray eels from the Republic of Kiribati could depurate ciguatoxins with a half-life of <1 year. We constructed our second model for dilution of ciguatoxins in the muscle (flesh) of Spanish mackerel using a combination of growth and depuration. We have modelled a range of potential half-life’s, from 0.5 to 4 years (Figure 7 and Figures S9–S12). We recognize that this modelling is speculative without any direct evidence for depuration in Spanish mackerel. However, there is circumstantial evidence for depuration, and we cautiously interpret the modelling results in the context of the biology and fishery for Spanish mackerel and the frequency of ciguatera cases from the east coast of Australia. Spanish mackerel are heavily exploited along the east coast of Australia with unsustainable total annual catches (~1000 tonnes) occurring in the late 1970’s and early 1980’s, and again in the early 2000’s, with commercial catches remaining high at other times before the introduction of a total allowable commercial catch in 2004 [83]. Given this high exploitation rate, we hypothesize that after the ban on taking Spanish mackerel from Platypus Bay came into force in 1987, which appeared to produce a reduction in the frequency of ciguatera along the east coast of Australia, fish that accumulated ciguatoxins in Platypus Bay, left the bay and depurated ciguatoxins relatively quickly before they were caught elsewhere or died of natural causes. Alternatively, it is possible that the ban may have coincided with a long-term decrease in ciguatoxin production from Platypus Bay. Even without direct evidence for depuration of ciguatoxins in Spanish mackerel, our model provides a conceptual pilot for the use of fishery science in developing such risk assessments. Our model for depuration assumes that the depuration rate is constant over the lifetime of adult fish as we have no basis for considering the up- or down-regulation of the enzymes involved in depuration pathways. We have used the same four initial ciguatoxin concentrations that we used for our previous growth dilution model (5.0, 1.0, 0.1 and 0.03 µg/kg P-CTX-1 equivalents).

Our model suggests that somatic growth in combination with a hypothetical half-life for ciguatoxin depuration of six months could reduce the toxicity of the flesh of even an extremely toxic (5.0 µg/kg P-CTX-1 equivalents) six-month-old fish to below the U.S. FDA precautionary action threshold (0.01 µg/kg P-CTX-1 equivalents) by the time the fish reaches 5 years of age (Figure 7). This is for both male and female fish which would weigh ~9.5 and ~12.4 kg at 5-years of age, respectively (Figure 5). In contrast, the model suggests that with a half-life of 1-year, although the 5.0 µg/kg toxin concentration would quickly reduce, the flesh concentration would not drop below the 0.01 µg/kg threshold for male or female fish until they were between 7- and 8-years of age (Figure 7). As most commercially caught Spanish mackerel are less than 8-years of age [83], this would effectively be for most Spanish mackerel caught from Queensland. A half-life of 4-years would effectively mean that a highly toxic 0.5-year-old fish may not become safe to eat over most of its lifetime (Figure S12), and even a 0.5-year-old lowly toxic fish (0.1 µg/kg P-CTX-1 equivalents) would be between 5- and 7-years of age before it reached the U.S. FDA threshold concentration (Figure S12a). All these conclusions are based upon the assumption that the fish does not accumulate any additional ciguatoxin after six months of age as this would significantly delay the time for the fish to reach a flesh concentration that was safe to eat.

We can use the relationships shown in Figure 7 and Figures S9–S12 to estimate the half-life most consistent with ciguatera outbreaks in southern Queensland and northern New South Wales for low and high ciguatoxin concentrations contaminating Spanish mackerel to reach the FDA threshold concentration of 0.01 µg/kg P-CTX-1 equivalents (although the safe-level for consumption is likely between 0.01 and 0.1 µg/kg P-CTX-1 equivalents). Ciguatera cases caused by Spanish mackerel decreased in Queensland after the 1987 ban on their capture from Platypus Bay, possibly indicating that fish had depurated toxicity before being caught elsewhere or dying of natural causes. If we assume commercial fishers were catching 2- and 3-year old Spanish mackerel from Platypus Bay before the ban (Table 2), and we assume an initial P-CTX-1 concentration of 1.0 or 0.1 µg/kg equivalents contaminating Spanish mackerel [14,26], then it would take a further ~10–11 years before 2- or 3-year old highly-toxic fish contaminated with 1.0 µg/kg P-CTX-1 equivalents would reach the U.S. FDA threshold concentration with a depuration half-life of 2-years (Table 2). For lowly toxic fish (0.1 µg/kg P-CTX-1 equivalents), it would take between a further 4 and 6 years (Table 2). As most Spanish mackerel caught in Queensland are less than 8-years of age [83], a half-life of 2-years or longer for depuration seems inconsistent with the observed reduction in ciguatera cases from Spanish mackerel after 1987. In contrast, a half-life of 1-year would take between a further 2 and 6 years for 2- and 3-year-old fish to depurate 1.0 or 0.1 µg/kg P-CTX-1 equivalents to the U.S. FDA threshold concentration (Table 2), and a half-life of 0.5-year would take between a further 1 and 3 years (Table 2). If Spanish mackerel along the east coast of Australia can depurate ciguatoxins, then we believe that it is most likely with a half-life of 1-year or less, because this rate of depuration is consistent with the incidence of ciguatera from Spanish mackerel reducing after the enactment of the ban on their capture from Platypus Bay. A half-life of ≤1-year is also consistent with the depuration rate suggested for moray eels by Lewis et al. [86].

The circumstantial [86,151,170,175] and experimental [71,72] evidence for depuration of ciguatoxins in fishes is strong, but the rate of depuration is likely to vary between tissues, species, life stage, toxin profile and metabolic regulation of detoxifying enzyme levels. Given the co-occurrence of rabbitfish and blotched-javelin feeding within the same ciguatoxic food web in Platypus Bay, but the former being generally non-toxic, it is possible that blotched-javelin depurate ciguatoxins more slowly than rabbitfishes. We have hypothesised that Spanish mackerel can depurate ciguatoxins, but ideally this requires experimental proof. Unfortunately, Spanish mackerel, especially larger specimens, do not often survive capture and release [144], so capturing commercial-sized fish and keeping them alive for direct feeding and depuration experiments may be difficult. In such cases, the use of fisheries science to model depuration as used in this review, helps provide a conceptual framework for future hypothesis testing.

If ciguatoxic fish can develop mature gonads, then spawning could be another mechanism for dilution of ciguatoxins from fish. The relative toxicity of red bass ovaries was reported to be slightly greater than muscle (flesh), but testes were more than tenfold more toxic [160]. Colman et al. [176] suggested that barracuda can transfer Caribbean ciguatoxin (C-CTX-1) to their eggs at much higher concentrations than found in muscle, and Yan et al. [177] have recently demonstrated transfer of P-CTX-1 to eggs in marine medaka (*Oryzias melastigma*). Prior to spawning, the gonads can contribute a significant amount to the total weight of Spanish mackerel with ovaries being up to 13% of fish body weight, and testes up to 7% of fish body weight [156]. Spawning occurs by the broadcasting of eggs and sperm into water, which may quickly shed a considerable tissue burden of toxin from the fish. It is interesting to speculate if such a fish survives until the next spawning season, would the re-maturing gonads again accumulate ciguatoxins from any residual toxin in other tissues? The embryos produced by broadcast sperm and eggs from spawning ciguateric fishes may not survive as ciguatoxins have deleterious effects on developing fish embryos [176,177,178,179].

## 5. A conceptual Model for Ciguateric Food-Chains on the Great Barrier Reef

The Great Barrier Reef extends offshore of the east coast of Queensland from the northern tip of Cape York to Bundaberg in the south, is about 2300 km long and made up of ~3000 separate reefs, ~600 mainland islands and ~300 coral cays. It sits within the Great Barrier Reef Marine Park, which covers an area of ~344,400 km^2^ consisting of 70 broadscale habitats (bioregions) and was enacted in 1975 and World Heritage Listed in 1981. Our conceptual model for ciguatera food-chains on the Great Barrier Reef is simplistic given the complexity of habitats and communities produced through spatial gradients across the marine park, both in a general east-west direction across the continental shelf from in-shore to mid-shelf and outer-shelf reefs, as well as the large north-south latitudinal gradient. For example, predatory fish assemblages differ markedly across the continental shelf with some but weaker differences along the north-south latitudinal gradient [180].

No individual reefs or regions are known that regularly produce ciguateric fishes, and none are known that produce more toxic fish than any other reef or region. Ciguatera cases are mostly caused by demersal predatory fishes caught from apparently anywhere along its length or breadth [5,6,181]. There is also almost no information on the production and transfer of toxins across trophic levels along the Great Barrier Reef. We know more about the food chain transfers that cause ciguatera from Platypus Bay fishes than from the Great Barrier Reef. Without more information, the food chains discussed in this review for the development of ciguatera along the Great Barrier Reef are hypotheses based mostly upon the general models developed for Pacific Island nations and territories (e.g., [70]) and contextualised within the food chains and reef fish fisheries found on the Great Barrier Reef.

In French Polynesia, herbivorous fishes are often more toxic than their higher trophic level predators, and there is often a considerable delay after the herbivorous fishes in a region become toxic before the predatory fishes become toxic [136,137,182]. This delay has been interpreted as a time-dependant transformation of analogs such as P-CTX-4A in the food chain before the appearance of P-CTX-1 in higher trophic level fishes [71]. An alternative hypothesis is that depuration of ciguatoxins could delay the accumulation of significant toxin concentrations in fishes exposed to ongoing low-concentration sources of toxins. A similar situation may exist in the Cook Islands where four of the top five fishes that cause ciguatera are herbivores [128]. The higher toxicity of herbivorous fishes in French Polynesia contrasts with the much greater toxicity of higher trophic level fishes in the Republic of Kiribati [32,87].

Herbivorous reef fishes generally have a “stronger” flavour than demersal carnivorous fishes and in many Pacific cultures this can be a desirable characteristic, whereas in Australia, herbivorous fishes from the Great Barrier Reef are generally not preferred and are rarely targeted by recreational or commercial fishers [183,184], with less than 4 tonnes of rabbitfish harvested by commercial fishers from the entire Great Barrier Reef marine park between 1990 to 2019 [141]. This is unlikely to change in the future with the organization responsible for managing the Great Barrier Reef Marine Park (GBRMPA), now actively discouraging the harvesting of rabbitfishes, parrotfishes and surgeonfishes (including unicornfishes) to reduce macroalgal growth on reefs impacted by coral bleaching and crown of thorns starfish (http://www.gbrmpa.gov.au/__data/assets/pdf_file/0006/247848/Coral-Recovery-A4-Flyer_4Print.pdf (accessed on 20 July 2021)). We therefore have no basis for comparison of the relative frequencies of toxic herbivorous and carnivorous fishes or the concentrations of the ciguatoxin congeners that are accumulated by them.

### 5.1. Food Chain Links Leading to Ciguatera along the Great Barrier Reef, Trophic Level 1

Benthic dinoflagellates, including *Gambierdiscus* species are common epiphytes on many macroalgae and turf algae (the epilithic algal matrix/community) found along the Queensland coast and the Great Barrier Reef [15,90,175,185]. They are mostly found in low population densities and on all major classes of macroalgae [15,185,186]. Six *Gambierdiscus* and one *Fukuyoa* species have been so far confirmed from the waters of the Great Barrier Reef (Table S1), with *G. carpenteri* [93] and *F. paulensis* [187] also isolated from New South Wales. Of these species, low concentrations of ciguatoxins have been detected from cultures of *G. carpenteri* [47], *G. belizeanus* [45,47,188] and *F. paulensis* [189] isolated from regions outside of Australia. However, known ciguatoxins could not be detected from cultures of *G. carpenteri*, *G. lapillus*, *G. lewisii* or *G. holmesii* isolated from the Great Barrier Reef [107,190,191], or *G. carpenteri* from New South Wales [93]. Larsson et al. [107] did report finding ciguatoxin-like activity from *G. lewisii* and *G. holmesii*, and trace amounts from *G. lapillus*, all isolated from Heron Island on the southern Great Barrier Reef (*G. lewisii* and *G. holmesii* were reported as unidentified species by Larsson et al., [107], with the species subsequently described by Kretzschmar et al., [191]). Earlier studies from the Great Barrier Reef detected ciguatoxin by mouse bioassay from a cultured clone of *Gambierdiscus* isolated from Arlington Reef adjacent to the Wet Tropics region in north Queensland [68]. At the time, *Gambierdiscus* was a monospecific genus so Holmes et al. [68] attributed the clone to *G. toxicus*, but with the description of many new species in recent years (Table S1), we are unable to assign this clone to a species. As yet, we don’t know which species from the Great Barrier Reef are responsible for producing P-CTX-4A, the precursor to P-CTX-1 and P-CTX-2, the major toxins that cause ciguatera along the east coast of Australia [26,28,29].

Most monitoring programs for benthic dinoflagellates, including those in Australia, have been based upon sampling a diverse array of benthic foliose or calcareous macroalgal species and expressing the epiphytic dinoflagellate cell density shaken from these substrates per gram of wet weight macroalgae [192,193]. Adaptations of this method have been widely adopted because it is easy to use. However, unless the same macroalgal species is sampled from different locations, or repeatedly from the same location, cell density comparisons based upon substrate weight are of limited value. For epiphytic species, cell density per unit surface area of substrate is probably more useful as a basis for comparison of populations, especially given the dramatic differences between surface area and unit weight of macroalgae [194]. The use of artificial substrates (settling plates) may overcome many of these deficiencies and provide a better basis for spatial and temporal comparisons [117,195,196,197,198], but have not yet been used for monitoring on the Great Barrier Reef. In locations such as Platypus Bay where a single substrate (*Cladophora*) is known to support the source of ciguatoxins, cell densities based upon wet weight of macroalgae are sufficient for internal comparisons, but if a standardised assessment method can be validated [196,198,199] it would allow for better comparisons between sites. However, Parsons et al. [200,201] suggest caution as they found poor correlation of *Gambierdiscus* densities on artificial substrates and macroalgae in the Florida Keys and U.S. Virgin Islands; although, from a ciguatera perspective, what is important is whether the populations being quantified are those likely to enter food chains leading to ciguatera.

There are 600–700 species of macroalgae on the Great Barrier Reef and they have considerable latitudinal, cross-shelf and within reef variation [202]. In contrast to inshore reefs, offshore reefs usually have low standing biomass of foliose macroalgae, but high cover of crustose calcareous algae and turf assemblages [202]. Where foliose macroalgae do survive on coral reefs, it is often because of their unpalatability to herbivores [203] or where herbivore behaviour is modified through other mechanisms, such as fear of predation [204,205,206]. Macroalgae may act as “reservoirs” for epiphytic dinoflagellates on coral reefs, but unless they and their epiphytes are consumed in what Cruz-Rivera and Villareal [203] termed their shared doom, they do not become part of the food chain leading to ciguatera. In the Cook Islands, increased ciguatera cases were associated with increased turf algae cover on coral reefs, whereas decreases in ciguatera cases occurred during a period of increased macroalgal cover [207]. Turf assemblages are ubiquitous and diverse on the Great Barrier Reef where 1 cm^2^ of space can host more than 20 species [202] and they can be 15 times more productive than foliose macroalgae on reefs [208]. They are one of the most abundant benthic habitats on the Great Barrier Reef [209] and are grazed by herbivorous and detritivorous fish, and invertebrates. We believe that epiphytic *Gambierdiscus* or *Fukuyoa* on turf algae are the most likely to enter food chains leading to ciguatera on many reefs [33,197,198,201,203], including the mid- and outer-shelf reefs of the Great Barrier Reef, but this is yet to be tested. Lewis et al. [175] developed an air-lift suction device to sample 0.8 m^2^ of turf algae for benthic dinoflagellates, in part to mimic the feeding process of the detritivorous, lined-bristletooth surgeonfish (*Ctenochaetus striatus*), one of the major vectors of ciguatoxins on Pacific reefs [210]. Parsons et al. [96] used a similar monitoring approach to sampling turf algae in Hawaii. While the method is suitable for sampling turf algae for benthic dinoflagellates, a way to compare population densities between sites still needs to be validated, possibly in conjunction with direct monitoring of dissolved ciguatoxins from the water [211,212,213].

Runoff of fine sediments and nutrients from land catchments are considered major threats to the health and resilience of the Great Barrier Reef [114]. Nutrients have been hypothesized to promote the growth of macroalgae and crown-of-thorns starfish outbreaks, and fine sediments reduce light and can smother seagrass meadows and inshore coral reefs [114]. To reduce the impacts of these stressors, targets have been developed for reducing the end-of-catchment loads of nutrients and sediments, and concentrations of pesticides (mostly herbicides) that flow from the major river catchments into the Great Barrier Reef lagoon (Great Barrier Reef 2050 Water Quality Improvement Plan, https://www.reefplan.qld.gov.au/ (accessed on 20 July 2021)). While there have been major improvements in land practices in recent years (Great Barrier Reef Report Card for 2017 and 2018, https://www.reefplan.qld.gov.au/tracking-progress/reef-report-card/2017-2018 (accessed on 20 July 2021)), at present they are not happening fast enough to be able to meet the 2050 targets [114].

Most catchment sourced nutrients and sediments flow to the Great Barrier Reef lagoon during high rainfall events during the monsoon (wet) season [214]. Fine sediments (<16 µm) can be carried in suspension by flood plumes and reach inshore and sometimes mid- and outer-shelf reefs and be easily resuspended by wind and wave action [215]. Whether sediments and nutrients reach mid- or outer-shelf reefs depends upon the size of the contributing catchments, the size of the flood plumes generated and the distance from river mouths to mid- and outer-shelf reefs. In addition, south-easterly winds and the Coriolis force will tend to push flood plumes northwards along the coast. However, in the Wet Tropics NRM region of north Queensland (from north of Townsville to south of Cooktown, approximately 15.5° S to 18.5° S, Figure S7), the continental shelf is narrow and nutrients and fine sediments probably reach mid-shelf reefs most years [216]. Riverine inputs dominate nutrient inputs to inshore reefs in the Wet Tropics, but upwelling, mixing events and nitrogen-fixation are more important for offshore reefs in the Wet Tropics [214]. In the relatively oligotrophic waters of the Great Barrier Reef, increased nitrogen concentrations are short-lived due to rapid biological uptake [214]. Benthic dinoflagellates living within turf algae matrices may face considerable competition for nitrogen resources given the rapid assimilation of inorganic nitrogen by their host algae [217,218]. However, some *Gambierdiscus* species may partially offset these limiting inorganic nitrogen concentrations through mixotrophy [219,220].

Sedimentation can drive declines in the productivity of algal turfs as well as suppress feeding on them by herbivores such as surgeonfishes [221,222,223,224], with feeding by *C. striatus* especially sensitive to small increases in sedimentation [225]. Sediments can cause the length of turf algae to increase while reducing their productivity and the proportion of detrital particulates within them [226]. The filling of interstitial spaces of turf algae by sediments not only limits feeding on them by surgeonfishes [222,224,225], it may also reduce space for growth of benthic dinoflagellates, with fine sediments (<16 µm) being smaller than the minimum diameter of *Gambierdiscus* and *Fukuyoa* species. Both the reduction in habitat for benthic dinoflagellates and in herbivory caused by deposition of sediments could be mechanisms that limit the production of ciguatoxins into coral reef food chains. Sediment loads on inshore reefs can overwhelm turf algae [224,227], and deter grazing by parrotfish on inner-shelf reefs where they are often the dominant herbivore/grazer [228]. It would be ironic if the major investment by Australian and Queensland Governments to restore the health of the Great Barrier Reef led to reductions in sediment loads that produced more grazing of turf algae supporting populations of *Gambierdiscus*. However, there are many links in marine food chains, and it is not possible to predict what the outcome would be from any such change with respect to the frequency or severity of ciguatera.

Reducing mixtures of pesticides flowing off agricultural catchments into the Great Barrier Reef lagoon during high rainfall events is a priority action for improving the health and resilience of the Great Barrier Reef, especially for certain catchments in the Mackay-Whitsunday, Burdekin and Wet Tropics NRM regions (Great Barrier Reef 2050 Water Quality Improvement Plan, https://www.reefplan.qld.gov.au/ (accessed on 20 July 2021)). While the impacts of pesticides most likely occur on inshore reefs, in the Wet Tropics NRM region, the photosystem II inhibitor diuron may reach more than 40 km offshore from river mouths at concentrations with inhibitory effects [229]. Shaw et al. [230] reported that herbicides could be transported in plumes at concentrations that affected zooxanthellae dinoflagellates on inshore coral reefs. Community composition changes in tropical benthic microalgae communities can occur at low µg/L concentrations of diuron [120]. However, there is not enough information to know if catchment derived herbicide concentration could be having an impact on populations of *Gambierdiscus* and other benthic dinoflagellates along the Great Barrier Reef.

*Halimeda* banks (bioherms) are deep water reefs (>20 m) with a surface layer of live *Halimeda* adjacent to the inside of outer barrier reefs of the Great Barrier Reef and covering many thousands of km^2^ [209,231]. *Halimeda* species are calcareous green macroalgae also common on shallow reefs along the Great Barrier Reef [202], which often host low population densities of epiphytic benthic dinoflagellates, including *Gambierdiscus* [90,185,186]. It is possible these deep-water reefs may be a reservoir for benthic dinoflagellate populations, but we are unaware of any sampling from these banks or other deep-water habitats. *Gambierdiscus* species are photosynthetic but may be able grow under low light conditions by acquiring nutrients through mixotrophy [219,220]. Herbivorous fish are rare below 50 m off the Great Barrier Reef shelf-break, but macroalgae have been found to 194 m depth and *Halimeda* to 150 m [232].

It is possible that there may be more than one source of ciguatoxins produced among the diverse microalgal species growing amongst the turf community matrix, as bio-synthetic pathways can be common to different algal groups. For example, paralytic shellfish poisoning toxins are produced by a diverse mixture of dinoflagellate and cyanobacterial species [233], and 44-methylgambierone has been isolated from species of *Gambierdiscus*, *Fukuyoa* and *Coolia* [60,61,62,63,124]. Ciguatoxin-like activity has been reported from the planktonic cyanobacteria *Trichodesmium* [234,235] but there has been little follow-up of these results. The recent suggestion that parrotfishes are microphages that acquire their nutrition by feeding mainly on photosynthetic microorganisms, predominantly cyanobacteria that are epilithic, epiphytic, endolithic or endosymbionts [236,237] requires further study with respect to their accumulation of toxins that cause ciguatera. Laurent et al. [238] previously linked ciguatoxin-like activity extracted from parrotfish and cyanobacterial mats from New Caledonia, in the apparent absence of *Gambierdiscus*.

*Gambierdiscus* is often only one of several toxin-producing benthic dinoflagellates found as epiphytes on macroalgae and turf algae on coral reefs. Due to this, many authors have suggested that these other species may play a role in ciguatera, but benthic dinoflagellate toxins are only part of the milieu of secondary metabolites produced on coral reefs [239]. For example, we found that between 50–100% of the solvent extracts of different sized fractions sieved from epiphytes and detritus from macroalgae and turf algae collected from along the east coast of Queensland were lethal to mice by intraperitoneal injection [77,91,175]. However, without evidence for their accumulation into the flesh of carnivorous reef fishes, there is no basis for suggesting these toxic fractions have a role in human poisoning along the east coast of Australia.

*Ostreopsis*, *Prorocentrum*, *Amphidinium* and *Coolia* are dinoflagellate genera containing toxic benthic species often found with *Gambierdiscus* as epiphytes on macroalgae on the Great Barrier Reef [15,90,125,126,185,186,240]. *Prorocentrum lima* is often found with *Gambierdiscus* and produces analogs of okadaic acid which are lipid-soluble toxins also produced by several planktonic dinoflagellate species of *Dinophysis* [241,242]. Shellfish accumulate these toxins by filter feeding on toxic *Dinophysis* which causes the disease Diarrhetic Shellfish Poisoning [243]. Okadaic acid has been reported from the flesh of a ciguatoxic Caribbean barracuda [244] but this result has never been repeated from a toxic fish and requires validation. However, okadaic acid has been shown to accumulate in some temperate fish species (reviewed by [245]). Some isolates of *Ostreopsis siamensis*, *O. ovata* and *O. mascarenensis* produce analogs of the extremely potent water-soluble toxin called palytoxin [246,247,248], as does the cyanobacteria *Trichodesmium* [249]. Tropical planktivorous Clupeid fishes such as herrings and sardines as well as various invertebrates are thought to sometimes accumulate palytoxins in their viscera and/or gut [250,251]. Some communities eat Clupeid fishes’ whole, exposing them to the risk of the visceral and gut contents of the fish, and tropical clupeids contaminated with palytoxin analogs have been linked to human poisoning [250,251]. The disease is called clupeotoxin poisoning to distinguish it from ciguatera. It is much rarer than ciguatera but apparently has higher morbidity [250]. We detected a water-soluble toxin from *O.* c.f. *siamensis* isolated from the Great Barrier Reef [240] and palytoxin-like toxins have been detected from *O*. c.f. *siamensis* strains isolated from along the east coast of Australia [252,253]. Relatively high populations of *Ostreopsis* have been found on macroalgae from inshore and mid-shelf reefs of the Great Barrier Reef [15,185].

### 5.2. The Food Chain Links Leading to Ciguatera along the Great Barrier Reef, Trophic Level 2

There is considerable species richness (178 species in 9 families) of herbivorous and nominally herbivorous (e.g., detritivorous) fishes on the Great Barrier Reef [254]. The surgeonfishes comprise a major group of these herbivores, especially on mid- and outer-shelf reefs [255]. The lined-bristletooth (*Ctenochaetus striatus*) and the brown surgeonfish (*Acanthurus nigrofuscus*) are among the most abundant herbivorous fish on Indo-Pacific reefs, including the Great Barrier Reef [255,256,257]. *Ctenochaetus striatus* has long been considered a major vector for ciguatoxins in the Pacific [210], as well as also causing ciguatera poisoning where it is eaten [128,258] and responsible for up to 65% of ciguatera cases in Tahiti [259]. It is a detritovore that does not consume macroalgae and uses its bristle-like teeth to comb or brush algal turfs and consumes considerable quantities of detritus and sediment [257,260], including epiphytic benthic dinoflagellates [42,43]. In contrast, the teeth of *A. nigrofuscus* are designed for cropping and consuming turf algae [257], resulting in the presumed consumption of the associated epiphytic dinoflagellates. To date, the direct evidence for consumption of *Gambierdiscus* by surgeonfish in the wild is the initial research by Yasumoto et al. [42,43] in the Gambier Islands of French Polynesia, and *Gambierdiscus* being found in the gut contents of *C. strigosus* from Hawaii [261]. Magnelia et al. [262] found that Atlantic Ocean surgeonfish (*A. bahianus* and *A. chirurgas*) did not avoid eating *Gambierdiscus* when offered a choice of foods, suggesting that at least some surgeonfish do not find *Gambierdiscus* unpalatable.

At Heron Island, two species of *Ctenochaetus*, *C. striatus* and *C. binotatus* are the most abundant grazers on turf algae with *C. striatus* more abundant in shallow waters [263]. Heron Island is part of the Capricorn Bunker group of coral atolls in the southern Great Barrier Reef (Figure S13) and is a healthy reef system that at least up until 2019, has been spared many of the major issues impacting other sections of the Great Barrier Reef, coral bleaching, damage from cyclones and crown-of-thorn-starfish outbreaks [209]. Marshell and Mumby [263] found that surgeonfish feeding on turf algae at Heron Island make up 74% of the herbivorous fish biomass and remove 73% of the daily productivity of the turf algae in the most productive habitat for turf algae. However, the turfs quickly recover because of their fast growth rates. However, given that the standing crop appears to be constant without major differences between habitats, Marshell and Mumby [263] suggest that grazers concentrate their feeding in habitats where turf algae are most productive. Generally, fish grazer biomass on the Great Barrier Reef correlates more strongly with turf algae production than with turf biomass [264].

There are only limited studies of benthic dinoflagellates on turf algae on the Great Barrier Reef [90,175,186] but these found that *Gambierdiscus* were often present. This suggests that benthic dinoflagellate growth rates, possibly along with imports from local population reservoirs such as those on calcareous and foliose macroalgae, are enough to maintain cell populations on turf algae with the daily removal and/or “combing” of much of the daily productivity of these by surgeonfishes. *Gambierdiscus* species are slow growing, with growth rates under laboratory conditions up to ~0.65 divisions/day [265], although most reported growth rates are considerably slower than this [45,46,266,267,268]. Growth rates for *G. carpenteri* isolated from the Great Barrier Reef were up to 0.17 divisions/day [269] while Holmes et al. [52] reported up to 0.25 divisions/day for a *Gambierdiscus* isolate from the Great Barrier Reef. Growth is dependent on many interacting environmental factors including temperature, light, and nutrients, all of which can vary with depth and aspect on the reef. Mustapa et al. [270] reported growth rates for Malaysian *Gambierdiscus* isolates growing in culture on turf algae that ranged between ~0.1 to <0.3 divisions/day. Growth rates of 0.1–0.25 divisions/day (i.e., population doubling every 4–10 days) could possibly maintain a *Gambierdiscus* population on turf algae if, as suggested by Marshell and Mumby [263] and references therein, that turf algae biomass can be turned over by grazers every 4 to 25 days (especially as reported growth rates for *Gambierdiscus* are often from experiments trying to produce optimal laboratory growth conditions). High grazing rates may be a feedback mechanism that keep low benthic populations from dramatically increasing. Even though grazing is unlikely to be 100% efficient at removing epiphytic benthic dinoflagellates from turf algae, high grazing pressure would result in only a trickle of ciguatoxin entering into the second trophic level, assuming the *Gambierdiscus* species on the turf algae produce ciguatoxins. Any possible depuration of ciguatoxins from surgeonfish [175] could then further limit toxin transfer to higher trophic levels. The dramatic increase in abundance of *Gambierdiscus* found in the US Virgin Islands when turf algae was caged to exclude grazers provides experimental support for this hypothesis [117].

**Figure 8:**
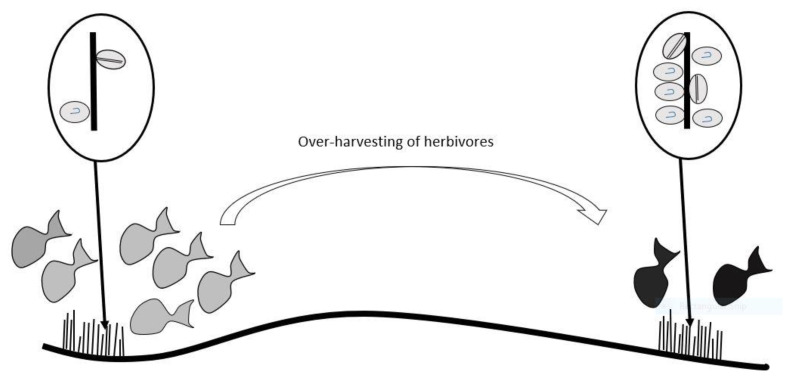
Conceptual model for hypothesised transfer of ciguatoxins from ciguatoxin-producing *Gambierdiscus* or *Fukuyoa* growing as epiphytes on turf algae before and after overharvesting of herbivorous fishes (especially surgeonfishes). On the left, grazing from surgeonfishes prevent benthic dinoflagellate populations from blooming (as indicated by the low number of dinoflagellates shown in the magnified view of turf algae in the oval frame). This limits the amount of ciguatoxin being transferred to the many herbivorous fishes grazing on the turf community and individual fish are generally less toxic (indicated by lighter shading of fish). Overharvesting of surgeonfishes produces the outcome on the right, where surgeonfish abundance is reduced, allowing space and time for benthic dinoflagellate populations to bloom. A higher ciguatoxin load can then be accumulated by the smaller surgeonfish population producing more toxic individual fish (indicated by the darker shading of fish).

If populations of turf algal grazers were rapidly reduced, for example through fishing, then the grazing rate on turf algae might be significantly reduced. This could allow time for populations of benthic dinoflagellates to increase on the turf algae, as found in the US Virgin Islands when grazers were excluded by caging turf algae growing on sandstone tiles [117]. If these populations consisted of species of *Gambierdiscus* and/or *Fukuyoa* that produce ciguatoxins, then this could lead to increased loads of ciguatoxins being available to flow into the remaining, smaller populations of herbivores/detritovores. High grazing pressure on turf algae in natural (healthy) reef ecosystems could limit the opportunity for production of significant ciguatoxin loads, while low grazing pressure, possibly from overharvesting of herbivores, might allow for the development of smaller numbers of highly toxic herbivores (Figure 8). This feedback hypothesis could be partially explored through field cage experiments on turf algae to exclude herbivores similar to those used by Loeffler et al. [117]. Such experiments are often used for studying turf algae productivity [221,223,226,263,264], but have not yet been used on the Great Barrier Reef to study changes in epiphytic benthic dinoflagellate populations on turf algae in the presence or absence of macro-grazers. Using artificial surfaces to develop flat turf layers for cage experiments could also make it simpler to standardize benthic dinoflagellate counts using an underwater vacuum or similar device [84]. If our hypothesis is correct, it would provide more support for the campaign by the Great Barrier Reef Marine Park Authority to discourage the harvesting of herbivorous fishes from the Great Barrier Reef (www.gbrmpa.gov.au/__data/assets/pdf_file/0006/247848/Coral-Recovery-A4-Flyer_4Print.pdf (accessed on 20 July 2021)).

Surgeonfishes, including *Ctenochaetus striatus* are harvested for food in the Cook Islands although they are considered high-risk for ciguatera [128]. Between 2006 and 2011, after major disturbances to coral reefs from crown-of-thorns outbreaks and several severe cyclones, macroalgae cover increased, turf algae cover and surgeonfish density decreased (mainly *Acanthurus nigrofuscus* but also *C. striatus*) and ciguatera cases declined, the latter from a peak in 2004 [207]. Before the peak in ciguatera cases, there were increases in turf algae cover but unfortunately there was no information on the abundance of surgeonfish between 1999 and 2006, that is, the years prior to the sudden increase in ciguatera cases in 2004. So, although Rongo and van Woesik [207] concluded that high densities of *C. striatus* were a good predictor of ciguatera cases, their data showing concurrent decreases in ciguatera cases with turf algae cover may be consistent with our feedback hypothesis for production of greater ciguatoxin loads in herbivores under reduced grazing pressure.

To date, long-term monitoring of herbivorous parrotfish and surgeonfish indicate that abundances across the Great Barrier Reef have generally remained stable over time, with some cross-shelf variability [209]. In part, this is likely because herbivorous fishes are not generally harvested by recreational or commercial fishers on the Great Barrier Reef [183,184]. The zoning of the Great Barrier Reef into areas that are permanently open or closed to fishing has therefore created an on-going experiment on the effect of top-down control of herbivory by predatory fish, especially the mostly meso-predators targeted by fishers such as Serranid, Lethrinid and Lutjanid fishes. As expected, the meso-predatory fish targeted by fishers have much greater abundance on reefs closed to fishing compared to those open to fishing [271,272], but this has not led to increased herbivore abundance on fished reefs or an increase in turf algae [272,273,274,275]. On the Great Barrier Reef, herbivore abundance appears to be weakly controlled by predators [273,274,276], at least at the current level of fish harvesting. Similar results were found by Mellin et al. [277] in an analysis of the effects of disturbance from storms, disease, crown-of-thorns starfish and coral bleaching on fished and un-fished reefs of the Great Barrier Reef, with the exception that all four types of disturbance led to increases in turf algae. This resilience of herbivore fish populations to predatory control may keep herbivore populations high and help prevent the production and transfer of ciguatoxins to predatory fish on the Great Barrier Reef, if the feedback conceptual model (Figure 8) for production of toxic herbivores is correct. In the only study to date of ciguatoxins from surgeonfish from the Great Barrier Reef, Lewis et al. [175] found only low levels of ciguatoxins from *C. striatus* collected from John Brewer and Davies Reefs off Townsville.

The coral reef fisheries of many island nations are thought to be overfished or being unsustainably fished [278]. If the feedback conceptual model for development of ciguatoxic fish (Figure 8) is correct, then a sudden loss of herbivores could be a process that concentrates the trophic transfers of ciguatoxins into the remaining smaller fish populations (herbivores and then carnivores), possibly leading to local increases in ciguatera if there are simultaneous increases in ciguatoxin-producing *Gambierdiscus*/*Fukuyoa* species. The loss of herbivore populations may produce phase shifts in ecosystems from coral to macroalgal dominated states [279,280], although the top-down control of macroalgae by parrotfish has been questioned [281]. Ciguatera research has tended to interpret a shift to macroalgae dominated reefs as an increase in the amount of substrate that supports growth of benthic dinoflagellate populations. The underlying assumption to this interpretation is that lower amounts of macroalgal foliage is limiting benthic dinoflagellate populations and therefore the production and transfer of ciguatoxins into fishes consumed by people. However, it may be the change in ecosystem functioning brought about by the reduction of herbivory that leads to increases in ciguatera, rather than the change to a macroalgal dominated ecosystem per se.

We have hypothesized that *Gambierdiscus* or *Fukuyoa* growing on turf algae are the major contributor to the flow of ciguatoxins into food chains on the Great Barrier Reef because:

- *Gambierdiscus* are common epiphytes of turf algae on the Great Barrier Reef;
- Turf algae are ubiquitous on reefs; and
- Turf algae have high rates of productivity which support high grazing pressure by surgeonfishes on the Great Barrier Reef.

However, most sampling of benthic dinoflagellates around the world to-date, including ours (MJH, RJL), has focused on macroalgal substrates. Often this is because it is physically easier to sample macroalgae than turf algae, and because of the difficulty in interpreting and comparing dinoflagellate populations from turf algae. However, the assumption underlying most sampling of *Gambierdiscus* and *Fukuyoa* is that the population numbers will relate to the risk of ciguatera (although this is not always explicitly stated). Researchers often focus on macroalgae that produce the highest density of benthic dinoflagellates, without considering the likelihood of those macroalgal species being consumed by herbivores and becoming part of a ciguateric food chain.

Some macroalgae species on the Great Barrier Reef are consumed by herbivorous fishes but often consumption is locally dominated by a single fish species which varies between locations [282,283,284]. At Lizard Island on the Great Barrier Reef, the dominant herbivore of macroalgae (the unicornfish *Naso unicornis*), was the eighth most abundant of the local herbivore species by numbers and second most abundant by biomass [284]. This indicates that the fish controlling macroalgae abundance may not be obvious based upon simple observations of the local fish community when sampling macroalgae for benthic dinoflagellates. Studies that identify functional processes within local reef ecosystems, e.g., [284,285], may help ciguatera studies target the appropriate fish species to understand the transfer of ciguatoxins along localized food chains. This is especially so for the Great Barrier Reef, where fishers mostly target higher trophic level fishes [184,286,287] so only carnivorous species from this ecosystem tend to cause ciguatera.

Many herbivorous fishes, including surgeonfishes void their faeces over the reef with much of the algal material, including turf algae, remaining structurally intact and capable of growth [288]. *Ctenochaetus* and other surgeonfish feeding over turf algae likely consume a considerable amount of this faecal material [257], which if it contains undigested or damaged *Gambierdiscus*, could be a re-circulation mechanism for concentrating ciguatoxins into surgeonfish. Defecation by surgeonfishes moves sediments across reefs [289] and may also be a mechanism for dispersing benthic dinoflagellates, but this has not been studied.

Small benthic carnivorous fishes, and parrotfish, consume large numbers of crustaceans and other invertebrates living within turf algae [290]. Nothing is known about the potential involvement of these invertebrates in ciguateric food chains on the Great Barrier Reef. However, given our hypothesis for the transfer of ciguatoxins through invertebrates to blotched-javelin fishes in Platypus Bay, it is worthy of study. This could include examining the role for the transfer of ciguatoxins from invertebrates into some 17 families of cryptobenthic reef fishes [291]. It was recently suggested that cryptobenthic fishes account for almost 60% of the fish biomass consumed by reef predators on the Great Barrier Reef [292]. To date, ciguatera research has focussed on the trophic transfer of ciguatoxin to carnivorous fishes from larger herbivorous fishes such as surgeonfishes, while most of the marine food chain on coral reefs may be driven through relatively small fish that grow fast but suffer extreme mortality, such as gobies, blennies, and cardinal fish [292].

### 5.3. The Food Chain Links Leading to Ciguatera along the Great Barrier Reef, Trophic Levels 3 and 4

Ciguatera from the Great Barrier Reef is mostly caused by demersal, meso-predatory fish species such as coral trouts (*Plectropomus* and *Variola* spp.), emperors/tropical snappers (*Lethrinus* and *Lutjanus* spp.) and cods (*Epinephelus* spp.) [5,293], all of which are targeted by commercial and recreational line fishers [184,286,287]. The adults of many of these demersal predatory reef fishes are thought to have relatively high reef fidelity with little range movement on the Great Barrier Reef [294,295], in contrast with Spanish mackerel which are pelagic and often travel large distances. Stable isotope analysis suggests that there is little dietary niche overlap of Spanish mackerel with demersal reef fishes such as coral trout on the Great Barrier Reef [146,296]. If dietary overlap is the exception, then possibly different food chains are responsible for the transfer of ciguatoxins from the causative benthic dinoflagellates *Gambierdiscus* and *Fukuyoa* spp., to demersal and pelagic fish predators. Alternatively, it is possible that the intoxicating effects of ciguatoxins and/or water-soluble toxins such as maitotoxins on some herbivorous fishes might produce opportunistic feeding for both demersal and pelagic reef predators on the Great Barrier Reef, creating space for dietary niche overlap and toxin transfer.

Coral trout is the common name for a species complex of demersal Serranid fishes (groupers) on the Great Barrier Reef, made up mostly of the common coral trout (*Plectropomus leopardus*), bar-cheek coral trout (*P. maculatus*), blue-spot coral trout (*P. laevis*), passionfruit coral trout (*P. areolatus*), vermicular cod (*P. oligacanthus*), yellow-edge coronation trout (*Variola louti*) and white-edge coronation trout (*V. albimarginata*) [297]. Of these, the common coral trout (*P. leopardus*) is the main target species of the commercial coral reef, fin-fish fishery [286]. Coral trout are considered premier and high-priced food fishes in Australia, in contrast to Spanish mackerel which is only a medium-priced fish (Sydney Fish Market website). On the east coast of Australia, a far greater weight of coral trout is caught and sold commercially than of Spanish mackerel with most fish caught commercially after about 1996 exported live to overseas markets in Asia [298]; although this was interrupted in 2020 through export restrictions on live species caused in-part by the human coronavirus COVID-19 disease pandemic. On the Great Barrier Reef, most of the commercial catch of coral trout (*P. leopardus*) comes from mid- and outer-shelf reefs whereas recreational catches of *P. maculatus* dominate in-shore reefs [286]. In contrast with Spanish mackerel, where catches are almost equal between commercial and recreational sectors [83], the commercial catch dominates the coral trout harvest [286,299]. *Plectropomus leopardus* form a single stock on the east coast of Queensland and are protogynous hermaphrodites, beginning life as a female, with many later changing to male [298]. Stocks are considered sustainably fished on the Great Barrier Reef [286] with the population level in 2020 about 59% of the unfished spawning biomass [299].

**Figure 9:**
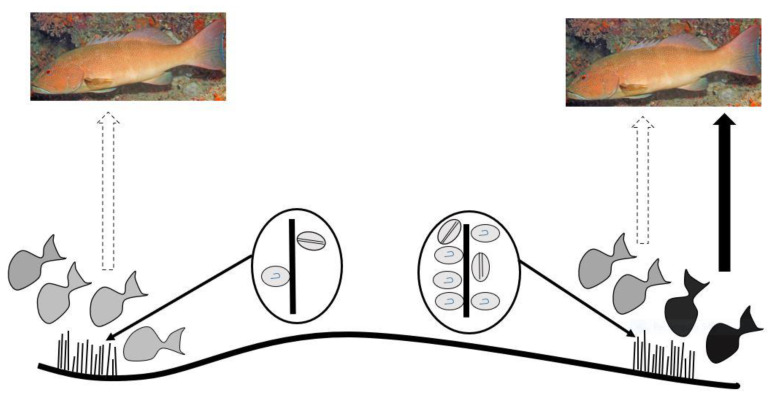
Conceptual model for hypothesised transfer of ciguatoxins from ciguatoxin-producing *Gambierdiscus* or *Fukuyoa* to herbivorous fish (such as surgeonfish) and then to predatory reef fish such as coral trout (*Plectropomus* spp.), in an ecosystem where herbivorous fish populations are mostly unimpacted (such as the Great Barrier Reef). On the left, surgeonfish graze on turf algae with low populations of benthic dinoflagellate populations (as indicated by low numbers of dinoflagellates in oval frame). This limits the amount of ciguatoxin being transferred to the many herbivorous fishes grazing on the turf community, and individual fish are lowly or non-toxic (indicated by lighter shading of surgeonfish). There is little transfer of ciguatoxin to coral trout because these lowly-toxic surgeonfish make up a small proportion of their diet (broken arrow). On the right, the occasional bloom of benthic dinoflagellates leads to a higher ciguatoxin load being accumulated by some surgeonfish (indicated by the darker shading). The intoxicating effects of the dinoflagellate toxins on these surgeonfish renders them more likely to be preyed upon by opportunistic predators such as coral trout leading to toxin transfer. (Coral trout *P. leopardus* image: Graham Edgar, www.reeflifesurvey.com (accessed on 20 July 2021), Creative Commons by Attribution licence for non-commercial use).

We have presumed that predatory demersal fish such as coral trout accumulate ciguatoxins by feeding on ciguatoxic herbivores such as surgeonfishes [70], but there may be alternate food chains for accumulation of ciguatoxins, such as through cryptobenthic fish species. Coral trout are high trophic level predators that eat mostly fish from a wide range of prey families plus a small proportion of invertebrates [300,301]. Gut analysis of speared *P. leopardus* from the Great Barrier Reef indicated that herbivores (parrotfishes, surgeonfishes, and rabbitfishes) make up <10% of prey numbers, with surgeonfishes <3% [301]. Recent studies using a combination of gut analysis, DNA metabarcoding and stable isotope analysis suggest that many prey species of coral trout may not be detected by gut analysis alone. However, this study also confirmed that herbivores make up only a small component of their diet, and relative to their abundance, surgeonfishes appear to be not selected as prey [302]. Instead, prey is dominated by planktivorous fishes (fusiliers, Caesionidae and damselfishes, Pomacentridae) and other carnivores [302]. Surgeonfishes such as *Ctenochaetus* and *Acanthurus* appear to be only a small part of the diet of both *P. leopardus* and *P. maculatus*, with *P. maculatus* feeding on more benthic prey than *P. leopardus* [296,302]. How do coral trout become poisonous based upon a predominant diet of planktivorous fishes? Their diet is not thought to change seasonally [303]. Coral trout are opportunistic generalist carnivores, so if surgeonfishes were intoxicated by ciguatoxins and/or maitotoxins when feeding on high populations of *Gambierdiscus* or *Fukuyoa*, then this could provide a mechanism for temporary diet switching to surgeonfishes by coral trout. Two Caribbean surgeonfishes (*Acanthurus bahianus* and *A. chirurgus*) fed a gel diet containing a *Gambierdiscus* species became disorientated and lost equilibrium [262], behaviour that in the wild would greatly increase their chance of predation. However, Magnelia et al. [262] found that surgeonfish could acclimate to feeding on a fixed dose of *Gambierdiscus* suggesting that the intoxicating effects, and risk of opportunistic predation from coral trout and many other carnivorous fish species, may be greatest after they feed on a sudden population increase (bloom) of *Gambierdiscus*/*Fukuyoa*. This may be a mechanism by which occasional blooms of ciguatoxin-producing benthic dinoflagellates lead to the production of ciguatoxic predatory reef fish in ecosystems such as the Great Barrier Reef where herbivorous fish populations are largely unaffected by fishing pressure (Figure 9). Marshell and Mumby [263] reported a considerable range in the rate of turnover of turf algae by surgeonfish at Heron Island (4–25 days). Slower turnover rates may allow time for benthic dinoflagellates to occasionally bloom in patches across the Great Barrier Reef, producing the occasional (stochastic) opportunity for ciguatoxins to flow through herbivores/detritovores into meso-predatory fishes such as coral trout. Alternatively, the patchy nature of surgeonfish grazing [304], may allow “islands” of un-grazed turf for benthic dinoflagellates to proliferate for a time.

If benthic dinoflagellates growing on macroalgae are a source of ciguatoxins on the Great Barrier Reef that poison people, they need to be consumed by herbivores that are preyed upon by carnivorous fish eaten by people, such as Serranid, Lethrinid or Lutjanid meso-predatory fishes. *Naso* spp. (unicornfishes) are surgeonfishes that feed on macroalgae, so may be one such intermediate, with *N. unicornis* being the dominant grazer of macroalgae at Lizard Island on the Great Barrier Reef [284]. However, similar to other surgeonfishes, unicornfishes appear not be selected as prey by coral trout species [296,300,302]. Opportunistic predation on intoxicated herbivores could facilitate toxin transfer; however, Clausing et al. [102] reported that the unicornfish, *N. brevirostris*, did not display any abnormal behaviour when fed a gel diet containing *G. polynesiensis*. Possibly there are species-specific differences in the response of herbivores to the various suite of toxins produced by *Gambierdiscus*/*Fukuyoa*. Otherwise, even if grazers of macroalgae accumulate ciguatoxins, if they are not preyed upon by carnivorous fishes, their toxin load is unlikely to become part of the food chain leading to ciguatera from the Great Barrier Reef.

Matley et al. [302] found that damselfishes (Pomacentridae) make up a large proportion of the prey of coral trout which includes species such as *Stegastes nigricans* that “farm” algal turfs. The turf algae within the Great Barrier Reef territories of these farming damselfishes have longer lengths and higher detritus and lower sediment loads than those outside [305], so may be favourable habitats for growth of benthic dinoflagellates, although we are not aware of any studies. Damselfishes can consume the detritus fraction directly as well as invertebrates within the algal matrix [306], so this could be a mechanism for toxin transfer from epiphytic dinoflagellates. Tebbett et al. [305] suggest that the farmed turfs of damselfish may also incentivise the raiding of them by schools of other herbivorous/detritivorous fishes, providing multiple mechanisms for the transfer of ciguatoxins if damselfish territories offer scope for growth of *Gambierdiscus*/*Fukuyoa*. In regions where herbivores are fished, biomass and abundance of territorial algal farming damselfishes increases [307]. Coral trout preying upon other carnivorous fishes that have accumulated ciguatoxins such as Lutjanids and Lethrinids [302], might be a secondary route for them to become ciguatoxic.

### 5.4. Summarizing the Production and Food Chain Transfer of Ciguatoxins from the Great Barrier Reef

**Figure 10:**
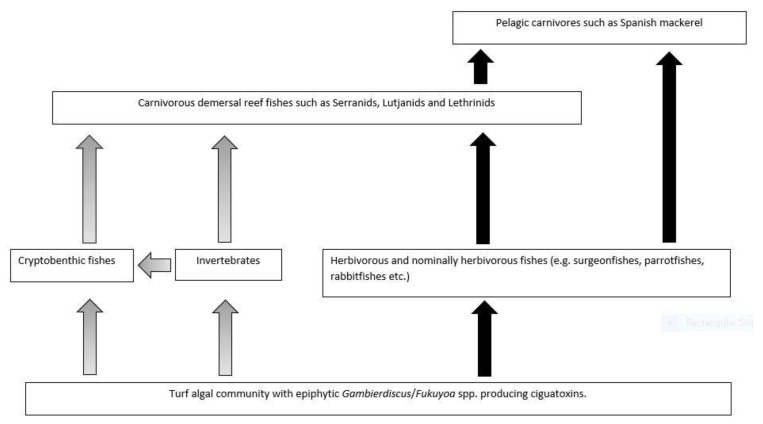
Conceptual model for food chain transfer (arrows) of ciguatoxins on the Great Barrier Reef. Solid arrows indicate transfer of ciguatoxins consistent with the general paradigm for the accumulation of ciguatoxins (Randall 1958). Shaded arrows indicate possible additional linkages.

The Great Barrier Reef marine park covers an area >340,000 km^2^ with cross-shelf and latitudinal differences in ecosystem structure. There are no local-scale studies of trophic transfers of ciguatoxins to extrapolate generalizations of the food chain links for communities across these large-scale differences. At present, we can only base our conceptual model (Figure 10) on the knowledge that *Gambierdiscus* is a common component of turf and macroalgae communities across the Great Barrier Reef, together with recent research on the biology and fisheries of Great Barrier Reef fishes. We believe that *Gambierdiscus* or *Fukuyoa* species growing on turf algae are likely the main source of ciguatoxins entering marine food chains on the Great Barrier Reef to cause ciguatera, but this remains to be tested. There are at least three possible food chains for the transfer of ciguatoxins from *Gambierdiscus*/*Fukuyoa* through invertebrates, cryptobenthic fish or larger herbivorous fish species to demersal predators such as coral trout (Figure 10). We suggest that the abundance of surgeonfish that feed on turf algae on the Great Barrier Reef is a feedback mechanism controlling the flow of ciguatoxins through the marine food chain and suggest that this hypothesis could be explored through cage experiments. The intoxicating effects of ciguatoxins and/or water-soluble toxins on herbivores, may be greatest after feeding on a sudden population increase such as a *Gambierdiscus*/*Fukuyoa* bloom, which likely increases the risk of opportunistic predation on them. This may be a mechanism by which occasional blooms of ciguatoxin-producing *Gambierdiscus*/*Fukuyoa* lead to the production of ciguatoxic predatory reef fish in ecosystems such as the Great Barrier Reef, where herbivorous fish populations are largely unaffected by fishing pressure. This likely concentrates ciguatoxins into the human food chain.

However, as with Platypus Bay, some of the same fundamental questions remain:

- What are the *Gambierdiscus*/*Fukuyoa* species that produce ciguatoxins on the Great Barrier Reef?
- What is the profile of ciguatoxins produced by these species?
- What ciguatoxins are transferred/transformed along the food chain that leads to the ciguatoxin profile found in demersal reef fish such as coral trout?
- Which of the potential food chains (Figure 10) operate to produce demersal ciguatoxic fish such as coral trout?

## 6. Model for Dilution of Ciguatoxins in the Flesh of the Common Coral Trout (*P. leopardus*) through Growth

We constructed growth models to explore the potential for dilution of ciguatoxin concentrations (5.0, 1.0, 0.1 and 0.03 µg/kg P-CTX-1 equivalents) in the flesh of the common coral trout to below the U.S. FDA threshold concentration of 0.01 µg/kg P-CTX-1 equivalents. Our modelling is based upon the same assumptions and limitations we outlined for Spanish mackerel with regard to; the homogeneity of ciguatoxins in the flesh, the ratio of flesh (fillet) weight to whole fish weight, any effect of ciguatoxins on growth, and any negative or positive bias for catchability as coral trout are mostly caught by line fishers using baits or lures.

The minimum legal size for taking all coral trout species in Queensland is 38 cm total length, except for the blue-spot trout (*P. laevis*) which has minimum size of 50 cm and a maximum size of 80 cm. Common and bar-cheek coral trout are long-lived species with common coral trout reaching 38 cm total length between 2–3 years of age, and most commercially harvested fish being 3–5 years old and averaging 1.58 kg [286]. However, the large variation in growth between individual fishes [155], adds considerable uncertainty to any estimation of the dilution of toxicity from growth of *P. leopardus*. It has been suggested that only about 5% of the population eventually reach 10-years or older [308].

**Figure 11:**
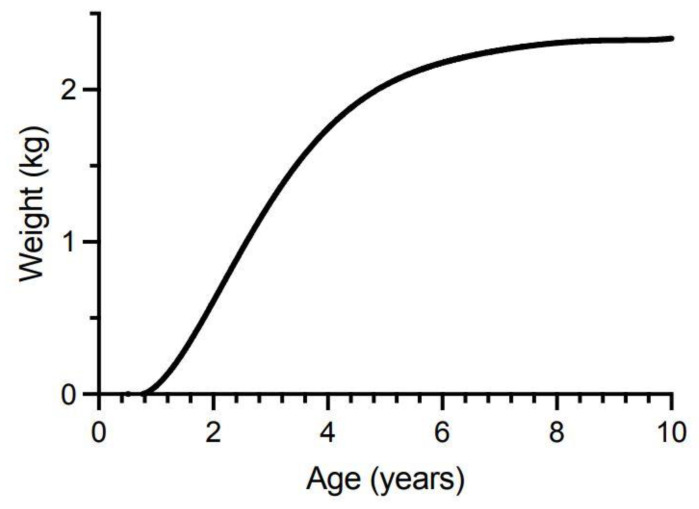
Growth curve (weight-at-age) for common coral trout from the east coast of Australia (data provided by the Queensland Department of Agriculture and Fisheries from Campbell et al., [298]).

**Figure 12:**
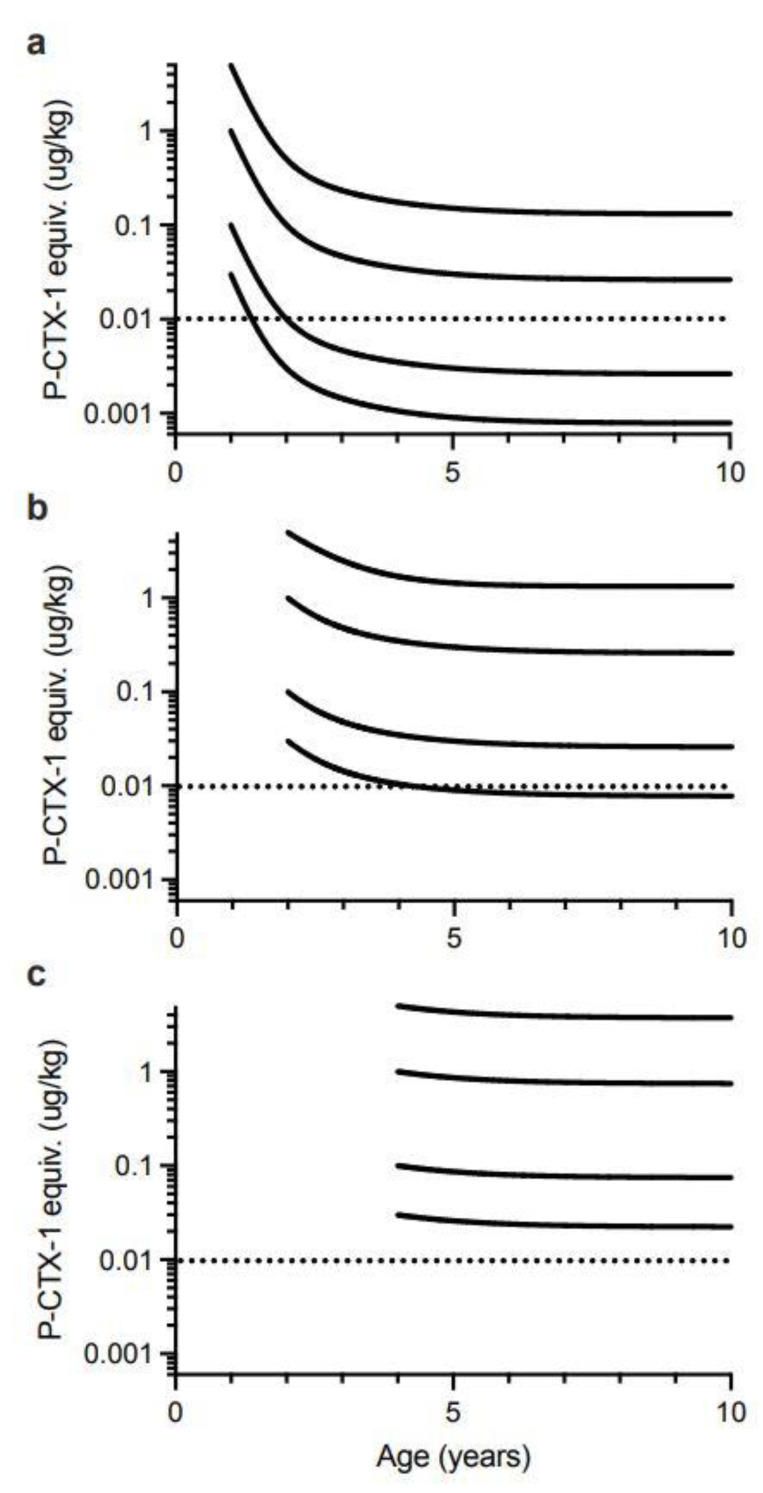
Modelled dilution of 5.0, 1.0, 0.1 and 0.03 µg/kg P-CTX-1 equivalents from the flesh of common coral trout (*P. leopardus*) by somatic growth, for fish contaminated with ciguatoxins at; (**a**) 1 year of age, (**b**) 2 years of age and (**c**) 4 years of age. Dotted line = USFDA precautionary action concentration of 0.01 µg/kg of P-CTX-1 equivalents.

The annual weight-at-age data to construct the growth curve for common coral trout (Figure 11) was supplied by the Queensland Department of Agriculture and Fisheries from data published in Campbell et al. [298] (their Figure 3). We used this data to estimate the annual rate of dilution of ciguatoxin concentrations by somatic growth from 1-, 2- and 4-year-old fish (Figure 12), as we did for Spanish mackerel. The model assumes that the ciguatoxin concentration in muscle decreases in proportion to the relative increase in mass from somatic growth. We did not include 0.5-year-old coral trout as young ages are poorly represented by the weight-at-age data (Figure 11).

Somatic growth can reduce the ciguatoxin concentrations contaminating young (≤1-year of age) coral trout by ~10-fold (Figure 12), before the fish reaches the legal size for harvesting at 2–3 years of age [286]. As with Spanish mackerel, this would likely reduce the severity of the disease if the fish did not accumulate additional toxin before it was harvested and eaten. However, somatic growth cannot reduce the ciguatoxin concentration of a 1-year-old fish contaminated with 1.0 µg/kg P-CTX-1 equivalents to below the U.S. FDA threshold of 0.01 µg/kg over a 10-year modelled lifetime (Figure 12). For fish >2years-old, somatic growth is unlikely to reduce the frequency of toxic fish except for those contaminated with very low (e.g., 0.03 µg/kg P-CTX-1 equivalents) concentrations of ciguatoxins (Figure 12), especially as most fish are harvested between 3–5 years of age [286]. Therefore, as with Spanish mackerel, somatic growth on its own is unlikely to significantly reduce the toxicity of ciguateric coral trout, especially if the fish accumulates the toxin after 1–2 years of age. We have not explored the possible depuration of ciguatoxins from coral trout as we have no evidence to support this hypothesis, although we think it likely.

Other reef fish species such as red bass (*Lutjanus bohar*) and the lined-bristletooth (*Ctenochaetus striatus*) are frequently implicated in ciguatera throughout the Pacific, and the former is a no-take fish in Queensland because of the risk of ciguatera. Red bass is often found on the mid- and outer-shelf of the Great Barrier Reef [180], as is *C. striatus* [255]. Both are long-lived species and very-slow growing after an initial period of rapid growth, with red bass living up to 43-years [309] and *C. striatus* between 30–45 years [310]. Based upon our models for the dilution of ciguatoxins by somatic growth in Spanish mackerel and common coral trout, growth on its own is unlikely to significantly reduce toxicity in red bass or lined- bristletooth surgeonfish, except if they accumulate toxins at a young age.

## 7. Comparative Risk of Ciguatera Estimated from Catches of Spanish Mackerel and Coral Trout (*Plectropomus* spp.) along the East Coast of Australia

Based upon the detection of a single toxic sample from the testing of the flesh of 71 Spanish mackerel caught in northern New South Wales waters in 2015, Kohli et al. [29] estimated the frequency of ciguatoxin contamination at 1.4%. This was the first study to estimate incidence rates for any fish from Australia. However, 1.4% seems high if this translated into fishes with the potential to cause ciguatera, given how heavily the stock is targeted by both commercial and recreational fishers. The commercial harvest of Spanish mackerel from the east coast of Queensland in 2015 was 299 tonnes, with about 46 tonnes taken from southern Queensland [141]. The commercial catch from northern New South Wales waters ranges between 3 and 40 tonnes with an average of 15 tonnes since 2000 [83]. The combined east coast Queensland and New South Wales annual recreational catch since 2004 is estimated to be between 250–300 tonnes with the New South Wales recreational catch being ~13% of the recreational harvest from Queensland waters [83]. If we use the lower range of this catch (250 tonnes) to estimate the New South Wales recreational catch, then 13% of 250 tonnes = 32.5 tonnes. Spanish mackerel caught in New South Wales tend to be larger fish [79], so it would not be appropriate to use an average fish size of 7.7 kg [83] to estimate population numbers from catch weight. The 71 New South Wales fish assayed by Kohli et al. [29] had a mean weight (± 1 standard deviation) of 13.4 ± 3.9 kg (range 4.5–23.6 kg). Assuming a combined commercial (15 tonnes) and recreational catch (32 tonnes) of Spanish mackerel in New South Wales waters of 47 tonnes, with an average fish size of 13.4 kg, would suggest the capture of ~3500 fish. However, the Sydney Fish Market refuses to accept fish above 10 kg because of the risk of ciguatera so commercial fishers may discard many of these larger fish. If only a recreational harvest of about 32 tonnes was considered then this would equate to about 2350 fish, with recreational fishers possibly less likely to discard large fish. This is a conservative figure because the estimate for the annual recreational catch of Spanish mackerel in New South Wales waters during financial years 2010–2011 and 2014–2015 by O’Neill et al. [83] are more than double the above at ~5000 fish each year.

An incidence rate of 1.4% for ciguatoxic Spanish mackerel [29] would equate to 33 toxic fish per year from 2350 recreationally caught fish, or 70 toxic fish caught from 5000 fish. Even small, legal-sized Spanish mackerel can be processed into many meals, so the number of cases resulting from 33 toxic fish in one year would be expected to be a multiple of this number depending upon how many people ate the first meal from the toxic fish, i.e., before the fish was recognized as being poisonous. Most likely this would be at least two people within the family and friends of recreational fishers, but the multiplier could likely be much higher for a commercially sold fish. For example, more than 30 poisoning cases were attributed to a single 21 kg commercial consignment of Spanish mackerel [311]. In either case, 33 toxic Spanish mackerel could be expected to create >50 ciguatera poisonings per year in New South Wales alone. Ciguatera is an under-reported disease in Australia [293,312], but we think such a high incidence rate from Spanish mackerel unlikely given the media interest generated by the 9 outbreaks that poisoned 37 individuals between 2014 and 2017 in New South Wales [14]. Five of these outbreaks were from Spanish mackerel caught in northern New South Wales waters in 2014 (2 fish), 2015 (1 fish) and 2016 (2 fish). In total, they poisoned 24 individuals [14].

The commercial catch of 46 tonnes of Spanish mackerel from southern Queensland in 2015 equates to ~6200 fish based upon an average weight of 7.4 kg (mean weight of Spanish mackerel caught by commercial fishers in southern Queensland during the 2014–2015 financial year; MJH personal communication from Queensland Department of Agriculture and Fisheries). Applying an incidence rate of 1.4% [29] would suggest ~86 toxic Spanish mackerel entering the human food chain during 2015 from commercial sources alone. Ciguatera is a notifiable disease in Queensland but only 11 ciguatera poisonings were recorded for 2015 from the entire State (Queensland Health, Notifiable condition reports: https://www.health.qld.gov.au/clinical-practice/guidelines-procedures/diseases-infection/surveillance/reports/notifiable/weekly (accessed on 26 August 2019 and 20 July 2021)). The fish species causing these 11 poisonings are not available on the public database but are unlikely to have all been due to Spanish mackerel, and the number of fish causing these poisonings is likely to be less than the number of people poisoned. It is not possible to know the true incidence rate of ciguatoxic fishes without a better estimate for the actual number of ciguatera cases that occur, given that many are thought to go un-reported to authorities. However, based upon the analysis above for 2015, we suggest that the true incidence of ciguatoxic Spanish mackerel in southern Queensland and northern New South Wales is likely much less than the 1.4% suggested by Kohli et al. [29].

Campbell et al. [298] assessed the current annual Queensland harvest of common coral trout (*P. leopardus*) by commercial fishers at ~829 tonnes and ~171 tonnes by recreational fishers. As the average weight of legally caught common coral trout is 1.58 kg [286], this equates to >634,000 fish harvested annually from the Great Barrier Reef. This compares with recent estimates for the combined annual commercial and recreational harvest of Spanish mackerel from the east coast of Queensland and New South Wales at ~600–700 tonnes [83]. Using a conservative average fish size of 7.7 kg for the combined catch [83], this equates to between 77,000 and 91,000 Spanish mackerel. However, since 1996, most commercially caught common coral trout are exported live to Asia and we have no information of any ciguatera cases from these fish; although, between 2004 and 2013, *P. leopardus* and red bass (*Lutjanus bohar*) caused most ciguatera cases in Hong Kong [313].

If just the recreational component (171 tonnes; [298]) of the Queensland catch of common coral trout (*P. leopardus*) is compared with the total catch of Spanish mackerel, it suggests that >108,000 common coral trout are eaten annually from the east coast of Australia compared with a conservative estimate of 77,000–91,000 Spanish mackerel. However, most of the recreational catch of coral trout from the Great Barrier Reef is bar-cheek coral trout (*P. maculatus*) [286], so more than twice the number of coral trout of all species is likely consumed on the east coast of Australia compared to Spanish mackerel. If the risk of ciguatera from Spanish mackerel and demersal reef fish were the same, we would expect more than twice the number of outbreaks from the east coast of Australia caused by coral trout than Spanish mackerel.

Reconstructed historical catches of Spanish mackerel and common coral trout from the east coast of Australia for 1986, the year before the ban on taking Spanish mackerel from Platypus Bay and before the development of the live export market for coral trout, indicate total catches of about 680 tonnes (>88,000 fish) and 1200 tonnes (>759,000 fish), respectively [83,298], suggesting that at times there was at least an order of magnitude more coral trout (of all *Plectropomus* and *Variola* species) consumed on the east coast of Australia than Spanish mackerel. This does not consider the many other demersal species caught by commercial and recreational fishers from the Great Barrier Reef that can cause ciguatera. For example, between 1990 and 2018, an average of 524 ± 243 tonnes of red-throat emperor (*Lethrinus miniatus*) were harvested annually by commercial fishers from the Great Barrier Reef [141]. At an average weight of 1.17 kg [314] this suggests that >448,000 of these reef fish are eaten annually in Australia from the commercial food chain; not including the considerable annual recreational catch of this species which was estimated at 65,000 fish in a 2010 survey [297].

We do not know the true incidence of ciguatera for any fish species from Australia, but Gillespie et al. [5] reported 30 outbreaks of ciguatera caused by Spanish mackerel and another 51 by unknown mackerel species between 1965 and 1984 (before the ban on harvesting Spanish mackerel from Platypus Bay came in force in 1987). If just half of the unknown outbreaks were Spanish mackerel, this would equate to ~55 outbreaks from Spanish mackerel whereas over the same period there were only 18 outbreaks from coral trout or 54 from all non-pelagic fish species [5]. This suggests that the probability of ciguatoxins being accumulated is much greater for a Spanish mackerel on the east coast Queensland than a demersal reef fish, but the considerable underreporting of ciguatera makes it difficult to be certain. Unfortunately, there are no recent studies linking fish species with ciguatera cases in Queensland to make better comparisons using fisheries data. If this were available, it would be possible to use fishery models to estimate the ciguatera risk from the catch of populations of individual fish species from the Great Barrier Reef and the east coast of Queensland. For some species, this could extend to estimating the risk from the entire population of legal-sized fish on the Great Barrier Reef as Leigh et al. [286] were able to estimate the number of legal-sized common coral trout in fishable zones of the Great Barrier Reef in the 1980′s at >5.3 million fish, with another >2.1 million fish in no-fishing zones.

A general paradigm of ciguatera research is that larger fish carry a higher risk of ciguatera because the toxins bio-accumulate through the marine food web [70], leading to general advice to be cautious about eating large reef fish [5]. Early research suggested that red bass (*Lutjanus bohar*) could remain toxic for up to 30 months when fed on a non-toxic diet [152]. It is therefore understandable that the Sydney fish market’s response to previous cases of ciguatera from large Spanish mackerel is to not accept any fish over 10 kg, which corresponds to a 3- to 4-year-old female fish or a 5- to 7-year-old male fish (Figure 5). However, the evidence for a relationship between fish size and toxicity is contradictory. Early research by Hessel et al. [315] and Banner et al. [316] in the Line Islands in the Pacific found an increasing frequency of toxic red bass with fish size, as well as an increasing toxicity with size. Vernoux [317] found toxicity correlated with size for one species of trevally (*Caranx*) in the Caribbean, but not another. Chan et al. [87] and Mak et al. [32] found a positive correlation between body weight and ciguatoxin concentration for moray eels from the Republic of Kiribati, whereas previously, Lewis et al. [86] had found no such relationship. Bravo et al. [318] suggested that weight was a risk factor for amberjack (*Seriola* spp.) from the Canary Islands in the Atlantic Ocean, whereas Gaboriau et al. [137] found little relationship between toxicity and reef fish size in French Polynesia (with the possible exception of red bass). All the above suggests that any relationship between fish size and ciguatera risk varies between species and their environment. In an environment with continuous or periodic input of ciguatoxins into the food chain, larger fish of the same species are likely older and could have accumulated a higher ciguatoxin burden assuming that the rate of toxin input is greater than losses/dilution through growth and depuration. For some species, the risk could also change with age if this was associated with ontogenetic changes in feeding behaviour or diet. Ciguatera in Australia is rare, and Kohli et al. [29] could not detect any relationship between size and frequency of toxic Spanish mackerel from the east coast of Queensland and New South Wales. It is possible that for many species, fish size is not a higher risk of ciguatera in Australia, but fish size carries a higher community risk because of the larger number of people that can be poisoned by a large toxic fish compared to a small one, as happened in 1994 in New South Wales, when more than 30 people were poisoned by 21 kg of Spanish mackerel caught from Queensland [311].

Large, minimally toxic fish caught by recreational fishers have an additional risk for the fisher and their family and friends, as they are more likely to eat repeat meals from the same fish, than people who eat single fish portions sold commercially through restaurants and other food outlets. While restaurant clients eating a single portion of a lowly toxic fish may avoid being poisoned, people eating repeat meals from a lowly toxic fish may become eventually succumb to poisoning [4], as was the case with a recreational spearfisher that captured a lowly toxic blue-spot coral trout off Moreton Island and only became sick after eating repeat meals (MJH personal communication).

Our modelling shows that dilution of ciguatoxin concentration through somatic growth of fishes is a slow process (Figure 6 and Figure 12), and that fish can retain toxicity for considerable time even if they depurate toxins (Figure 7 and Figures S9–S12). If Spanish mackerel can depurate ciguatoxins with a half-life of six months, it would still take more than a year for the flesh of a lowly toxic fish (0.1 µg/kg P-CTX-1 equivalents) to reach the U.S. FDA recommended safe concentration (Figure 7, Table 2). For Spanish mackerel undertaking annual migrations along the southern Queensland and northern New South Wales coasts, a year could allow for fish to re-visit the same toxic areas and accumulate an additional toxin burden.

Reducing the absolute risk of ciguatera for the fishing industry and the consumers of wild-caught tropical and temperate fishes may require the development of a rapid, sensitive, reliable, and low-cost assay, preferably one that could be used in the field or in a non-laboratory environment. Cost will be an important factor for deployment of such an assay in both poor and wealthy communities, as poorer consumers may not be able to afford the additional cost of testing individual fish or fish fillets, and richer consumers generally have choice between a range of alternate sources of animal protein. The recent development and commercialization of an ELISA that can detect a range of ciguatoxins including P-CTX-1 at 0.01 ppb [319] offers hope for the future development of portable kits for the detection of ciguateric fishes. An antibody-based approach has also been developed using an electrochemical immunosensor [320]. Alternatively, the relative long-term risk of ciguatera from different fish species along the east coast of Australia could be estimated with the use of currently available fisheries data, if better epidemiological data on ciguatera cases and the causative fishes was available.

## 8. Disturbance and the New Surface Hypothesis for Ciguatera

The Great Barrier Reef has been impacted by an increasing range of major disturbances from cyclones, crown-of-thorns starfish, catchment runoff from past and on-going development, and coral bleaching events due to increasing water temperatures from climate change [114]. Disturbance to reef environments has long been suggested to be linked with increased ciguatera in the Pacific [70,153,182,207,258,321]. In what became known as the “new surface hypothesis”, Randall [70] suggested that disturbance created new surfaces for the causative organism to colonise and proliferate (implying that space for growth is otherwise limited). Fleshy macroalgae and/or turf algae can colonise and dominate coral reefs after major disturbances [277,279,285,322,323,324], although reefs and their fish communities vary in their resilience to these ecological phase shifts [285,324,325]. After the discovery of *Gambierdiscus* [40], it was suggested that this epiphytic benthic dinoflagellate attached to, and proliferated on, algae colonizing new surfaces [193]. However, there are few direct studies that have tested this hypothesis, and over the past decade, ecologists have found that the response of reefs to any one pressure is often non-linear [326]. Kaly and Jones [327] could not find supporting evidence for the new surface hypothesis from studies on reef disturbance sites in Tuvalu. An unpublished study (Holmes and Lewis) at a small marina development site on a fringing reef at Hayman Island on the Great Barrier Reef in the 1980’s failed to detect any increase in *Gambierdiscus* populations near the development site (data archived in [186]). In the only study of the toxicity of surgeonfishes (*Ctenochaetus striatus*) from the Great Barrier Reef, Lewis et al. [175] found only low concentrations of ciguatoxins from *C. striatus* speared from John Brewer and Davies reefs. At the time, John Brewer reef had been damaged by crown-of-thorns starfish whereas Davies Reef had only been lightly impacted [175].

The impact of disturbance to coral reefs on fish populations is not clear and may vary with local conditions. Morais et al. [285] studied changes in fish populations across a mid-shelf coral reef at Lizard Island on the Great Barrier Reef that had suffered major coral degradation over 14–15 years from cyclones and coral bleaching events. These stressors led to increases in turf algae but not macroalgae, with coral loss associated with increases in total herbivorous fish biomass and productivity but with decreased turnover. There was a shift in the size structure of parrotfish, surgeonfish, and rabbitfish populations to larger bodied individuals, with an overall decline in abundance of surgeonfishes. Grazing pressure for a given biomass tends to be greatest with smaller fish sizes [328] so a population shift to larger sizes may reduce overall grazing pressure. McClure et al. [329] also found an increase in herbivore biomass for mid- and outer-shelf reefs of the Great Barrier Reef after disturbance from cyclones and coral bleaching events. However, in contrast to Morais et al. [285], they found an increased abundance of surgeonfishes, with an increase in *Acanthurus nigrofuscus* on mid-shelf reefs and increases in *Ctenochaetus striatus* and *A. lineatus* on outer-shelf reefs. Morais et al. [285] suggested that severe loss of coral can produce a more productive assemblage of reef fish herbivores but with reduced energy flow across trophic levels.

There are suggestions that increasing water temperatures through climate change could be linked with increasing ciguatera [330,331,332,333,334]. Much of this concern is about possible range expansion of benthic dinoflagellate species [335], for example southwards along the east coast of Australia into nominally temperate waters [93,253]; although, it is also possible that warmer seawater temperatures may reduce the incidence of ciguatera [97,332]. However, at the species level, some free-living dinoflagellates can have broad temperature tolerances. For example, the Paralytic Shellfish Poisoning dinoflagellate *Gymnodinium catenatum* can bloom in Tasmanian waters at >40° S latitude [336], but also grows in Singapore waters at ~1° N [337]. Higher temperatures may lead to higher latent growth rates [335] and references therein; however, this does not necessarily translate into higher cell numbers in the wild. The maximum cell densities of *Gambierdiscus* species recorded from the wild along the east coast of Australia occur quasi-seasonally but at seawater temperatures below the local annual maximum for each location [77,90,93]. However, climate driven range shifts could also increase the possibility of ciguatoxic fish species being caught by fishers from waters previously outside their range, especially highly mobile pelagic species. For example, the distribution of Spanish mackerel along the east coast of Australia has been found to be especially sensitive to the environmental effects of climate change with southward range shifts exceeding 200 km per decade [145].

Ciguatera can increase in South Pacific communities during seawater warming driven by the El Niño Southern Oscillation [330], although an earlier analysis failed to find a simple correlation [338]. Recently, Zheng et al. [339] modelled relationships between seawater surface temperature anomalies and monthly prevalence of ciguatera in French Polynesia and the Cook Islands. In both regions, there were time-lagged delays before the increase in ciguatera, although these time delays were very different between the two regions [339]. This suggests the possibility of using sea surface temperature anomalies in a risk assessment tool for ciguatera. Warming temperature (degree heating week) is routinely used to predict coral bleaching on the Great Barrier Reef, so it would be interesting to determine if there is sufficient data on ciguatera cases from the east coast of Australia to conduct modelling similar to that used by Zheng et al. [339]. However, there was an average of only ~28 ciguatera cases per year reported between 2014–2019 from Queensland (Queensland Health, notifiable conditions annual reporting: https://www.health.qld.gov.au/clinical-practice/guidelines-procedures/diseases-infection/surveillance/reports/notifiable/annual (accessed on 26 August 2019 and 20 July 2021)), compared to ~174 cases per year from the Cook Islands and ~428 per year from French Polynesia [339]. If the statistical relationship between sea surface temperature anomalies and monthly changes in ciguatera is predictive [339], it would suggest that changes in fish toxicity can occur rapidly, with increases and reductions in ciguatera cases occurring over monthly time periods. It is possible that such rapid reductions in cases may be driven through rapid toxin depuration from fish.

To date, the increasing impacts to the Great Barrier Reef have not produced any major increase in ciguatera in Australia. However, as outlined in this review, there are many links in the food chain that need to connect to produce ciguateric fishes, and the breaking of anyone could limit the bioaccumulation of ciguatoxins into the higher trophic level fishes to cause ciguatera along the east coast of Australia. Each individual link in the food chain may be described deterministically, but once linked, the chain may be chaotic.

## 9. Mitigation of Ciguatera

Lewis and Holmes [33] speculated on the potential for development of mitigation programs that can reduce the incidence of ciguatera. The apparent discrete nature of the source of ciguatera in Platypus Bay may offer the opportunity to identify key controllable factors that would underpin such strategies. Trawling or dredge removal of *Cladophora*, the substrate hosting the *Gambierdiscus* source of ciguatoxins in Platypus Bay, could be trialled for reducing ciguatera from Platypus Bay, should there be enough pressure in the future to justify such an intervention. Large scale, expensive, ecosystem interventions are being currently trialled on the Great Barrier Reef by the Great Barrier Reef Marine Park Authority and the Great Barrier Reef Foundation through manual and robotic culling of crown-of-thorns starfish. Research projects are also being funded to study the potential of even larger-scale interventions to mitigate some of the effects of climate change on the Great Barrier Reef (Great Barrier Reef Foundation; https://www.barrierreef.org/ (accessed on 20 July 2021)). The removal of *Cladophora* would be consistent with the current (2006) General Use zoning of the Great Sandy Marine Park, except for a small area along the beach which is zoned for Habitat Protection (Queensland Department of Environment and Science, https://parks.des.qld.gov.au/parks/great-sandy-marine/ (accessed on 20 July 2021)).

## Figures and Tables

**Table 1:** Catch (tonnes) of rabbitfishes (*Siganus* spp.) caught by Queensland commercial fishers from Platypus Bay between 1994 and 2018. Data derived from commercial fishers’ compulsory logbooks and retrieved from the Queensland Government’s QFish database (http://qfish.fisheries.qld.gov.au/ (accessed on 20 July 2021)). See Figure S4 for details.

Years	Rabbitfish Catch (Tonnes)
1994–1998	22.12
1999–2003	6.35
2004–2008	13.59
2009–2013	3.19
2014–2018	0.16
Total for all years	45.41

**Table 2:** Age at which 2- or 3-year-old Spanish mackerel contaminated with 0.1 or 1.0 µg/kg of P-CTX-1 equivalents are estimated to reach the U.S. FDA threshold concentration of 0.01 µg/kg P-CTX-1 equivalents, assuming a depuration half-life of 0.5, 1.0, 2.0 or 4.0 years.

Gender and Approx. Weight (kg) of 2- or 3-Year-old Ciguatoxic Spanish Mackerel	Initial [P-CTX-1] µg/kg Equivalents	Modelled Fish Age (Years) at Which P-CTX-1 Concentrations of 1.0 or 0.1 µg/kg Reach the Threshold of 0.01 µg/kg Equivalents, from Somatic Growth and Depuration (for Depuration Half-Life’s of 0.5, 1.0, 2.0 or 4.0 Years)			
		Half-Life = 0.5 Year	Half-Life = 1.0 Year	Half-Life = 2.0 Year	Half-Life = 4.0 Year
Male 2-year-old, 5.5 kg	1.0	4.9	7.5	12.4	21.6
Male 3-year-old, 7.0 kg	1.0	6.0	8.8	13.9	23.9
Female 2-year-old, 6.6 kg	1.0	4.9	7.3	11.8	20.1
Female 3-year-old, 8.7 kg	1.0	6.0	8.6	13.4	22.5
Male 2-year-old, 5.5 kg	0.1	3.4	4.6	6.6	10.0
Male 3-year-old, 7.0 kg	0.1	4.5	5.7	8.0	11.9
Female 2-year-old, 6.6 kg	0.1	3.4	4.5	6.3	9.2
Female 3-year-old, 8.7 kg	0.1	4.5	5.7	7.7	11.2

## Data Availability

Data sources are outlined in the article.

## Supplementary Materials

The following are available online at https://www.mdpi.com/article/10.3390/toxins13080515/s1, Table S1: Known species of *Gambierdiscus* and *Fukuyoa* from the Great Barrier Reef (GBR) and species found on the east coast of Australia outside the GBR, Table S2: Toxicity (~LD_50_) of chromatographic fractions from experiments conducted during the purification and characterisation of MTX-3. Figure S1: Death-time vs. dose for MTX-3, Figure S2: Elution profiles for MTX-1, -2 and -3 from a PRP-1 reverse-phase HPLC column, Figure S3: Map of Hervey Bay and Fraser Island, Figure S4: Logbook map grids for the QFish database, Figure S5: Annual combined catches of Spanish mackerel extracted from QFish 30 × 30 nm logbook grids W32, W32, V32, V33 between 1990 and 2019, Figure S6: Summed monthly catches of Spanish mackerel extracted from QFish logbook grids W32 and W33 between 1990 and 2018, and monthly numbers of ciguatoxic Spanish mackerel caught in the Hervey Bay region between 1976 and 1983, Figure S7: Map showing the central and north Queensland coasts between Proserpine and Cooktown, Figure S8: Modelled dilution of 5.0, 1.0, 0.1 and 0.03 µg/kg P-CTX-1 equivalents from Spanish mackerel flesh by somatic growth, for fish contaminated with ciguatoxins at 0.5, 1, 2, and 4 years of age, Figure S9: Modelled dilution of 5.0, 1.0, 0.1, and 0.03 µg/kg P-CTX-1 equivalents from the flesh of female and male Spanish mackerel by a combination of somatic growth and depuration half-life of 0.5 year, Figure S10: Modelled dilution of 5.0, 1.0, 0.1, and 0.03 µg/kg P-CTX-1 equivalents from the flesh of female and male Spanish mackerel by a combination of somatic growth and depuration half-life of 1.0 year, Figure S11: Modelled dilution of 5.0, 1.0, 0.1, and 0.03 µg/kg P-CTX-1 equivalents from the flesh of female and male Spanish mackerel by a combination of somatic growth and depuration half-life of 2.0 years, Figure S12: Modelled dilution of 5.0, 1.0, 0.1, and 0.03 µg/kg P-CTX-1 equivalents from the flesh of female and male Spanish mackerel by a combination of somatic growth and depuration half-life of 4.0 years, Figure S13: Map showing the position of some of the Capricorn-Bunker group of reefs and coral cays off the coast of Gladstone, Queensland.
