# Supplementary material for: Critical Review and Conceptual and Quantitative Models for the Transfer and Depuration of Ciguatoxins in Fishes
Source: Toxins (Basel). 2021 Jul 23;13(8):515. doi: 10.3390/toxins13080515 (PMC8402393; doi:10.3390/toxins13080515)
Supplement: Supplementary file 1 [file toxins-13-00515-s001.zip › toxins-1266639-supplementary.pdf]

Supplementary Materials: Critical Review and Conceptual and Quantitative Models for the Transfer and Depuration of Ciguatoxins in Fishes

Michael J. Holmes, Bill Venables and Richard J. Lewis

Table S1. Known species of *Gambierdiscus* and *Fukuyoa* from the Great Barrier Reef (GBR), and species found on the east coast of Australia outside the GBR.

Number	*Gambierdiscus* Species	Reference for Occurrence on the GBR	Reference for Occurrence outside the GBR
1	*G. toxicus*		
2	*G. belizeanus*	Murray et al. 2014. Harmful Algae 39, 242-252. (as *G.* cf. *belizeanus*)	
3	*G. australes*		
4	*G. pacificus*		
5	*G. polynesiensis*		
6	*G. caribaeus*		
7	*G. carolinianus*		
8	*G. carpenteri*	Sparrow et al. 2017 [269], Larsson et al. 2018 [107]	Larsson et al. 2018 [107]
9	*G. excentricus*		
10	*G. silvae*		
11	*G. scabrosus*		
12	*G. cheloniae*		
13	*G. balechii*		
14	*G. honu*	Rhodes et al. 2017. Harmful Algae 65, 61-70.	
15	*G. lapillus*	Kretzschmar et al. 2017 [190], Larsson et al. 2018 [107]	
16	*G. jejuensis*		
17	*G. lewisii*	Larsson et al. 2018 [107], Kretzschmar et al. 2019 [191]	
18	*G. holmesii*	Larsson et al. 2018 [107], Kretzschmar et al. 2019 [191]	
1	*F. yasumotoi*		
2	*F. ruetzleri*		
3	*F. paulensis*	Murray et al. 2014. Harmful Algae 39, 242-252.	Larsson et al. 2019 [187]
4	*Fukuyoa* sp. HK Type 1		

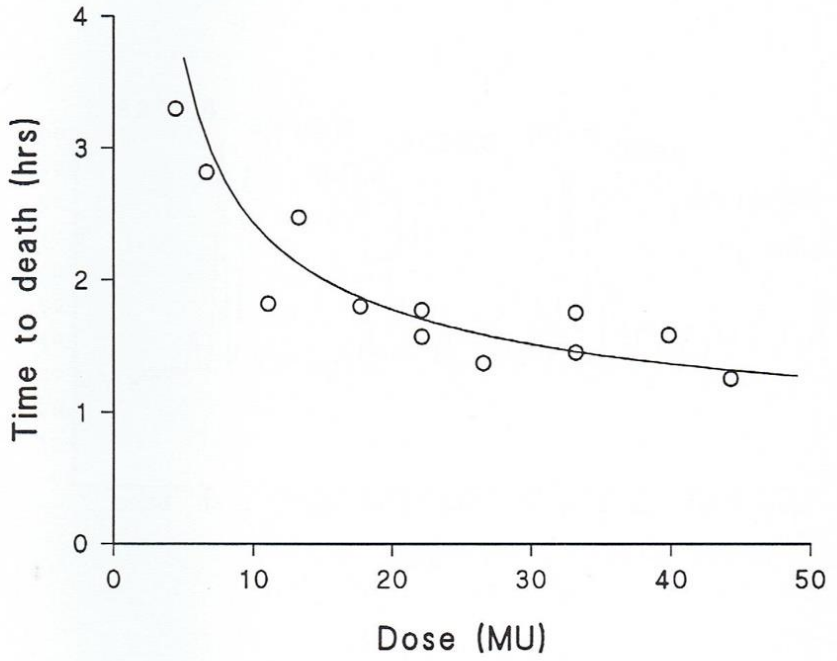

Figure S1. Death-time vs dose for MTX-3. The equation for the curve (log dose = 6.7 log(1+t^{-1})) [54] was used to quantify extracts during the purification of MTX-3 (dose is in MU, and t is time to death in hr). The derivation of such equations allowed a minimum number of mice to be sacrificed for quantification of extracts during toxin purification (animals were sacrificed under the then Australian National Health and Medical Research Council guidelines). Figure reprinted from: Holmes M.J. The Toxicity of the Benthic Dinoflagellate *Gambierdiscus toxicus* and Its Role in the Origin of Ciguatera. Ph.D. Thesis, University of Queensland, Brisbane, 1993.

Holmes et al. [52] and Holmes and Lewis [54] defined a mouse unit (MU) as the dose that caused death within 24 hr in 20 g Quackenbush strain mice injected intraperitoneally (i.p.) with up to 1 g of dry weight material per kg mouse body weight (i.e., maximum of 20 mg fraction injected into a 20 g mouse). Maitotoxin bioassays were mostly terminated after 24 hr to limit suffering with 1 MU considered as approximately equivalent to an LD_{50} dose [52]. Purified MTX-3 caused signs in mice not obviously distinguishable from those displayed by mice injected with MTX-1 or -2 [52,54] and the equation derived from the death-time vs dose curve for MTX-3 (Figure S1), was the same as that for MTX-1 [54]. Mice injected with extracts containing low doses of MTX-3 (or MTX-1 and -2) that survived for 24 hr could still shows signs of acute poisoning such as a prostrate posture with paralysis of the limbs, shallow nasal breathing, and black (cyanotic) tails. However, because the fraction did not kill within 24 hr, it would be quantified as containing < 1 MU. Murray et al. [62] injected 20 g Swiss strain mice i.p. with 0.89, 4.45, 20 and 38 mg/kg of 44-methylgambierone and none died over 24 hr. The mouse injected with 38 mg/kg showed normal behaviour for 48 hr except for low food intake. It showed hunched behaviour and laboured breathing at 56 hr and was then euthanized to avoid suffering. These signs are different to those displayed by mice intoxicated with MTX-1, -2 or -3; especially the display of "normal" behaviour for 48 hr. Murray et al. [62] interpreted the lethal (LD_{50}) dose of 44-methylgambierone at between 20 and 38 mg/kg. However, as no mice died over 24 hr, our interpretation would have been an LD_{50} >38 mg/kg. Holmes and Lewis [54] were unable to purify sufficient MTX-3 to obtain a weight and therefore to estimate an LD_{50} for pure toxin. However, the ~1000-fold greater toxicity of a partially purified extract of MTX-3 (Table S2), along with the

different signs displayed by mice injected with MTX-3 (or MTX-1 or -2), indicate that MTX-3 is not 44-methylgambierone.

Table S2. Toxicity (~LD_{50}) of chromatographic fractions from experiments conducted during the purification and characterisation of MTX-3 from the WC1/1 clone of *Gambierdiscus* [see 54]. In comparison, Murray et al. [62] reported the ~LD_{50} of pure 44-methylgambierone as between 20–38 mg/kg.

Fractionation of Extracts of the WC1/1 Clone of *Gambierdiscus*[1]	Eluate Dry Weight (mg)	~Lethal Units (Mouse Units)[2] in Fraction	~LD_{50} (μg/kg) of Fraction
Stepwise elution gradient of silica column. MeOH eluate	156	25,800	300
Stepwise elution gradient of silica column. MeOH eluate	184	80,000	115
Stepwise elution gradient of silica column. 3:7 $CHCl_3$: MeOH eluate	2978	280,000	530
C18 Sep Pak cartridge. 3:7 MeCN: H_2O eluate	250	29,000	430
C18 Sep Pak cartridge. 4:6 MeCN: H_2O eluate	16	10,000	80
C18 Sep Pak cartridge. 2:8 MeCN: H_2O eluate	233	36,000	320
C18 Sep Pak cartridge. 4:6 MeCN: H_2O eluate	28	30,000	47

[1.] Described as G. toxicus by Holmes et al. [68] when Gambierdiscus was a monotypic genus. Species now not known. [2.] 1 MU = 1 LD50 dose for a 20 g mouse over 24 h. LD50 calculated from the death time of ~20g Quackenbush strain mouse injected intraperitoneally (i.p.) with a dried fraction re-suspended in 0.5 mL of 1% Tween 60 saline (see [52] for details). Animals were sacrificed under the then Australian National Health and Medical Research Council guidelines.

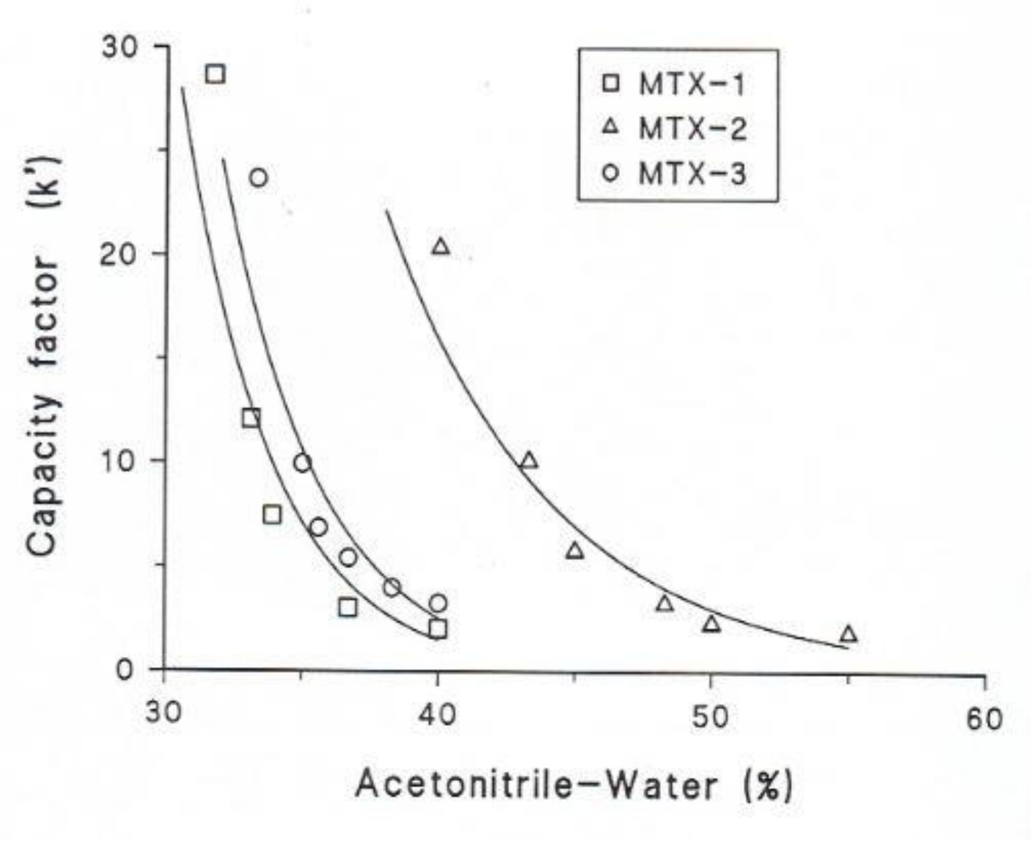

Figure S2. Elution profiles for MTX-1, -2 and -3 from a PRP-1 (Hamilton) reverse-phase HPLC column. Details of methods and statistical comparisons are reported in Holmes et al. [52] and Holmes and Lewis [54]. Capacity factor k′ = [T_R-T_0]/T_0, where T_R = retention time of the peak, and T_0 = time to non-retained peak (1.3 min). Figure reprinted from: Holmes M.J. The Toxicity of the Benthic Dinoflagellate *Gambierdiscus toxicus* and Its Role in the Origin of Ciguatera. Ph.D. Thesis, University of Queensland, Brisbane, 1993. A comparison of the elution profiles of MTX-1, -2 and -3 from a reverse-phase HPLC column reveals MTX-1 and -3 have similar elution profiles from a reverse-phase HPLC column, whereas MTX-2 is more hydrophobic (Figure S2).

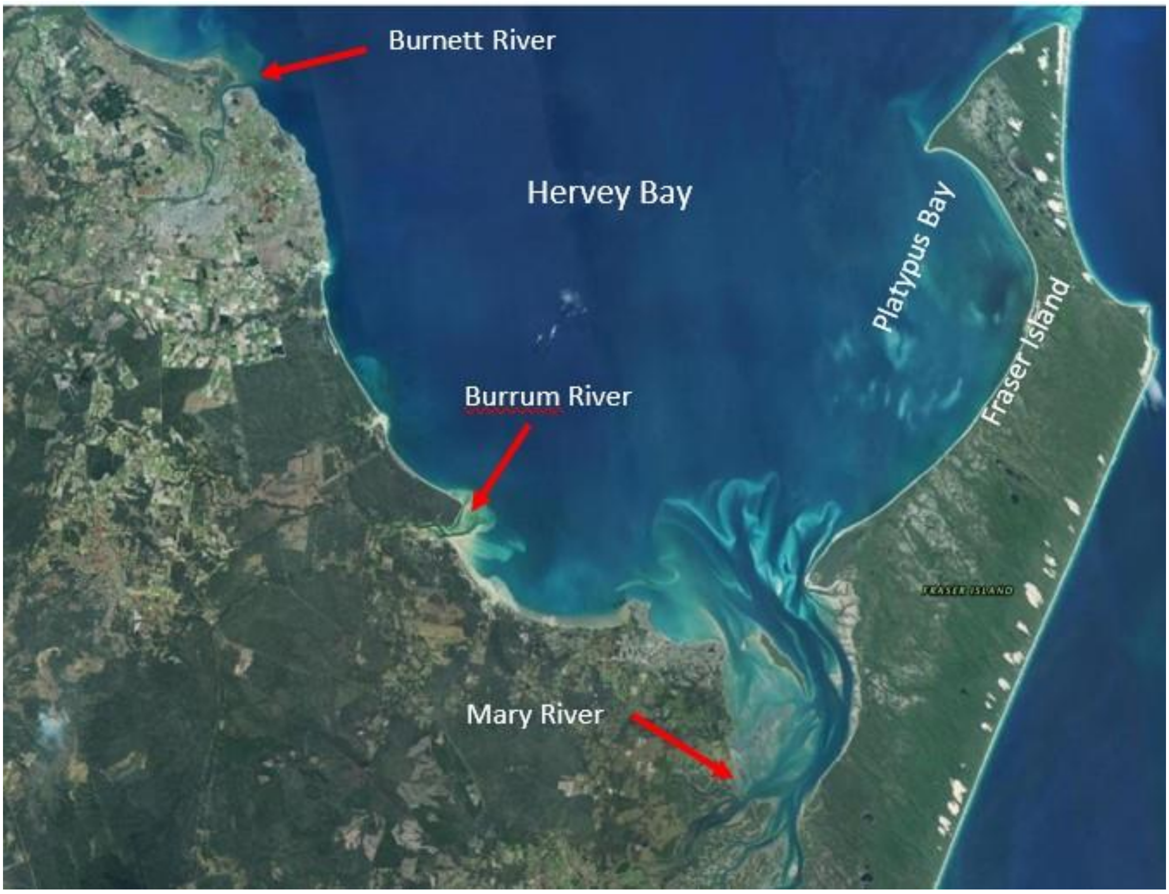

Figure S3. Map of Hervey Bay and Fraser Island with arrows showing the position of the mouths of three major rivers that flow into Hervey Bay, the Burnett, Burrum and Mary rivers. Image adapted from QGlobe (https://qldglobe.information.qld.gov.au/; Creative Commons Attribution 4.0 International (CC BY 4.0) licence).

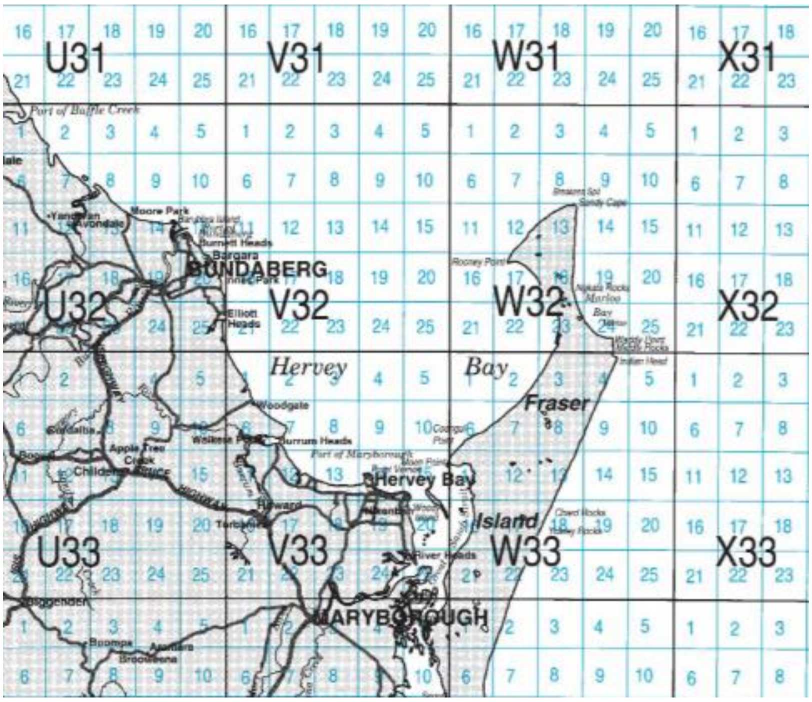

Figure S4. Logbook map grids for the QFish database. QFish is a Queensland Government database that stores and provides information on Queensland fisheries resources and spatial layers. Non-confidential catch and effort data from commercial and charter fisheries can be extracted from the public database. Figure shows the QFish 30 X 30 nm square logbook map grids (U, V, W, and X series) around Fraser Island and Hervey Bay. Fishers record their catch from the smaller 6 x 6 nm grids that make up the larger logbook grids. Platypus Bay is bisected by logbook grids W32 and W33. Queensland commercial fishers must record their catch in logbooks with the data archived in QFish [141 available online: http://qfish.fisheries.qld.gov.au/]. The data that can be publicly retrieved from QFish is available from 30 x 30 nautical mile map grids. Platypus Bay falls in the middle of the two logbook grids, W32 and W33. Grid areas are based on latitude and longitude and do not differentiate between water and land, with a considerable area of both grids covering much of Fraser Island as well as waters to the north and east of Fraser Island. For protection of fisher's right to confidentiality, QFish data for a grid is only publicly available if it can be aggregated from 5 or more fishers. The data in Table 1 is aggregated for fish caught from within any of ten 6 x 6 nautical mile sub-grids that cover only Platypus Bay waters within the two larger logbook grids W32 and W33. The aggregated data from these sub-grids were retrieved by the Queensland Department of Agriculture and Fisheries and is the lowest level of detail that could be provided by the department that meets the confidentiality requirements for fishers. The data was aggregated from sub-grids 16, 17, 21, 22, 23 from logbook grid W32, and sub-grids 1, 2, 3, 6, 7 from logbook grid W33. Rabbitfishes are referred to as "Spinefoot" and "Black Spinefoot" in the QFish database.

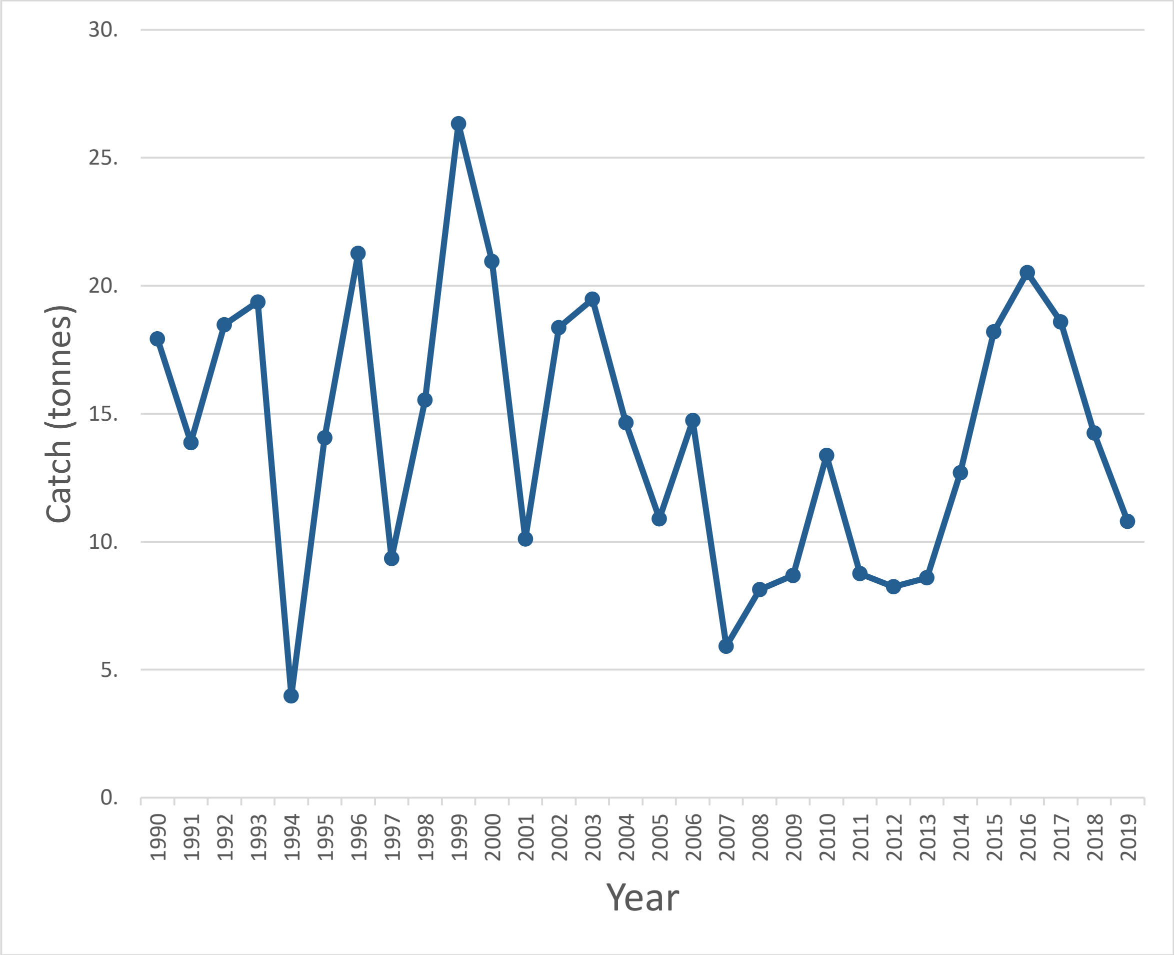

Figure S5. Annual combined catches (tonnes) of Spanish mackerel extracted from QFish 30 × 30 nm logbook grids W32, W32, V32, V33 between 1990 and 2019 (see Figure S4 for map of logbook grids and QFish details).

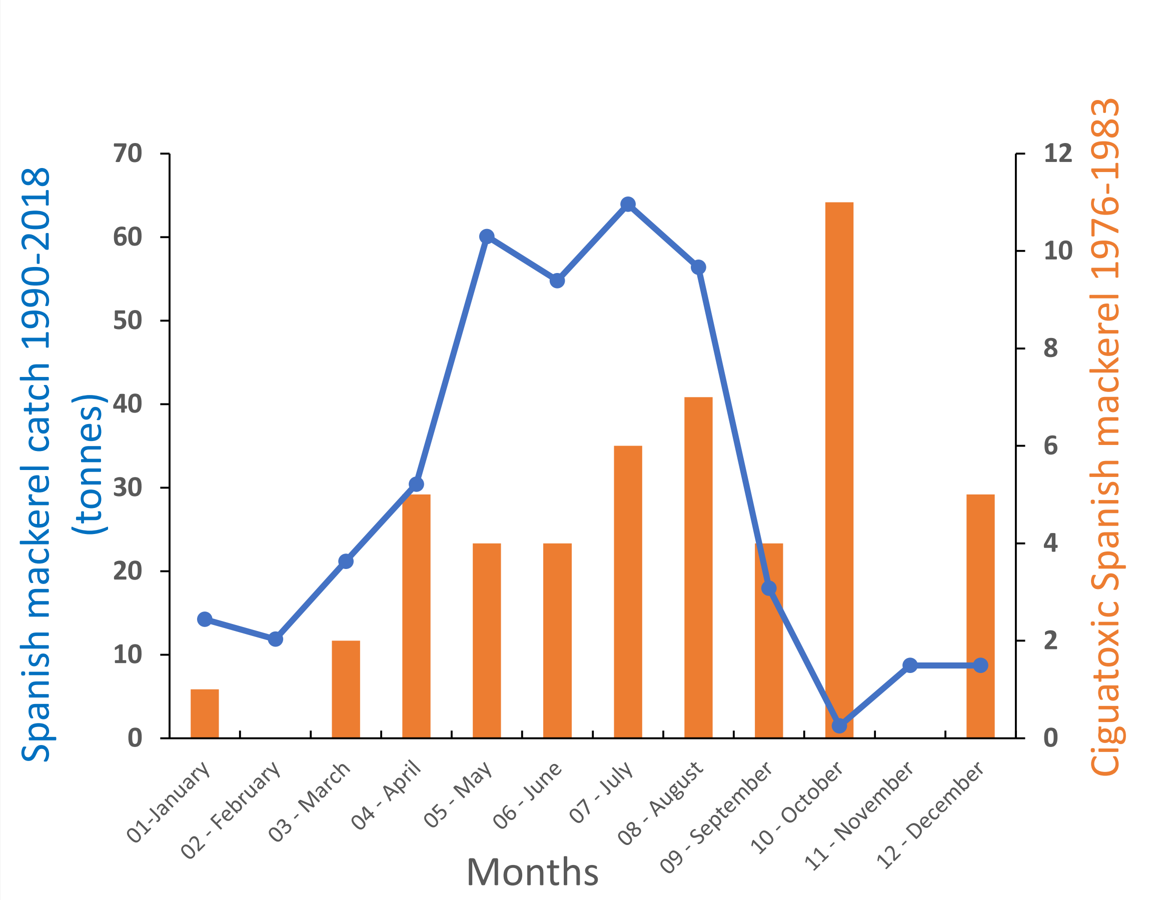

Figure S6. Summed monthly catches of Spanish mackerel extracted from QFish logbook grids W32 and W33 (Figure S4) between 1990 and 2018, and monthly numbers of ciguatoxic Spanish mackerel caught in the Hervey Bay region between 1976 and 1983 [78].

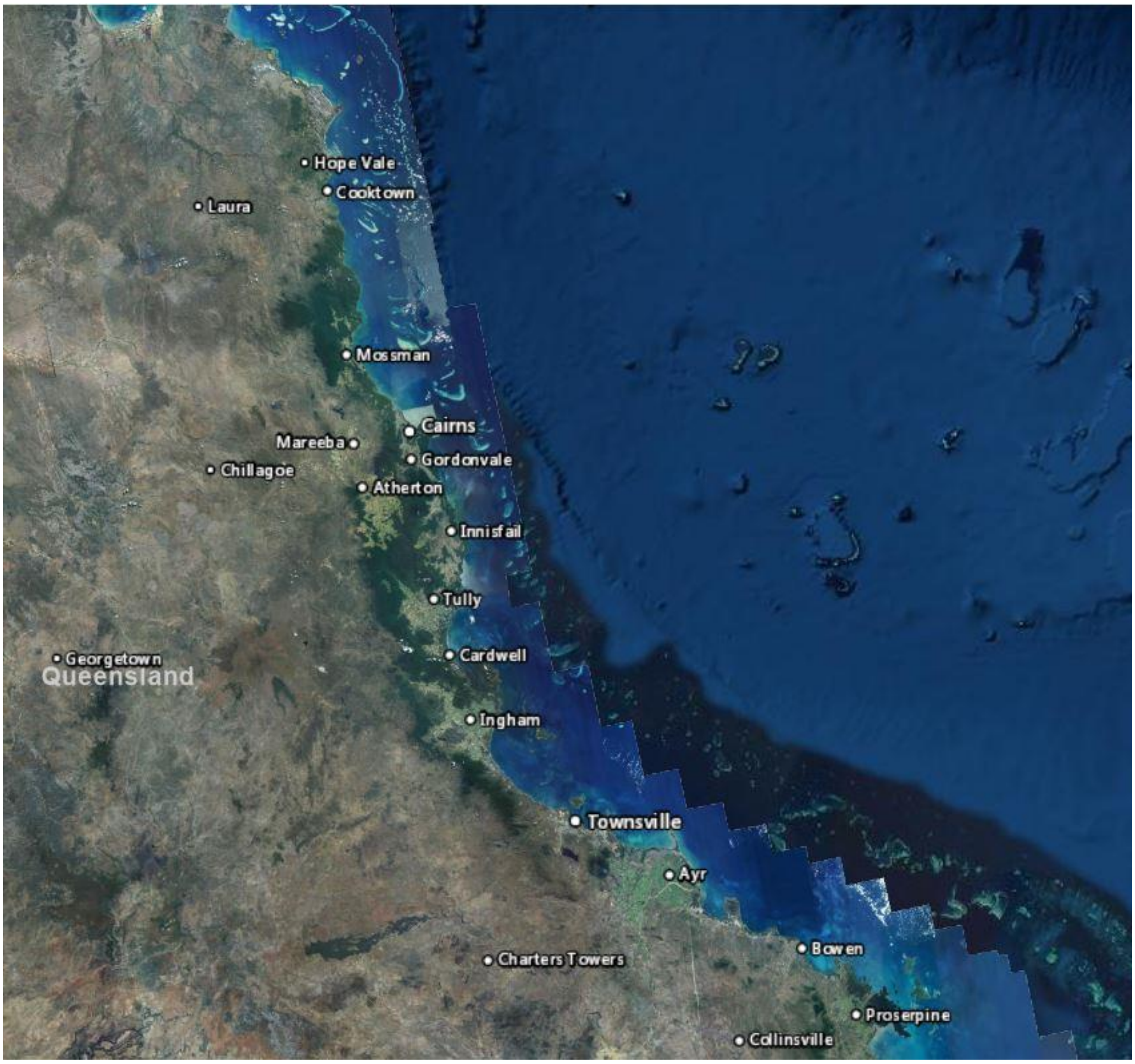

Figure S7. Map showing parts of the central and north Queensland coasts between Proserpine and Cooktown (including the Wet Tropics region from north of Townsville to south of Cooktown). Image from QGlobe (https://qldglobe.information.qld.gov.au/; Creative Commons Attribution 4.0 International (CC BY 4.0) licence).

a

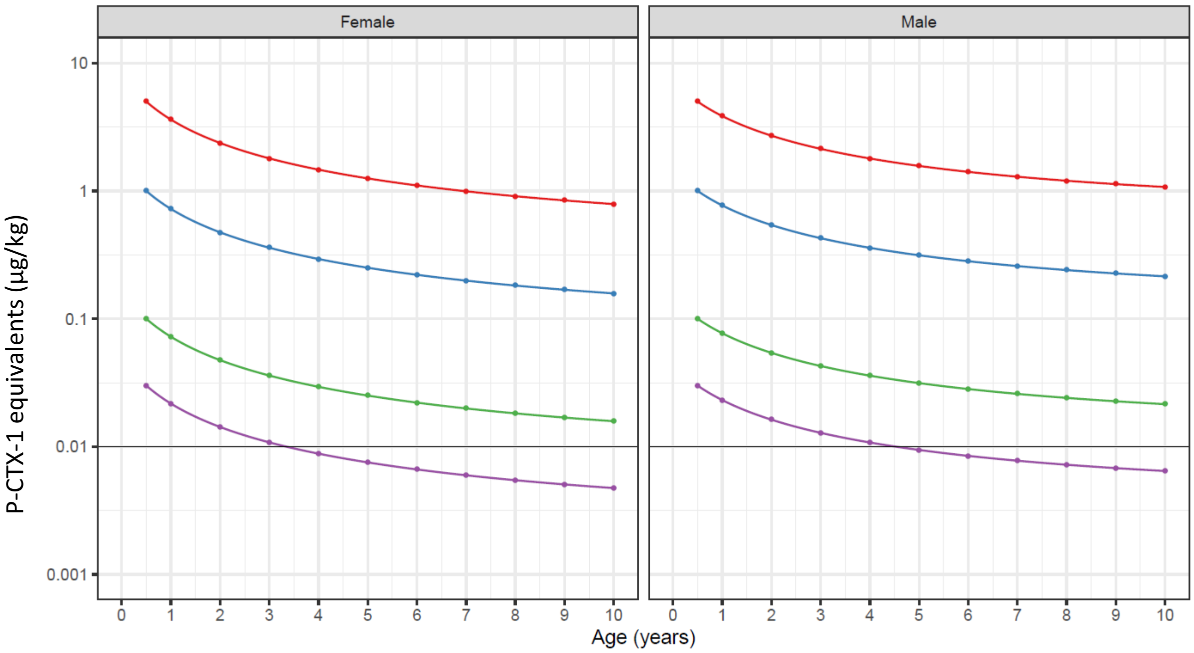
Female
Male
10
1
0.1
0.01
0.001
P-CTX-1 equivalents (µg/kg)
0 1 2 3 4 5 6 7 8 9 10
Age (years)

b

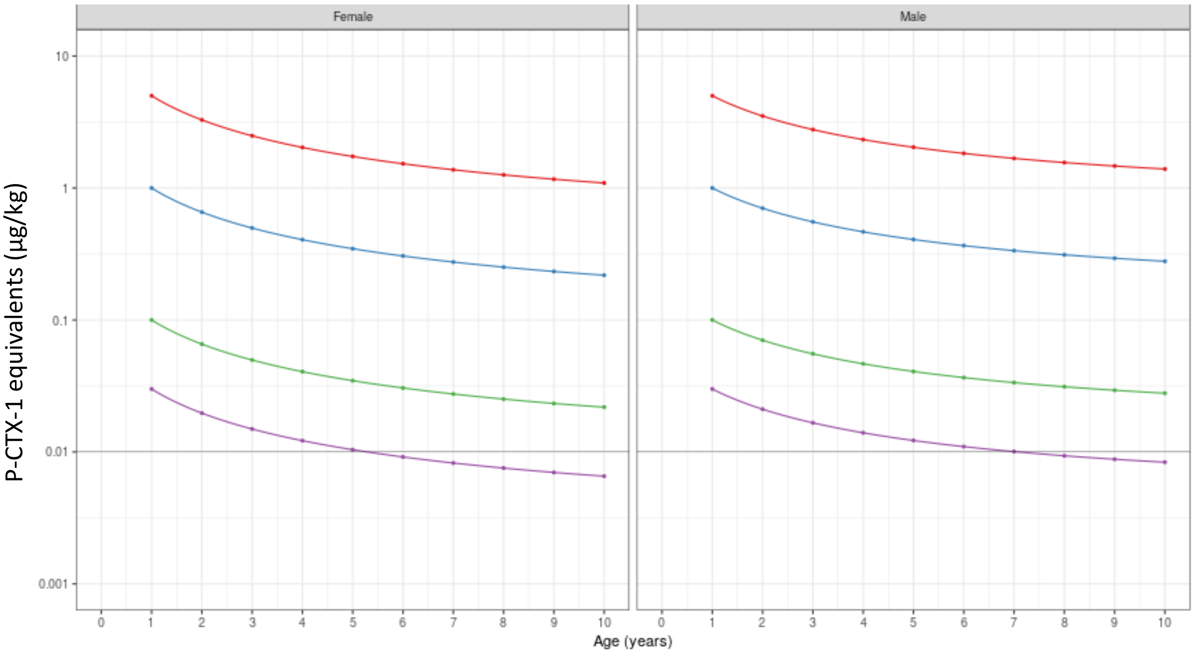
Female
Male
10
1
0.1
0.01
0.001
P-CTX-1 equivalents (µg/kg)
0 1 2 3 4 5 6 7 8 9 10
Age (years)

c

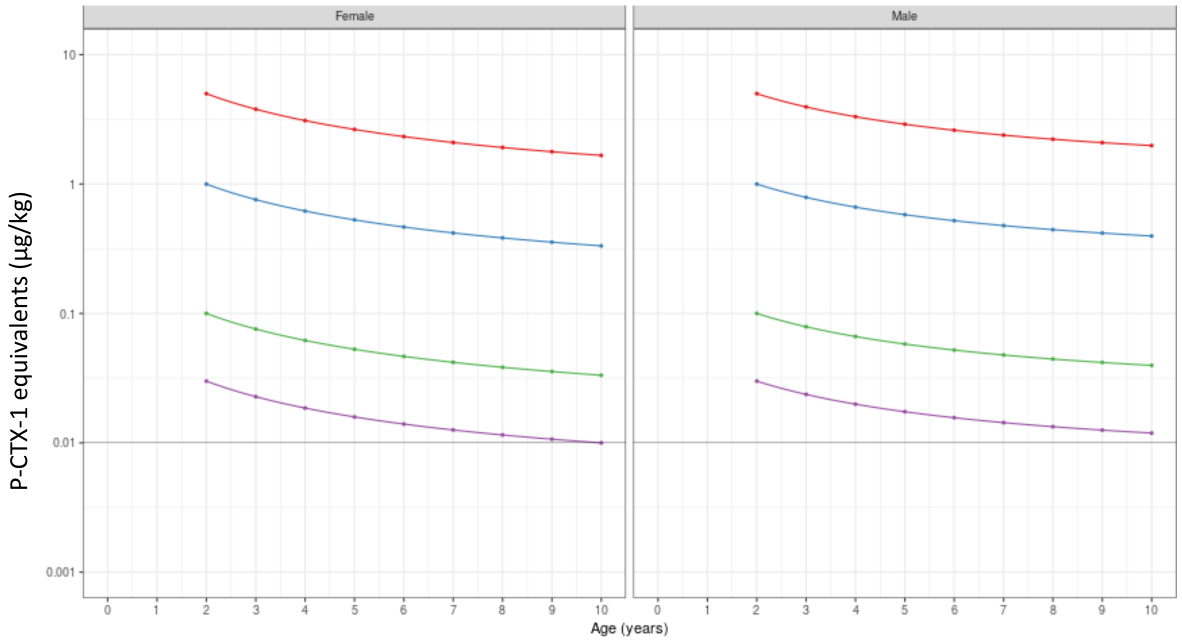

d

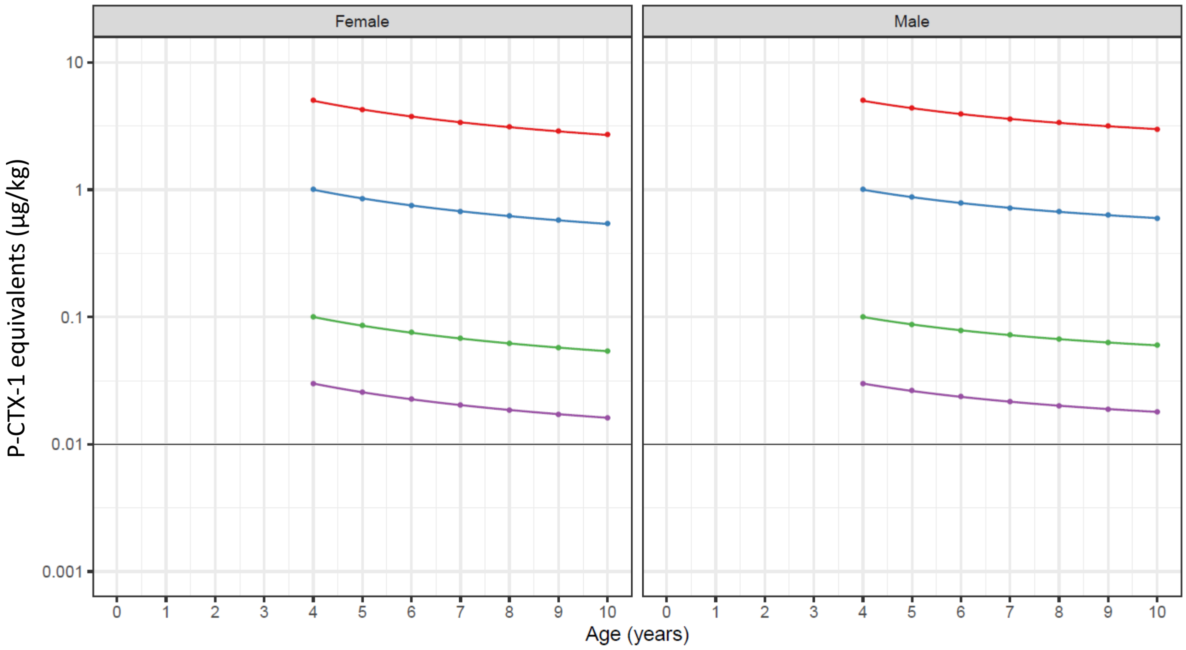

Figure S8. Expanded Figure 6. Modelled dilution of 5.0, 1.0, 0.1 and 0.03 µg/kg P-CTX-1 equivalents from Spanish mackerel flesh by somatic growth, for fish contaminated with ciguatoxins at (**a**) 0.5 years of age, (**b**) 1 year of age, (**c**) 2 years of age, and (**d**) 4 years of age.

a

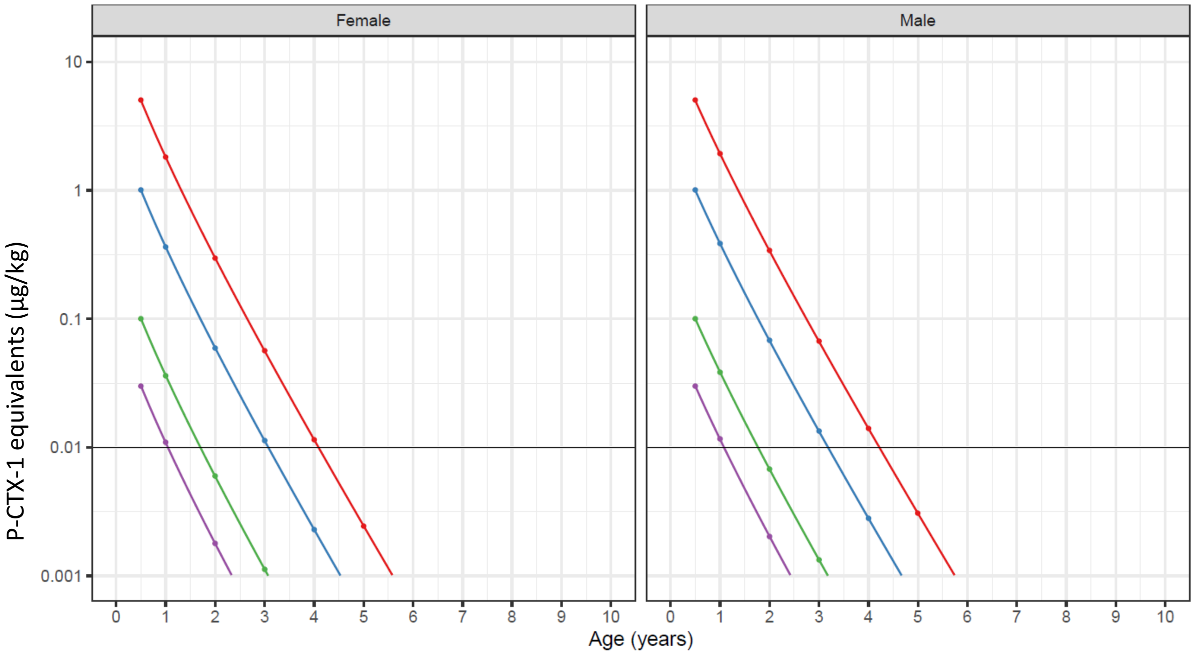
Female
Male
10
1
0.1
0.01
0.001
P-CTX-1 equivalents (µg/kg)
0 1 2 3 4 5 6 7 8 9 10
Age (years)

b

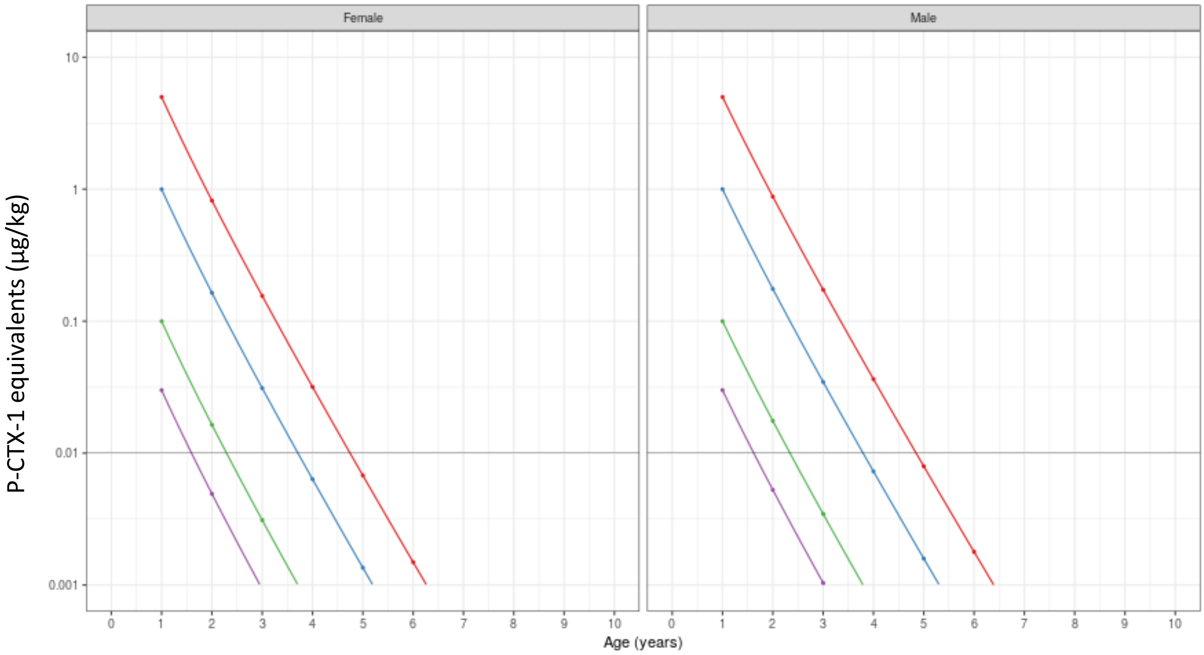
Female
Male
10
1
0.1
0.01
0.001
P-CTX-1 equivalents (µg/kg)
0 1 2 3 4 5 6 7 8 9 10
Age (years)

c

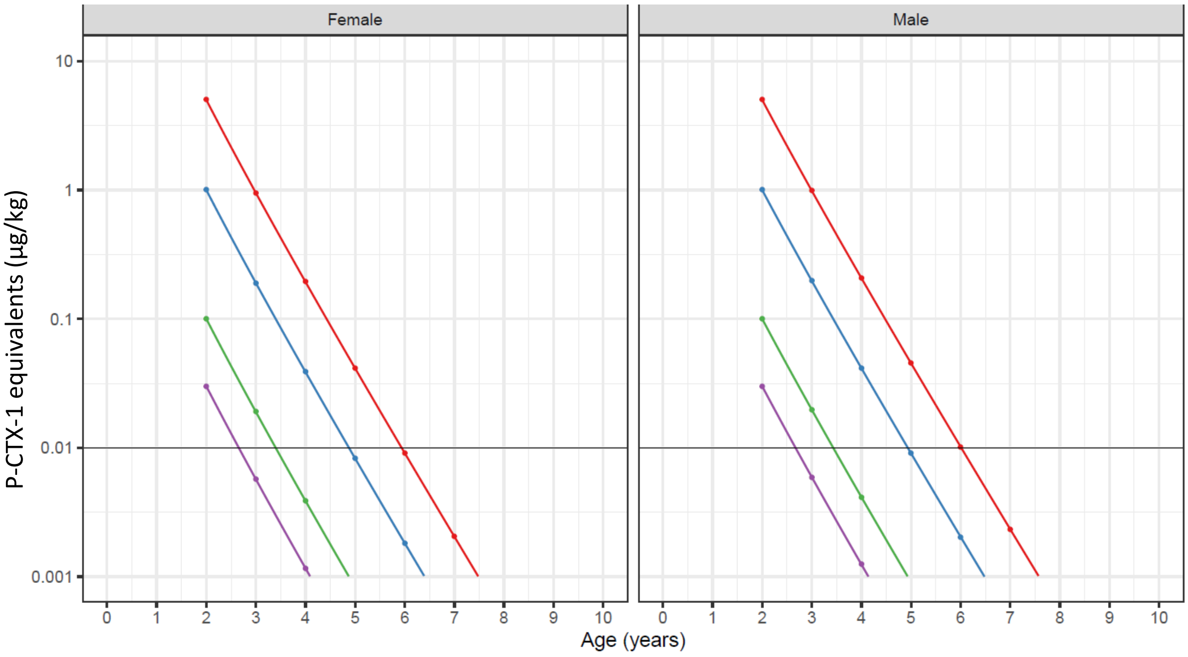

d

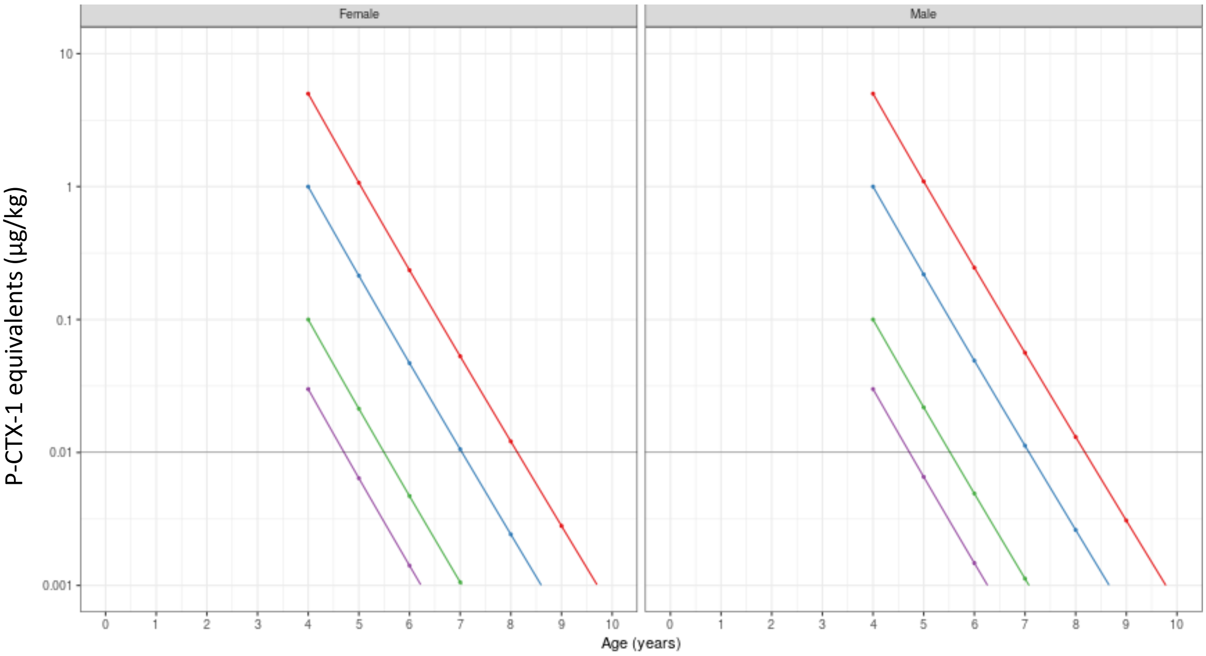

Figure S9. Expanded Figure 7 for depuration half-life = 0.5 year. Modelled dilution of 5.0, 1.0, 0.1, and 0.03 µg/kg P-CTX-1 equivalents from the flesh of female and male Spanish mackerel by a combination of somatic growth and depuration. Modelled are Spanish mackerel contaminated with ciguatoxins at: (**a**) 0.5 year of age, (**b**) 1 year of age, (**c**) 2 years of age (**d**) 4 years of age of age.

a

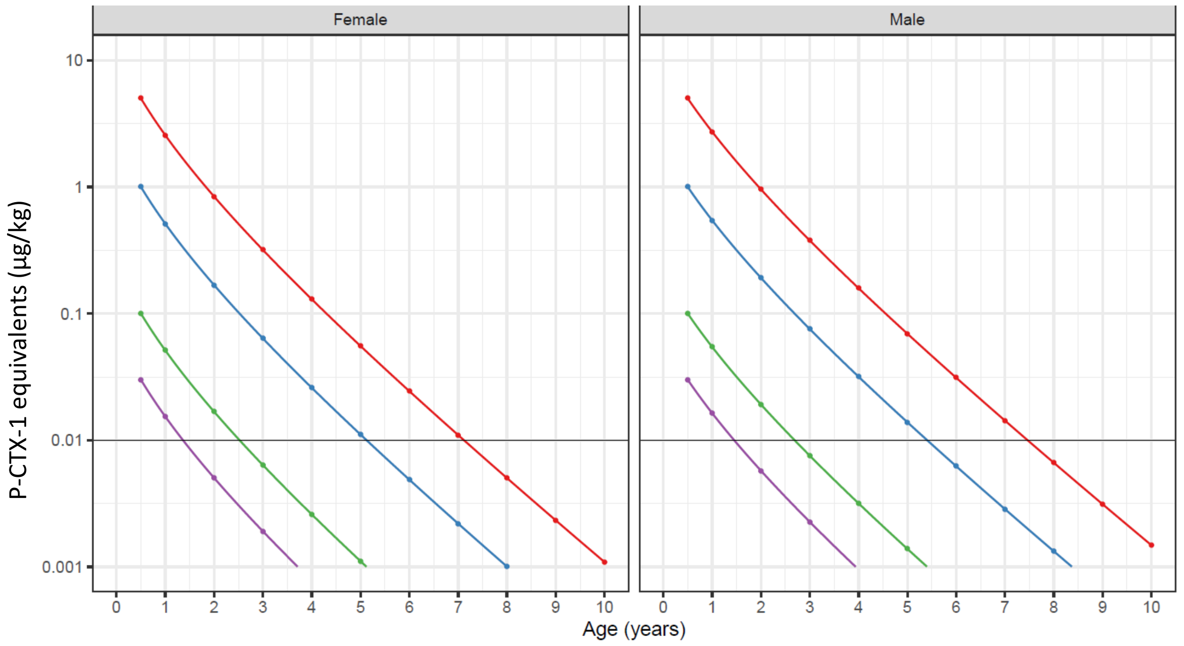
Female
Male
10
1
0.1
0.01
0.001
P-CTX-1 equivalents (µg/kg)
0 1 2 3 4 5 6 7 8 9 10
Age (years)

b

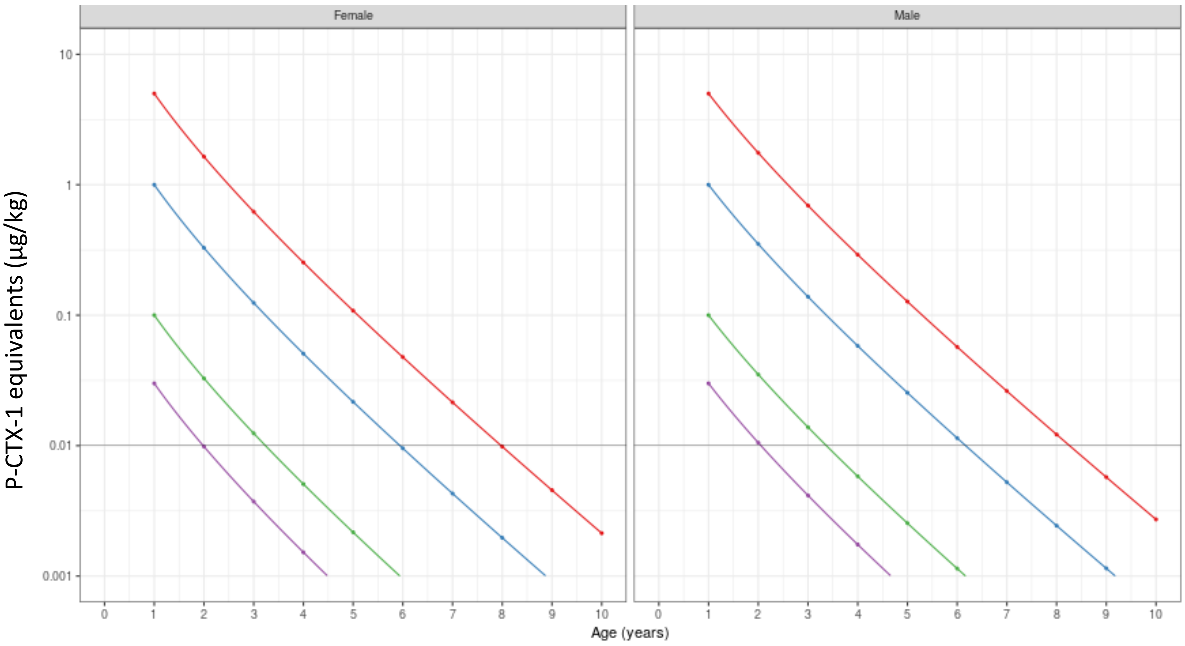
Female
Male
10
1
0.1
0.01
0.001
P-CTX-1 equivalents (µg/kg)
0 1 2 3 4 5 6 7 8 9 10
Age (years)

c

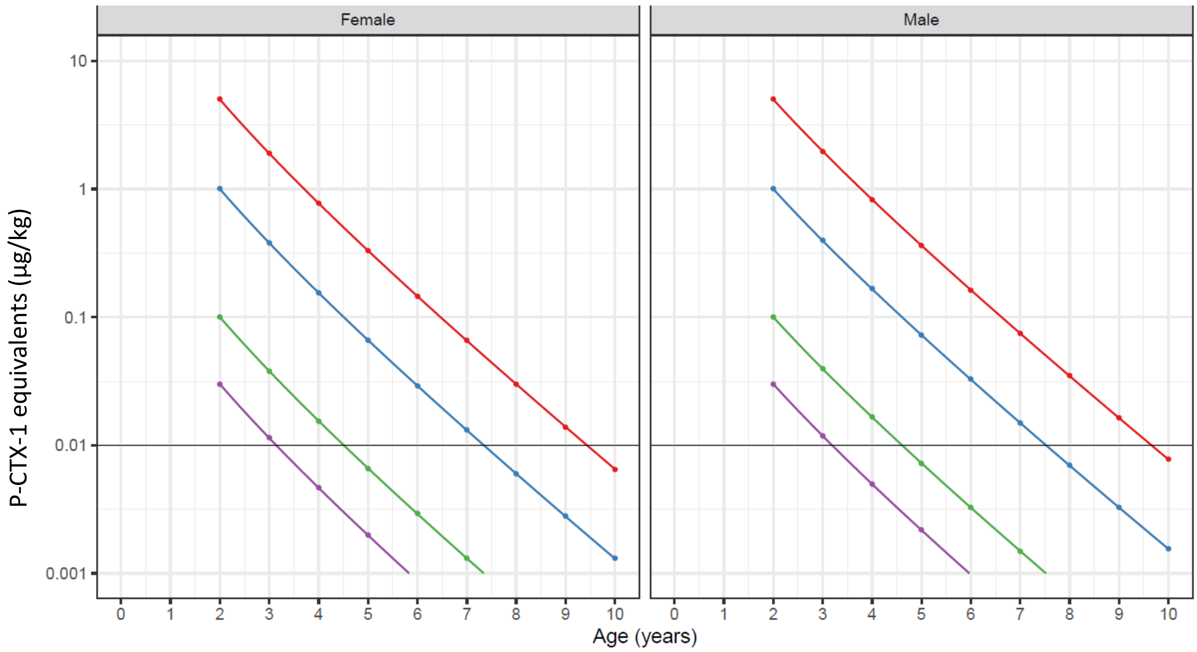

d

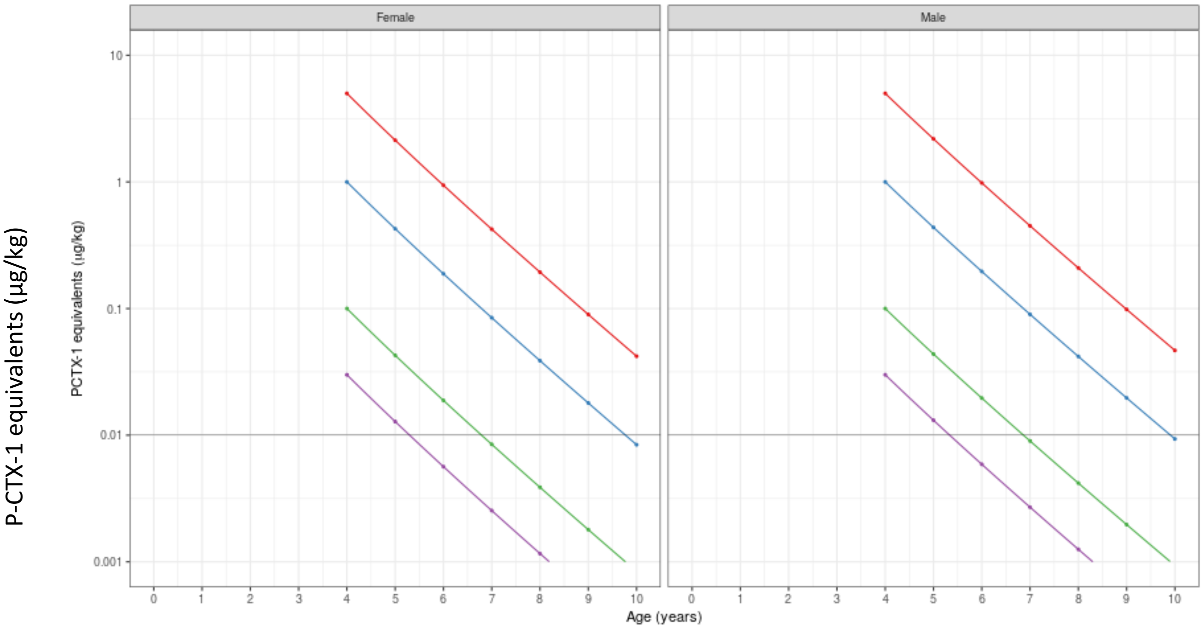

Figure S10. Expanded Figure 7 for depuration half-life = 1.0 year. Modelled dilution of 5.0, 1.0, 0.1, and 0.03 µg/kg P-CTX-1 equivalents from the flesh of female and male Spanish mackerel by a combination of somatic growth and depuration. Modelled are Spanish mackerel contaminated with ciguatoxins at: (**a**) 0.5 year of age, (**b**) 1 year of age, (**c**) 2 years of age (**d**) 4 years of age.

a

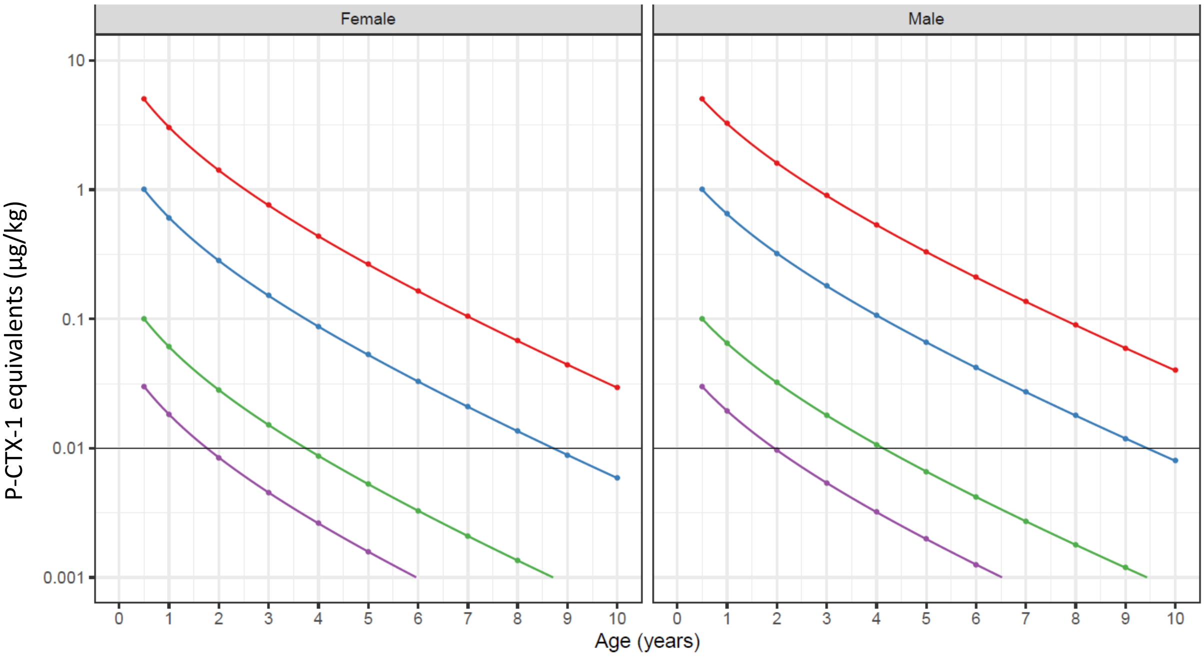
Female
Male
10
1
0.1
0.01
0.001
P-CTX-1 equivalents (µg/kg)
0 1 2 3 4 5 6 7 8 9 10
Age (years)

b

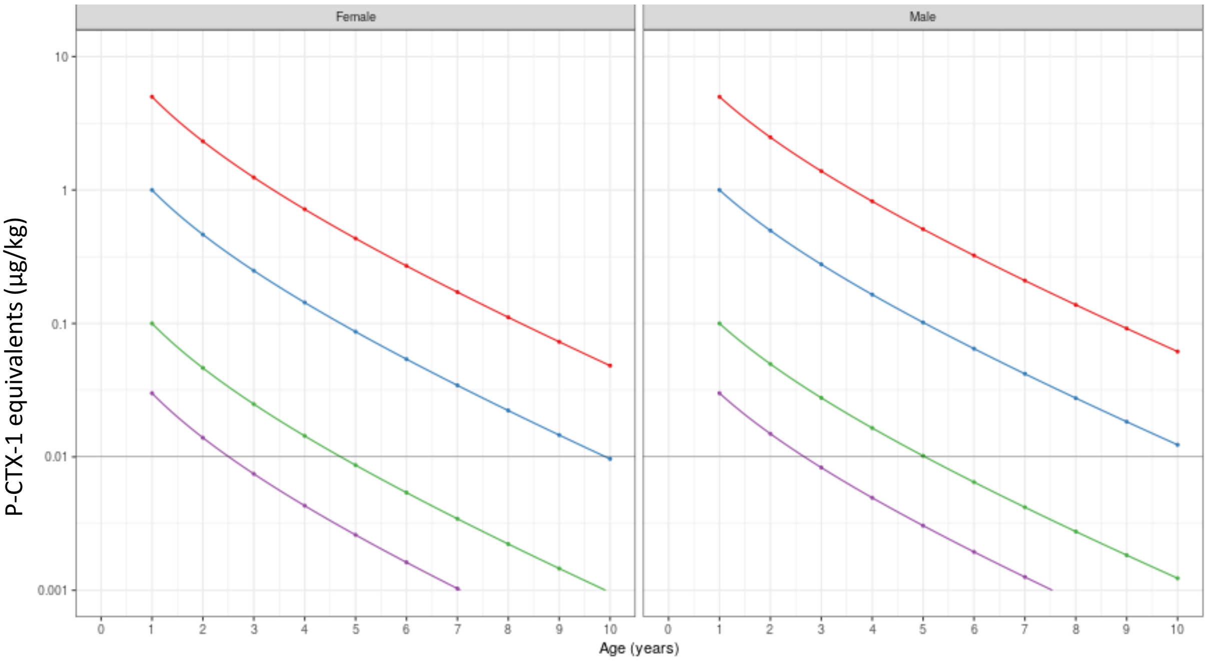
Female
Male
10
1
0.1
0.01
0.001
P-CTX-1 equivalents (µg/kg)
0 1 2 3 4 5 6 7 8 9 10
Age (years)

c

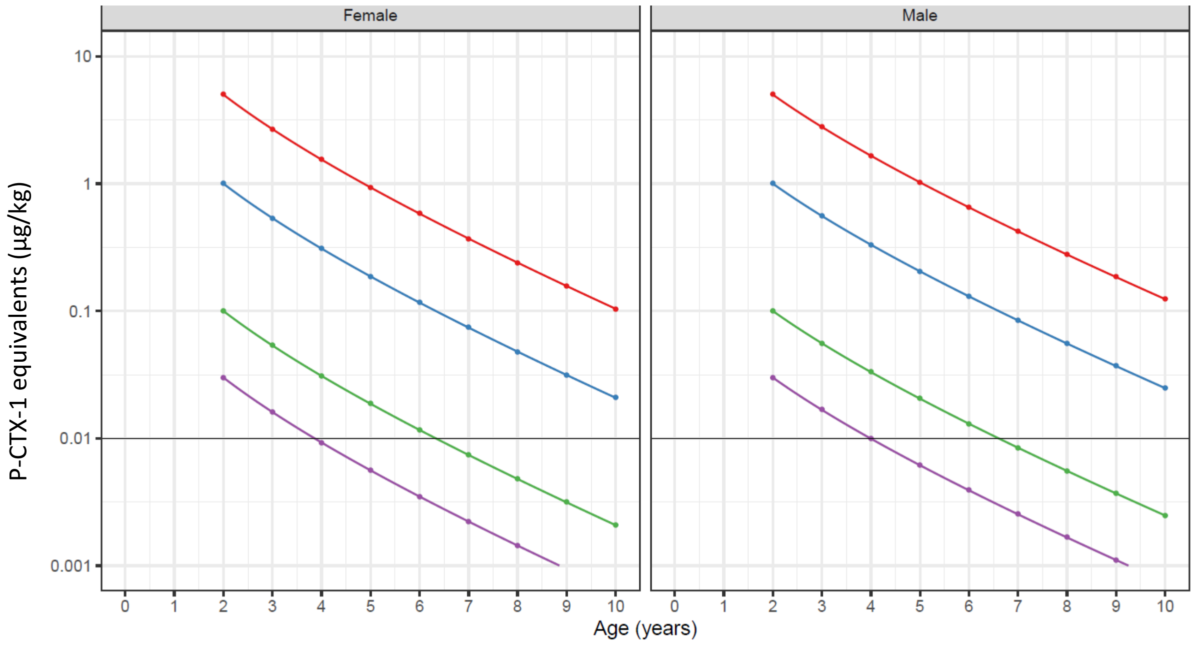

d

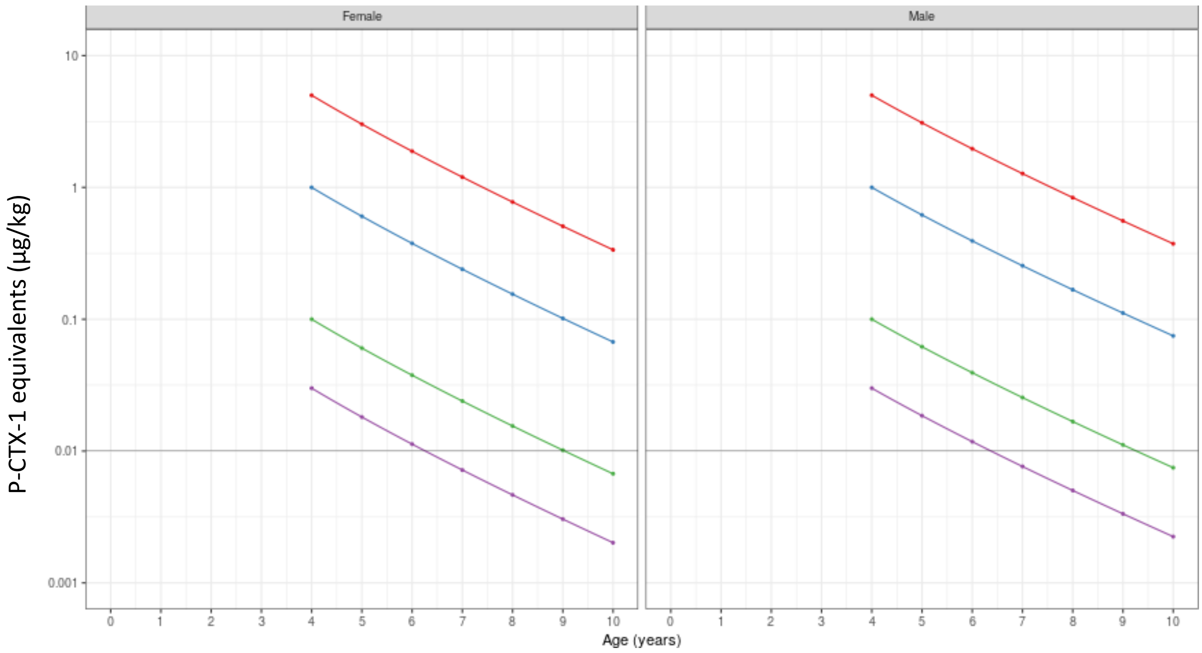

Figure S11. Expanded Figure 7 for depuration half-life = 2.0 years: Modelled dilution of 5.0, 1.0, 0.1, and 0.03 µg/kg P-CTX-1 equivalents from the flesh of female and male Spanish mackerel by a combination of somatic growth and depuration. Modelled are Spanish mackerel contaminated with ciguatoxins at: (**a**) 0.5 year of age, (**b**) 1 year of age, (**c**) 2 years of age (**d**) 4 years of age of age.

a

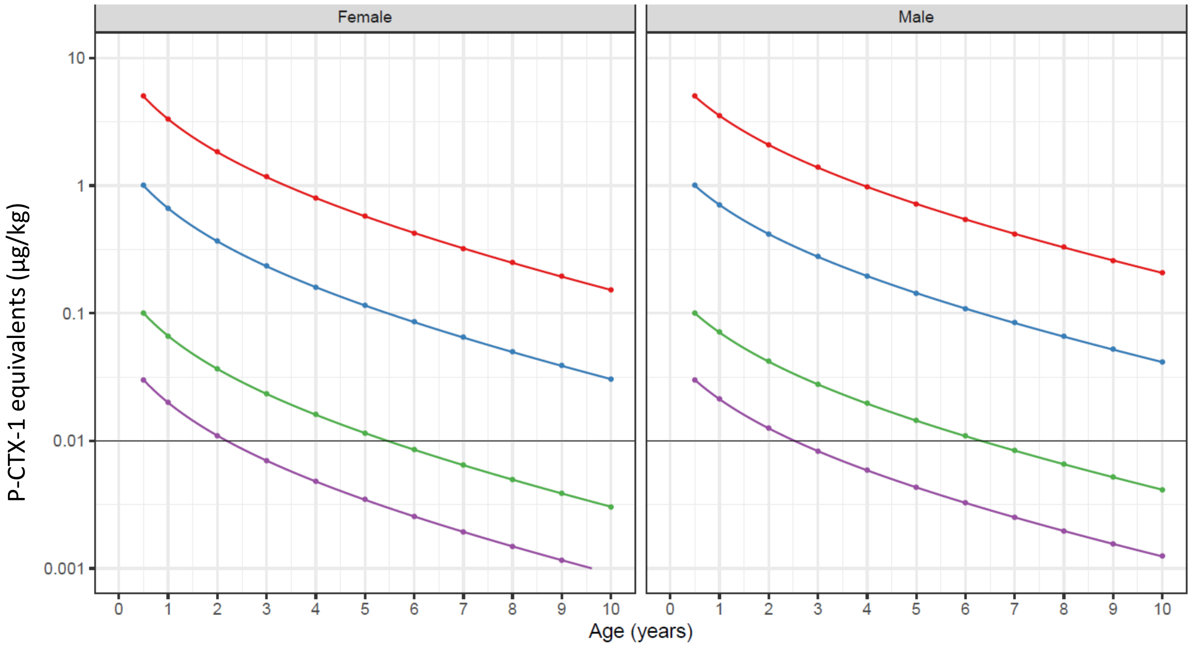
Female
Male
10
1
0.1
0.01
0.001
P-CTX-1 equivalents (µg/kg)
0 1 2 3 4 5 6 7 8 9 10
Age (years)

b

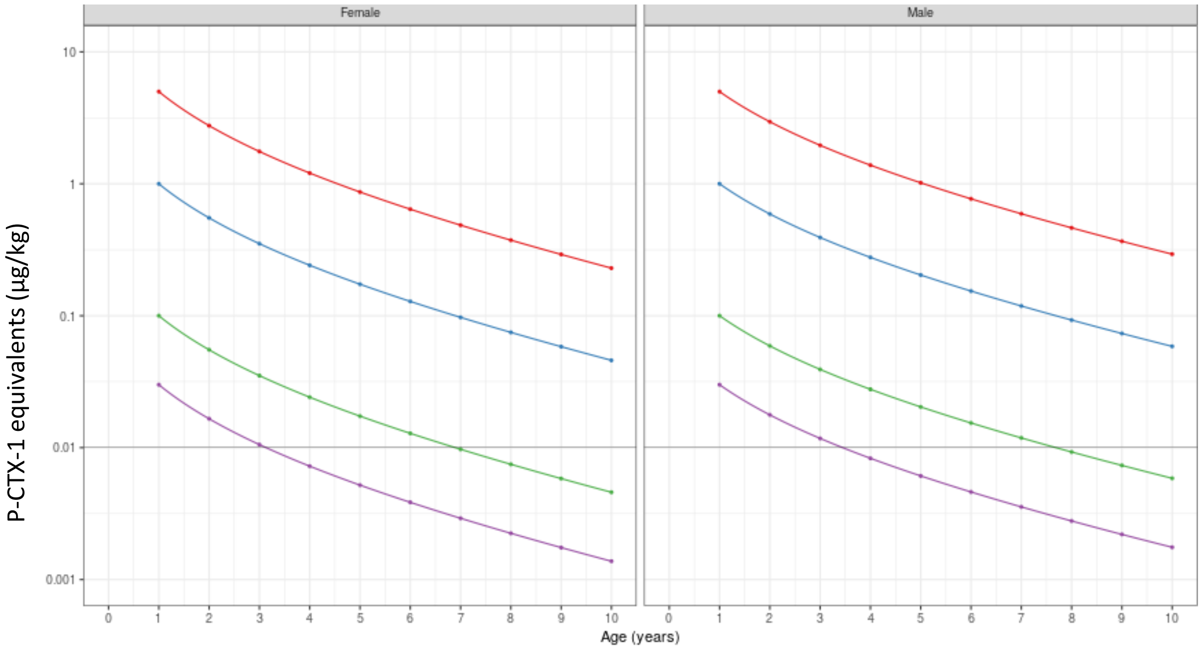
Female
Male
10
1
0.1
0.01
0.001
P-CTX-1 equivalents (µg/kg)
0 1 2 3 4 5 6 7 8 9 10
Age (years)

c

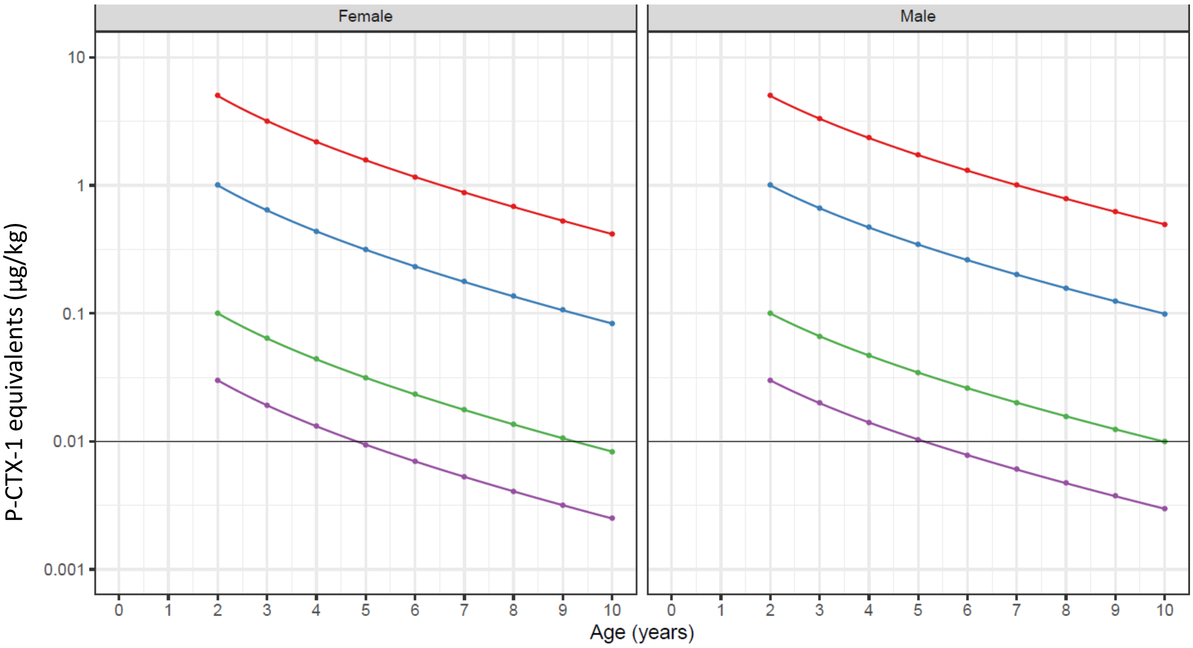

d

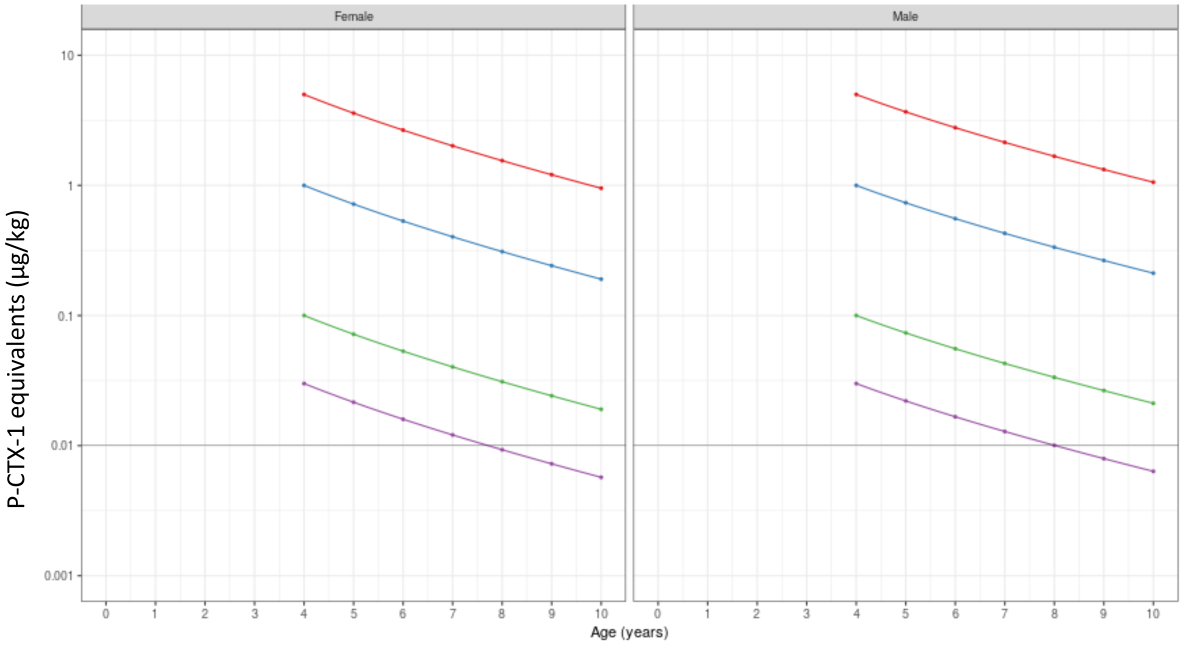

Figure S12. Expanded Figure 7 for depuration half-life = 4.0 years. Modelled dilution of 5.0, 1.0, 0.1, and 0.03 µg/kg P-CTX-1 equivalents from the flesh of female and male Spanish mackerel by a combination of somatic growth and depuration. Modelled are Spanish mackerel contaminated with ciguatoxins at: (**a**) 0.5 year of age, (**b**) 1 year of age, (**c**) 2 years of age, (**d**) 4 years of age of age.

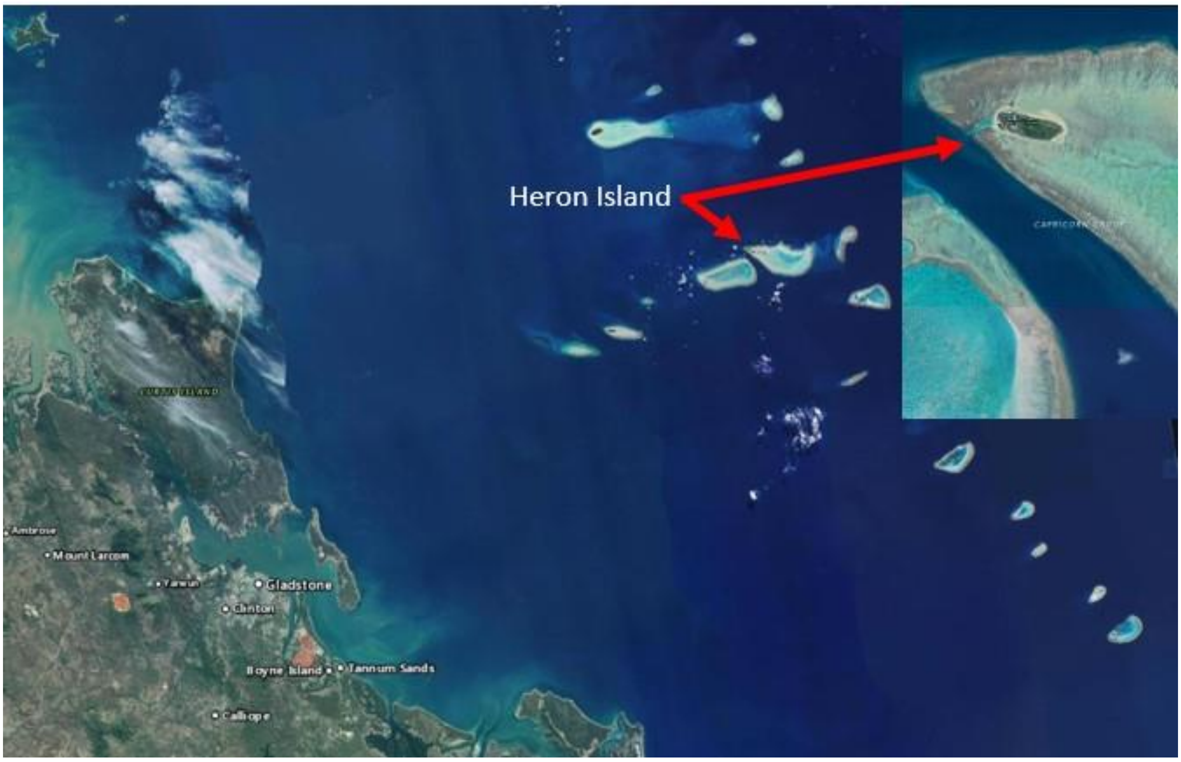

Figure S13. Map showing the position of some of the Capricorn-Bunker group of reefs and coral cays off the coast of Gladstone, Queensland. These are the southernmost reefs in the Great Barrier Reef. The position of Heron Island is indicated by arrows on main image and inset. Image from QGlobe (https://qldglobe.information.qld.gov.au/; Creative Commons Attribution 4.0 International (CC BY 4.0) licence).
